# CD19 CAR-T cells for treatment-refractory autoimmune diseases: the phase 1/2 CASTLE basket trial

**DOI:** 10.1038/s41591-025-04185-6

**Published:** 2026-01-07

**Authors:** Fabian Müller, Melanie Hagen, Andreas Wirsching, Soraya Kharboutli, Michael Aigner, Simon Völkl, Sascha Kretschmann, Koray Tascilar, Jule Taubmann, Laura Bucci, Maria Gabriella Raimondo, Christina Bergmann, Tobias Rothe, Giulia Corte, Carlo Tur, Luis Muñoz, Sebastian Böltz, Louis Schuster, Fabian Hartmann, Panagiotis Garantziotis, Silvia Spörl, Ingrid Vasova, Armin Gerbitz, Bernd Spriewald, Hans Kiener, Diana Giannarelli, Franco Locatelli, Maria-Antonietta D’Agostino, Linda Hanssens, Stefan Miltenyi, Aline Bozec, Ricardo Grieshaber-Bouyer, Andreas Mackensen, Georg Schett

**Affiliations:** 1 Department of Internal Medicine 5 – Hematology and Oncology, Friedrich Alexander Universität (FAU) Erlangen-Nürnberg and Universitätsklinikum Erlangen, Erlangen, Germany; 2 Deutsches Zentrum Immuntherapie, Friedrich Alexander Universität (FAU) Erlangen-Nürnberg and Universitätsklinikum Erlangen, Erlangen, Germany; 3 Department of Internal Medicine 3 − Rheumatology and Immunology, Friedrich Alexander Universität (FAU) Erlangen-Nürnberg and Universitätsklinikum Erlangen, Erlangen, Germany; 4 Department of Rheumatology, Fondazione Policlinico Universitario A. Gemelli, IRCCS, Rome, Italy; 5 Department of Internal Medicine 3, Medical University of Vienna, Vienna, Austria; 6 Biostatistical Unit, Fondazione Policlinico Universitario A. Gemelli IRCCS, Rome, Italy; 7 Department of Pediatric Hematology/Oncology and Cell & Gene Therapy, IRCCS Bambino Gesu Children’s Hospital, Catholic University of the Sacred Heart, Rome, Italy; 8 Catholic University of Sacred Heart, Rome, Italy; 9 Miltenyi Biomedicine, Bergisch Gladbach, Germany; 10 Karolinska Institutet and Karolinska University Hospital, Stockholm, Sweden

**Keywords:** Systemic lupus erythematosus, Immunotherapy, Idiopathic inflammatory myopathies, Autoimmune diseases, Systemic sclerosis

## Abstract

Chimeric antigen receptor (CAR)-T cells are considered a powerful therapeutic tool to reset the immune system in patients with autoimmune diseases. Innovative trial designs are needed to allow feasible testing of the safety and efficacy of CAR-T cells in clinical studies. CASTLE (CAR-T cells in systemic B cell mediated autoimmune disease) is a phase 1/2a two-stage optimal design basket study that investigated the safety and efficacy of zorpocabtagene autoleucel (Zorpo-cel, also known as MB-CART19.1), an autologous CD19 CAR-T cell product, in patients with treatment-resistant systemic lupus erythematosus (SLE), systemic sclerosis (SSc) and idiopathic inflammatory myopathies (IIM). The primary safety outcome was the rate of cytokine release syndrome (CRS) and immune effector cell-associated neurotoxicity syndrome (ICANS). The secondary clinical efficacy outcomes were remission of SLE according to DORIS criteria, no progression of interstitial lung disease in SSc and American College of Rheumatology (ACR) major/moderate response in IIM after 24 weeks. A total of 24 patients were enrolled (10 with SLE, 9 with SSc and 5 with IIM), all receiving a single infusion of Zorpo-cel after stopping immunosuppressive treatments and receiving standard lymphodepletion with cyclophosphamide and fludarabine. Primary and secondary endpoints of CASTLE were met. Regarding safety, no CRS higher than grade 2 and no ICANS occurred. Regarding efficacy, 22 of the 24 patients achieved predefined efficacy endpoints, with 9 out of 10 patients with SLE reaching DORIS remission, 9 out of 9 patients with SSc showing no disease progression, and 4 out of 5 patients with IIM reaching ACR major/moderate response. Furthermore, all patients remained free of glucocorticoids and any other immunosuppressive treatment over the entire observation period of 24 weeks. CASTLE suggests the feasibility, safety and efficacy of Zorpo-cel in three different autoimmune diseases and paves the way for conducting a pivotal study. ClinicalTrials.gov identifier: NCT06347718, EudraCT identifier: 2022-001366-35.

## Abstract (web summary)

In the phase 1/2 CASTLE basket trial, autologous CD19 CAR-T cell therapy in patients with treatment-refractory systemic lupus erythematosus, systemic sclerosis or idiopathic inflammatory myopathy was safe, with improved disease activity and patient-reported global health in most patients.

## Main

Autoimmune diseases, such as SLE, SSc and IIM, are characterized by a breach in immune tolerance in conjunction with an immune response against intracellular, especially nuclear, antigens, triggering a chronic multiorgan inflammatory disease process^1–3^. SLE, SSc and IIM can be life-threatening owing to affection of vital organs, warranting early and effective therapeutic intervention to prevent permanent organ damage. Although numerous targeted drug therapies have been developed to manage SLE, IIM and SSc, along with traditional broad-spectrum immunosuppressive drugs, these interventions do not usually eliminate the root of the autoimmune disease but necessitate chronic, often lifelong, treatment. Furthermore, a substantial number of patients with SLE, SSc and IIM do not experience sufficient control of their disease activity due to inefficacy or poor tolerance of therapy.

B cells are major drivers in the pathogenesis of autoimmune diseases, as they are involved in antigen presentation, cytokine release, autoantibody production and immune complex formation^4–6^. In accordance with the central role of B cells in autoimmune disease, B-cell-depleting monoclonal antibodies, such as rituximab, ofatumumab, ocrelizumab and obinutuzumab, have shown efficacy^7–9^. However, these protein-based B cell depleters usually require continuous administration to control the disease, as they only rarely induce drug-free remission with true reset of the immunopathology, which is the ultimate goal in treating autoimmune diseases. To achieve durable remission, deep depletion of B cells in the tissues may be needed, which can be achieved by CAR-T cells directed against the B cell repertoire^10^. Hence, small case series have shown that CD19 CAR-T cells can induce durable drug-free remission in autoimmune diseases, such as SLE, IIM and SSc^11–15^. In particular, patients with a severe and treatment-refractory course seem to benefit substantially from CD19 CAR-T cell therapy, providing control of organ manifestations with an acceptable safety profile.

Zorpo-cel is an autologous T cell product genetically engineered using a lentiviral vector to express a CAR that targets the B-lineage antigen CD19. To test the feasibility, safety and efficacy of Zorpo-cel, we conducted the first, to our knowledge, phase 1/2a basket trial in patients with SLE, IIM and SSc who showed severe treatment-refractory disease.

## Results

### Patient characteristics

**Fig. 1:**
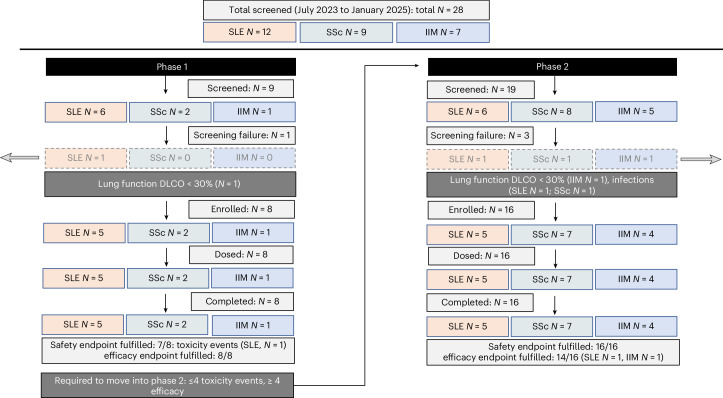
CONSORT flow diagram for screening and enrollment. Flowchart showing the total and per-disease number of patients screened and enrolled into the CASTLE study. All patients received Zorpo-cel.

**Extended Data Fig. 1:**
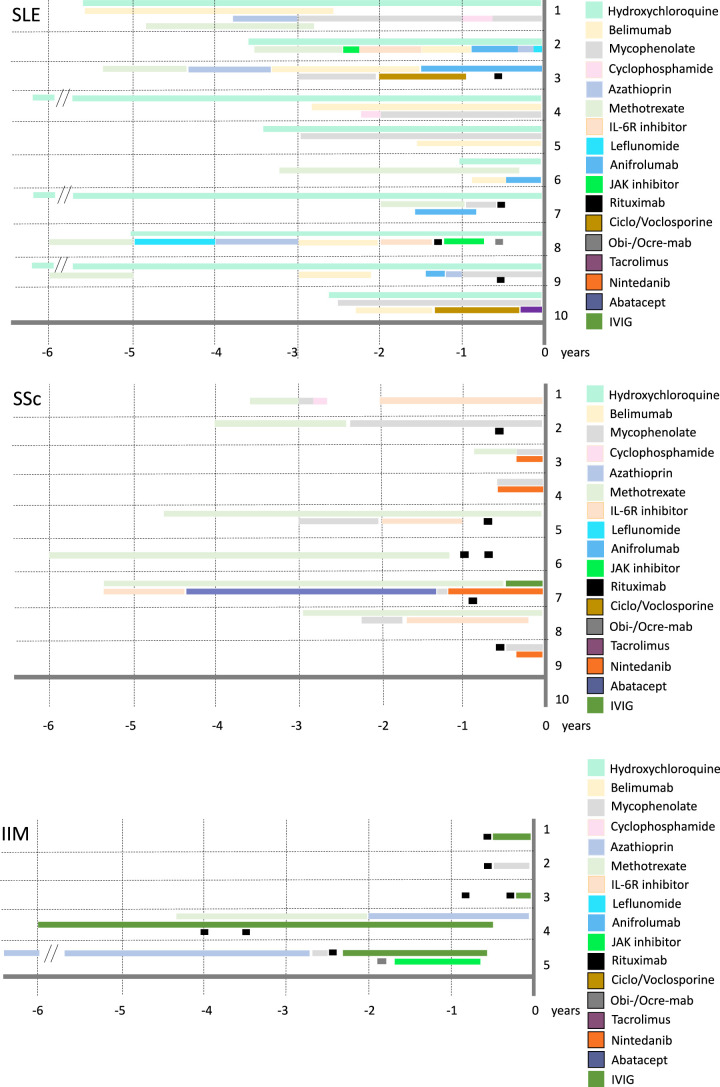
Sequence of previous treatments. Summary of previous immunomodulatory treatments in patients with summary of product characteristics in the entire population and in patients with SLE (top), SSc (middle) and IIM (bottom).

CASTLE is a phase 1/2a basket study investigating the safety and efficacy of autologous CD19 CAR-T cell therapy in patients with severe and treatment-refractory forms of SLE, SSc and IIM. Although its primary endpoint is safety, clinical efficacy is the key secondary endpoint. Exploratory endpoints were the changes in B cell receptor repertoire and the therapy-induced alterations of B cell compartments. The study is based on a two-stage optimal design according to Bryant and Day, which is only able to proceed from phase 1 to phase 2 if predefined safety (≤4 toxicity events) as well as efficacy thresholds (≥4 responders) are met. CASTLE included 8 patients in its first phase and another 16 patients into its second phase. A total of 28 patients with SLE, IIM and SSc were screened, and 24 patients were enrolled into CASTLE between 17 July 2023 and 20 January 2025 (Fig. 1). Screening failures occurred in four patients, two due to concomitant infections (*Salmonella* spp. and *Cryptococcus* spp.) and another two due to severely impaired lung function (diffusing capacity of the lungs for carbon monoxide (DLCO) < 30%). Ten patients had SLE, nine had SSc and five had IIM. Over half (17 out of 24) were women. The median (interquartile range (IQR)) age of patients was 39 (26−53) years, and median disease duration was 4.0 (1.3−6.5) years. Before enrollment into the study, the patients had experienced failure of response or intolerance to a median of four (3−6) immunosuppressive treatments. Patients had active disease with a median SLEDAI score (SLE disease activity index) of 12 (7.8−16.5) points in SLE, a median mRSS (modified Rodnan skin score) of 26 (17−30.5) in SSc, and a median MMT-8 (manual muscle testing 8) score of 117 (75.5−138) in IIM. Detailed patient characteristics, including their disease burden, organ involvement and baseline laboratory characteristics, are provided in Table 1. Types and times of previous immunosuppressive treatments in each individual patient are summarized in Extended Data Fig. 1.

**Table 1:** Baseline characteristics of the patients

*N*	All	SLE	SSc	IIM
	24	10	9	5
Demographic characteristics				
Age (years), median (IQR)	39 (26−53)	31 (24−55)	36 (27−56)	44 (45−69)
Sex (*N* female (%))	17 (71)	7 (70)	6 (67)	4 (80)
Disease-specific characteristics				
Disease duration (years)	4.0 (1.3−6.5)	5.0 (2.5−9.0)	2.5 (0.9−5.0)	2.3 (0.9−9.3)
Previous therapies (*N*), median (IQR)	4.0 (3.0−6.0)	5.5 (5.0−8.2)	3.0 (2.5−4.0)	2.0 (2.0−5.5)
SLEDAI (U), median (IQR)	-	11 (6.0−14.0)	-	-
mRSS (U), median (IQR)	-	-	26 (17−30.5)	-
MMT-8 (U), median (IQR)	-	-	-	117 [75.5−138]
VAS global (mm), median (IQR)	63 (53−75)	62 (54−68)	63 (58−76)	51 (42−83)
VAS physician (mm), median (IQR)	68 (54−75)	63 (56−73)	74 (61−84)	54 (45−65)
FACIT (U), median (IQR)	26 (13−48)	19 (11−40)	27 (11−48)	34 (18−48)
HAQ (U), median (IQR)	1.1 (0.1−2.0)	0.7 (0.1−1.4)	1.1 (0.3−2.1)	2.3 (0.4−2.6)
Organ involvement				
Major organs involved, *N* (%)	4.0 (3.0−4.0)	4.0 (4.0−5.0)	4.0 (3.0−5.0)	2.0 (2.0−4.0)
Skin, *N* (%)	22 (92%)	10 (100%)	9 (100%)	3 (60%)
Kidneys, *N* (%)	8 (33%)	8 (80%)	0 (0%)	0 (0%)
Lungs, *N* (%)	14 (58%)	3 (30%)	9 (100%)	2 (40%)
Heart, *N* (%)	7 (29%)	3 (30%)	4 (44%)	0 (0%)
Muscle, *N* (%)	9 (38%)	0 (0%)	4 (44%)	5 (100%)
Joints, *N* (%)	11 (46%)	7 (70%)	2 (22%)	2 (40%)
Gastrointestinal, *N* (%)	9 (38%)	1 (10%)	8 (89%)	0 (0%)
Hematology, *N* (%)	4 (17%)	4 (40%)	0 (0%)	0 (0%)
Serositis, *N* (%)	7 (29%)	7 (70%)	0 (0%)	0 (0%)
Laboratory parameters				
WBC (cells/μl × 10^−3^), median (IQR)	6.6 (4.5−9.0)	4.6 (4.0−6.8)	7.7 (6.7−10.0)	6.3 (5.3−9.7)
B cells (cells/μl), median (IQR)	45 (3−130)	43 (13−78)	39 (4−136)	50 (2−159)
IgG (mg dl^−1^), median (IQR)	1,051 (780−1,252)	818 (701−1,218)	1,069 (929−1,187)	1,257 (813−1,312)
IgA (mg dl^−1^), median (IQR)	168 (127−271)	168 (121−285)	153 (131−206)	212 (77−524)
IgM (mg dl^−1^), median (IQR)	80 (66−133)	66 (45−85)	120 (78−148)	80 (47−143)
CRP (mg l^−1^), median (IQR)	5.8 (2.7−25.6)	14.2 (4.5−27.6)	6.2 (3.2−81.0)	2.4 (0.7−4.2)
IL-6 (pg ml^−1^), median (IQR)	6.3 (3.6−10.7)	8.4 (4.5−15.3)	6.4 (3.5−44.1)	3.7 (1.9−5.1)
Ferritin (ng ml^−1^), median (IQR)	141 (87−219)	171 (114−500)	147 (78−208)	126 (63−201)

HAQ, health assessment questionnaire; VAS, visual analog scale; WBC, white blood cells.

### In vitro and in vivo CAR-T cell expansion

**Fig. 2:**
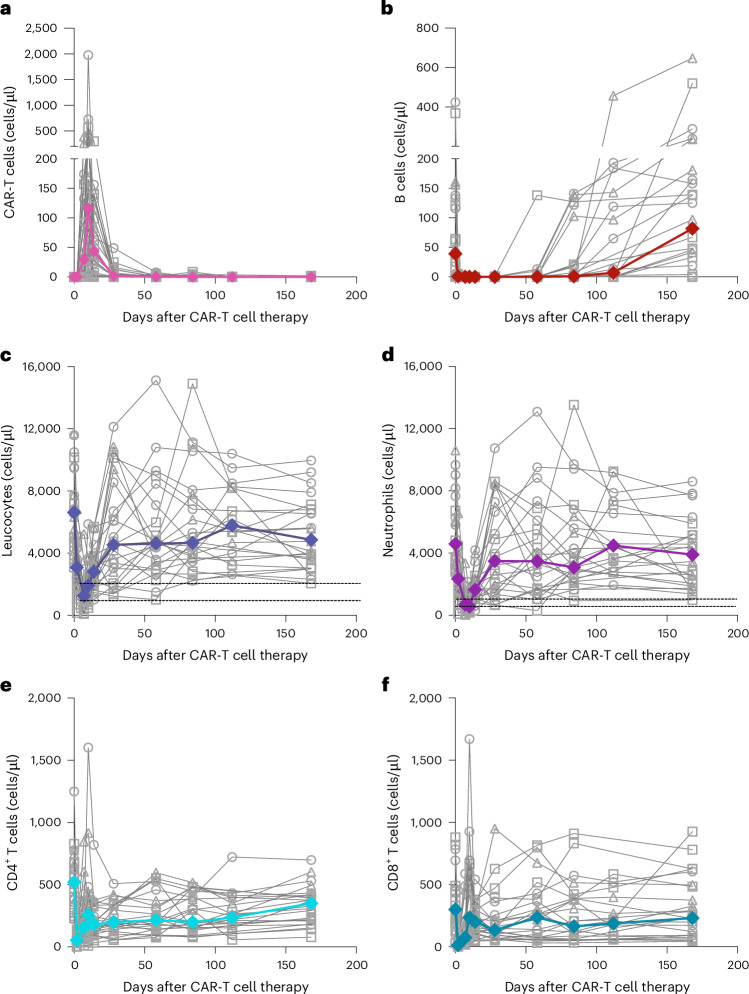
Dynamics of CAR-T cell expansion and effects on leukocyte lineages. **a**–**f**, Dynamics of CAR-T cells (**a**), B cells (**b**), leukocytes (**c**), neutrophils (**d**), CD4^+^ T cells (**e**) and CD8^+^ T cells (**f**). Bold colored lines show medians of individual patients (gray lines).

**Extended Data Fig. 2:**
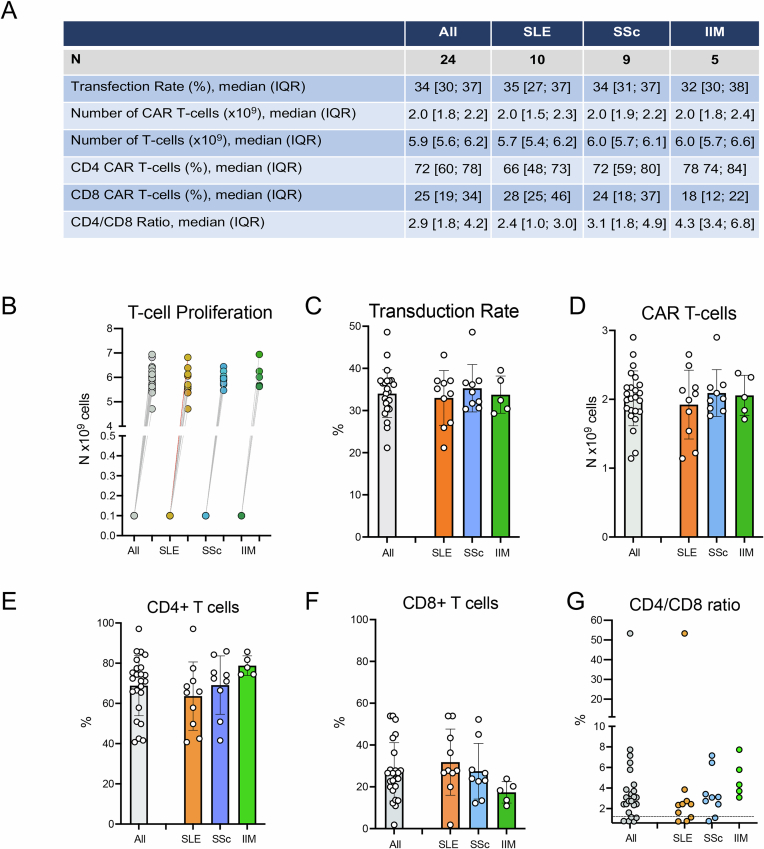
Parameters of ex vivo CAR T-cell generation. (**a**) Summary of product characteristics in the entire population (N = 24) and in patients with systemic lupus erythematosus (SLE; N = 10), systemic sclerosis (SSc, N = 9) and idiopathic inflammatory myositis (IIM, N = 5); numbers are medians and interquartile ranges; (**b**) in vitro T-cell expansion; (**c**) transduction efficacy (% CAR-expressing cells of T-cells), (**d**) number of CAR T-cell at the end of the production process, (**e**) % CD4 + T-cells of CAR T-cells, (**f**) % CD8 + T-cells of CAR T-cells, (**g**) CD4 + /CD8+ ratio in CAR T-cells.

**Extended Data Fig. 3:**
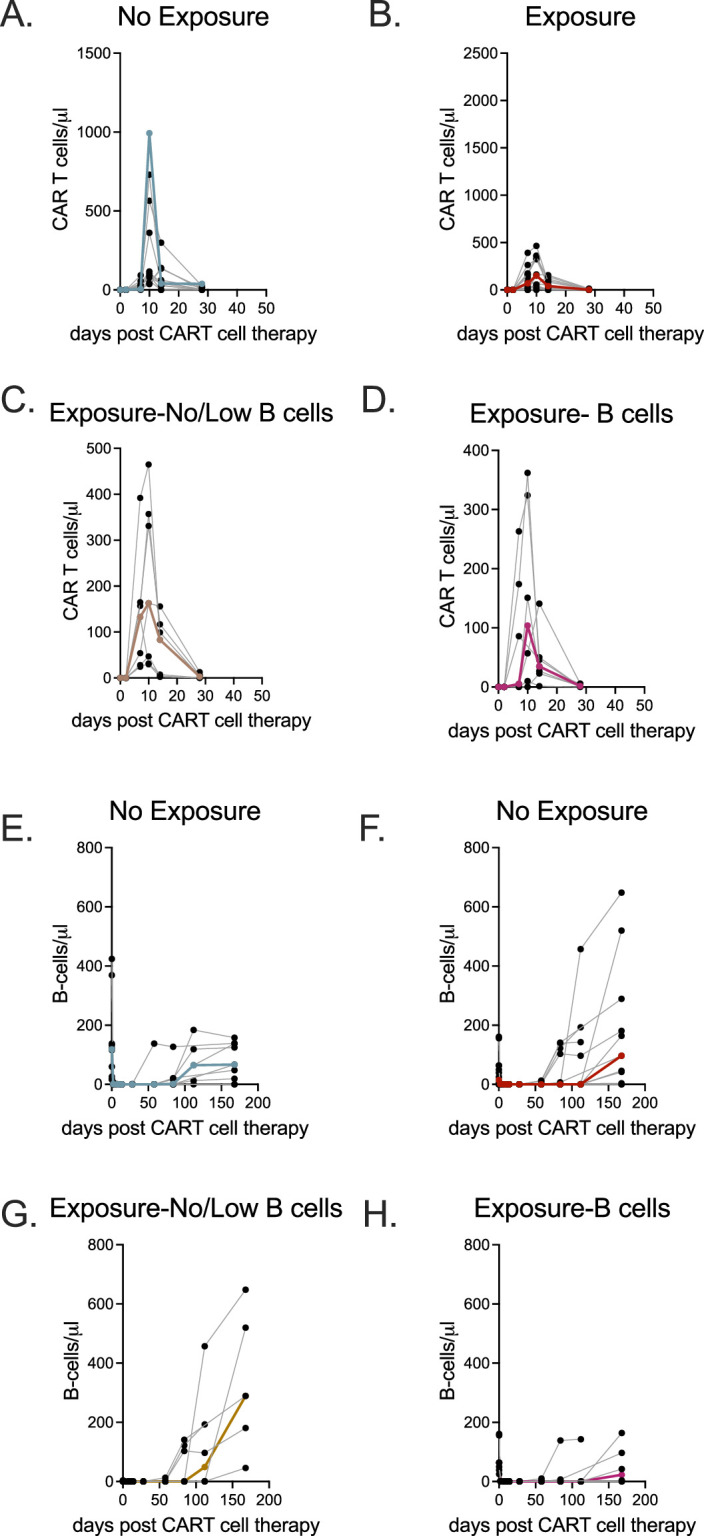
CAR T-cell expansion and B-cell reconstitution in patients with and without previous exposure to protein-based B-cell depleters. (**a-d**) Expansion and involution of chimeric antigen receptor (CAR) expressing T-cells in patients with no previous exposure to protein-based B-cell depleters (**a**; N = 10), previous exposure to protein-based B-cell depleters (**b**; N = 14) and those exposed with no or low B-cells at baseline (**c**; N = 7) or normal B-cells as baseline (**d**; N = 7). (**e-h**) Recovery of B-cells in patients with no previous exposure to protein-based B-cell depleters (**e**), previous exposure to protein-based B-cell depleters (**f**) and those exposed with no or low B-cells at baseline (**g**) or normal B-cells as baseline (**h**). Colored line shows the median.

Product characteristics of Zorpo-cel are summarized in Extended Data Fig. 2 and were notably homogeneous across SLE, IIM and SSc despite the substantial differences in T cell toxic drug exposure before leukapheresis. In brief, the median transduction efficacy was 34% (IQR 30−37) with CD4^+^ cells dominating the product over CD8^+^ T cells, leading to a CD4/CD8 ratio of 2.9 (IQR 1.8−4.2). From a fixed starting population of 0.1 × 10^9^ cells, the T cells expanded to a median of 5.9 × 10^9^ (IQR 5.6−6.2) at day 12. The median number of CAR-T cells produced was 2.0 × 10^9^ (IQR 1.8−2.2). No major differences in expansion characteristics were found between leukaphereses from patients with SLE, SSc and IIM. No out-of-specification product occurred. After administration of Zorpo-cel, CAR-T cells expanded to a peak of 140 (82−360) cells/μl after 10 (10−10) days (Table 2 and Fig. 2a). CAR-T cells were no longer detected after a median of 83 (56−113) days. Expansion of CAR-T cells was lower in patients with previous exposure to antibody-based B cell depleters (that is, rituximab), although this did not correlate with baseline B cell counts (Extended Data Fig. 3).

**Table 2:** Dynamics of CAR-T cell expansion and leukocyte changes

*N*	All	SLE	SSc	IIM
	24	10	9	5
CAR-T cell characteristics				
Maximum (cells/μl), median (IQR)	140 (82−360)	140 (75−358)	132 (69−546)	324 (48−398)
Time to maximum (days), median (IQR)	10 (10−10)	10 (10−14)	10 (10−11)	10 (9−10)
Time to <1 cell/μl (days), median (IQR)	56 (30−58)	56 (28−85)	56 (42−71)	43 (30−58)
Time to 0 cells/μl (days), median (IQR)	83 (56−113)	98 (63−154)	84 (43−99)	56 (37−58)
B cell characteristics				
Time to 0 cells/μl (days), median (IQR)	7 (0−7)	5 (0−7)	7 (1−7)	7 (0−7)
Recovery >1 cell/μl (days), median (IQR)	84 (58−140)	86 (43−140)	84 (65−147)	98 (65−154)
Leukocyte characteristics				
Nadir (cells/μl), median (IQR)	1,245 (735−1,845)	670 (149−1,060)	1,600 (1,075−1,980)	1,710 (1,265−2,745)
Time to nadir (days), median (IQR)	7 (7−7)	7 (7−7)	7 (7−9)	7 (7−9)
Patients <2,000 cells/μl (grade 3), *N* (%)	20 (83%)	10 (100%)	7 (78%)	3 (60%)
Time to >2,000 cells/μl (days), median (IQR)	14 (10−15)	14 (13−19)	10 (5−12)	14 (0−15)
Patients <1,000 cells/μl (grade 4), *N* (%)	11 (46%)	8 (80%)	11 (22%)	1 (20%)
Time to >1,000 cells/μl (days), median (IQR)	0 (0−14)	12 (8−14)	0 (0−6)	0 (0−8)
Neutrophil characteristics				
Nadir (cells/μl), median (IQR)	530 (243−891)	274 (80−521)	681 (430−939)	897 (276−1,229)
Time to nadir (days), median (IQR)	9 (7−10)	7 (7−10)	7 (7−10)	9 (7−10)
Patients <1,000 cells/μl (grade 3), *N* (%)	20 (83%)	9 (90%)	7 (78%)	4 (80%)
Time to >1,000 cells/μl (days), median (IQR)	14 (10−16)	15 (10−28)	14 (5−14)	14 (7−16)
Patients <500 cells/μl (grade 4), *N* (%)	11 (46%)	7 (70%)	2 (22%)	2 (40%)
Time to >500 cells/μl (days), median (IQR)	0 (0−14)	12 (0−14)	0 (0−7)	0 (0−15)
Lymphocyte characteristics				
Nadir (cells/μl), median (IQR)	88 (34−113)	54 (16−125)	92 (45−142)	84 (20−103)
Time to nadir (days), median (IQR)	2 (0−2)	2 (0−2)	2 (1−3)	2 (1−5)
Patients <500 cells/μl (grade 3), *N* (%)	24 (100%)	10 (100%)	9 (100%)	5 (100%)
Time to >500 cells/μl (days), median (IQR)	10 (10−29)	14 (10−110)	10 (8−13)	10 (7−23)
Patients <200 cells/μl (grade 4), *N* (%)	23 (96%)	10 (100%)	8 (89%)	5 (100%)
Time to >200 cells/μl (days), median (IQR)	7 (7−9)	7 (7−10)	7 (3−7)	7 (5−9)

### B cell depletion and effects on white blood cells

**Extended Data Fig. 4:**
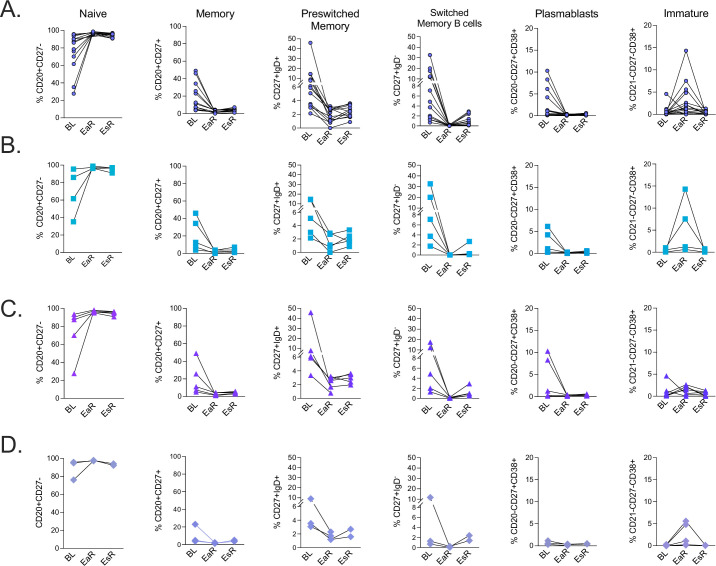
B-cell subsets before and after CAR T-cell therapy. Percentages of naïve CD20 + CD27- B-cells, CD20 + CD27+ total memory B-cells, CD27+IgD+ pre-switched memory B-cells, CD27+IgD- witched memory B-cells, CD20-CD27 + CD38+ plasmablasts and immature CD21-CD27-CD38 + B-cells in all patients (N = 23; N = 6 with only one time point) (**a**), patients with systemic lupus erythematosus (SLE) (N = 9; N = 3 with only one time point) (**b**), systemic sclerosis (SSc) (N = 9; N = 1 with only one time point) (**c**) and idiopathic inflammatory myositis (IIM) (N = 5; N = 2 with only one time point) (**d**). BL, baseline; EaR, early reconstitution, EsR, established reconstitution. Analysis was done in patients with sufficient numbers ( > 10 cells, microliter) of baseline B-cells and reconstituted B-cells.

B cells were rapidly depleted in all patients after a median time of 7 (0−7) days (Fig. 2b). At baseline, four patients had no circulating B cells, and three patients had fewer than 10 B cells/μl, which was explained by previous antibody-based B cell depletion. Median time of B cell aplasia after CAR-T cell infusion was 83 (56−113) days. Four (three SLE and one SSc) of the twenty-four patients did not reconstitute B cells within 6 months. B cell reconstitution did not differ between patients previously receiving antibody-based B cell depleters and those naive to such agents (Extended Data Fig. 3). B cell analysis in the 13 patients who had B cells at baseline and showed sufficient reconstitution of B cells at follow-up showed B cell reset after CAR-T cell therapy, with dominance of naive B cells (>90%) and virtual loss of the memory B cell and plasmablast compartments in all three diseases (Extended Data Fig. 4).

Effects on white blood cell counts, neutrophils, CD4^+^ T cells and CD8^+^ T cells are depicted in Table 2 and Fig. 2c–f. Duration of grade 3 or higher leukopenia (<2,000 cells/ml), neutropenia (<1,000 cells/ml) and lymphopenia (<500 cells/ml) was short, attributing to 14 (10−15), 14 (10−16) and 10 (10−29) days, respectively. Duration of grade 4 leukocytopenia (<1,000 cells/ml), neutropenia (<500 cells/ml) and lymphopenia (<200 cells/ml) were even shorter at 0 (0−14), 0 (0−14) and 7 (7−9) days, respectively. Grade 3 and grade 4 leukocytopenia and neutropenia occurred more frequently in patients with SLE than in patients with IIM and SSc. There was no relevant thrombocytopenia (>grade 1) in any of the patients.

### Primary endpoint and safety assessments

The study met its primary endpoint. Only one dose-limiting toxicity (DLT) event (see below) was recorded in the first phase of the study (maximal four allowed), and no predefined toxicity events were recorded in the second phase of the study (maximal six allowed). No higher-grade (grade 3 or 4) CRS occurred throughout the entire study (Table 3). Seventy percent of the patients (17 out of 24) developed CRS grade 1, and only one CRS grade 2 was recorded in a patient with SSc. Median onset of CRS was 1 (1−6) day after CAR-T cell administration and lasted a median of 3 (1−5) days. Fifty-eight percent of the patients (14 out of 24) received at least one dose of tocilizumab in the context of CRS, and dexamethasone was administered to four patients (17%). No ICANS occurred, and no clinically relevant neutropenia or thrombocytopenia persisted beyond day 28. In all 24 patients, immune effector cell-associated hematotoxicity (ICAHT) and local immune effector cell-associated toxicity (LICATS) were mild, as summarized in Table 3.

**Table 3:** Toxicity analysis

*N*	All	SLE	SSc	IIM
	24	10	9	5
CRS				
CRS (any grade), *N* (%)	18 (75%)	9 (90%)	6 (67%)	3 (60%)
CRS (grade 1), *N* (%)	17 (71%)	9 (90%)	5 (56%)	3 (60%)
CRS (grade 2), *N* (%)	1 (4%)	0 (0%)	1 (11%)	0 (0%)
CRS (grade 3), *N* (%)	0 (0%)	0 (0%)	0 (0%)	0 (0%)
CRS (grade 4), *N* (%)	0 (0%)	0 (0%)	0 (0%)	0 (0%)
Time to CRS (days), median (IQR)	1 (1−6)	1 (1−7)	1 (1−6)	2 (1−6)
Duration of CRS (days), median (IQR)	3 (1−5)	4 (2−5)	2 (1−4)	3 (2−5)
Tocilizumab, *N* (%)	14 (58%)	8 (80%)	5 (56%)	2 (40%)
Glucocorticoids, *N* (%)	4 (17%)	3 (33%)	1 (11%)	0 (0%)
ICANS				
ICANS (any grade), *N* (%)	0 (0%)	0 (0%)	0 (0%)	0 (0%)
ICAHT				
Early ICAHT (any grade), *N* (%)	14 (58%)	9 (90%)	3 (33%)	2 (40%)
Early ICAHT (grade 1), *N* (%)	4 (17%)	3 (30%)	1 (11%)	0 (0%)
Early ICAHT (grade 2), *N* (%)	9 (37%)	6 (60%)	1 (11%)	2 (40%)
Early ICAHT (grade 3), *N* (%)	1 (4%)	0 (0%)	1 (11%)	0 (0%)
Early ICAHT (grade 4), *N* (%)	0 (0%)	0 (0%)	0 (0%)	0 (0%)
Late ICAHT (any grade), *N* (%)	6 (25%)	5 (50%)	1 (11%)	0 (0%)
Late ICAHT (grade 1), *N* (%)	2 (8%)	1 (10%)	1 (11%)	0 (0%)
Late ICAHT (grade 2), *N* (%)	1 (4%)	1 (10%)	0 (0%)	0 (0%)
Late ICAHT (grade 3), *N* (%)	3 (13%)	3 (30%)	0 (0%)	0 (0%)
Late ICAHT (grade 4), *N* (%)	0 (0%)	0 (0%)	0 (0%)	0 (0%)
G-CSF administration	3 (13%)	3 (30%)	0 (0%)	0 (0%)
LICATS				
LICATS (any grade), *N* (%)	21 (88%)	10 (100%)	6 (66%)	5 (100%)
LICATS (grade 1), *N* (%)	11 (46%)	5 (50%)	3 (33%)	3 (60%)
LICATS (grade 2), *N* (%)	9 (38%)	4 (40%)	3 (33%)	2 (40%)
LICATS (grade 3), *N* (%)	1 (4%)	1 (10%)	0 (0%)	0 (0%)
LICATS (grade 4), *N* (%)	0 (0%)	0 (0%)	0 (0%)	0 (0%)

Only prednisolone and no other immunosuppressive treatment was used between cessation of immunosuppression before leukapheresis and CAR-T cell infusion (‘bridging treatment’). Fourteen (eight SLE, four SSC and two IIM) of the twenty-four patients received prednisolone as bridging treatment. The median (IQR) prednisolone dose was 7.5 (5−10) mg per day with a median duration of 8.5 (5.7−14) days. Four flares occurred between cessation of immunosuppression and CAR-T cell therapy, all in patients with SLE. Flares were treated with prednisolone with a cumulative prednisolone dose of 60 mg (rash, arthritis), 280 mg (rash, ulcers, alopecia), 490 mg (nephritis, rash, serositis) and 500 mg (nephritis, carditis), respectively. No other medications were used for flares, except mycophenolate (cumulative dose of 16 g) in the one patient with SLE in whom the flare presented as nephritis and carditis.

One event of grade 3 organ toxicity occurred in the first phase of the study in a patient with SLE. After leukapheresis and discontinuation of immunosuppression and tapering of prednisone to 5 mg per day, the patient developed a severe SLE flare with rash, polyserositis and hypertensive crisis (see above). Prednisone was increased to an initial daily dose of 100 mg, which led to stabilization. Lymphodepletion and CAR-T cell infusion could be conducted as planned. Prednisone was stopped prior to lymphodepletion. During the first week after CAR-T cell infusion, creatinine increased with acute kidney insufficiency grade 3. Systemic and pulmonary cytomegalovirus (CMV) reactivation was detected. Biopsy was performed on day 30 after CAR-T cell therapy, primarily showing microthrombus formation in the afferent arterioles and peripheral interlobular arteries resembling thrombotic microangiopathy. Some hyaline deposits (wire loops), mild interstitial inflammation and tubular damage could be identified. B cells were completely absent, and T cells were scarce in the interstitium. Immunoglobulin deposits were still found, most likely due to the short time after CAR-T cell infusion, providing not enough time to resolve them. Activity and chronicity index were rated as 4/24 and 4/12, respectively. The kidney biopsy was complicated by severe intracapsular bleeding requiring multiple transfusions and intrarenal coiling. CMV infection was successfully treated with ganciclovir. The patient recovered thereafter with stably increased renal retention parameters (serum creatinine between 2 mg dl^−1^ and 3 mg dl^−1^). No further immunosuppression was needed.

Three cases of delayed grade 3 neutropenia were recorded, which occurred at a median of 59 (29−84) days after CAR-T cell administration and resolved at a median of 7 (1−7) days after a median of four (1−6) doses of granulocyte colony-stimulating factor (G-CSF). All patients who presented with a late neutropenia were those with SLE without clinical or serological evidence of ongoing disease activity. The respective patients had no prior history of neutropenia, excluding the underlying disease as cause of neutropenia. Also, other hematopoietic cell lineages (red blood cells and platelets) were not altered. In two patients, anti-neutrophil antibodies were examined, showing negative results. Furthermore, bone marrow biopsy in two patients showed unspecific findings with only reactive changes. Delayed neutropenia fully resolved in all three cases.

**Extended Data Fig. 5:**
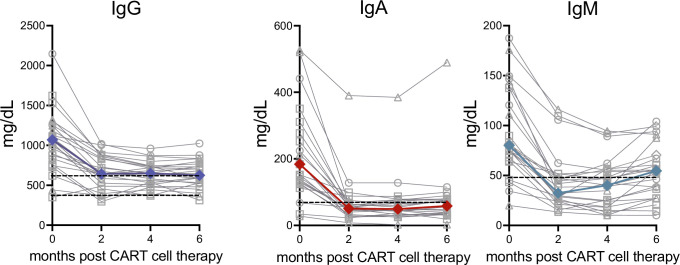
Immunoglobulin levels. Serum levels of immunoglobulin G (IgG), immunoglobulin A (IgA) and immunoglobulin M (IgM) (N = 24). Dashed lines indicate lower level of normal; the lower dashed line in the IgG graph shows the limit of immunoglobulin substitution (400 mg/dL).

Levels of immunoglobulins (IgG, IgA and IgM) decreased after CAR-T cell therapy (Extended Data Fig. 5). Seven patients (29%) required intravenous immunoglobulin substitution due to higher-grade hypogammaglobulinemia or recurrent mild infections.

The most common adverse events according to National Cancer Institute Common Terminology Criteria for Adverse Events (CTCAE) were blood and lymphatic system disorders (98 records), with 34 recorded neutropenia of any grade and 35 instances of leucopenia mainly related to lymphodepletion. Infections and infestations were recorded 60 times, mostly consisting of mild upper respiratory infections. Fifteen serious adverse events occurred, and all but one recovered without sequelae. The one serious adverse event that did not resolve (persisting grade 3 kidney function impairment) was described above. The most common adverse events of special interest were CRS and disease flares before advanced therapy medicinal product (ATMP) administration. The four flares between leukapheresis and CAR-T cell administration were exclusively in patients with SLE. They were successfully controlled with short but high-dose courses of glucocorticoids. Adverse events, serious adverse events and adverse events of special interest are summarized in Supplementary Tables 1 and 2.

### Secondary endpoint and efficacy assessment

**Fig. 3:**
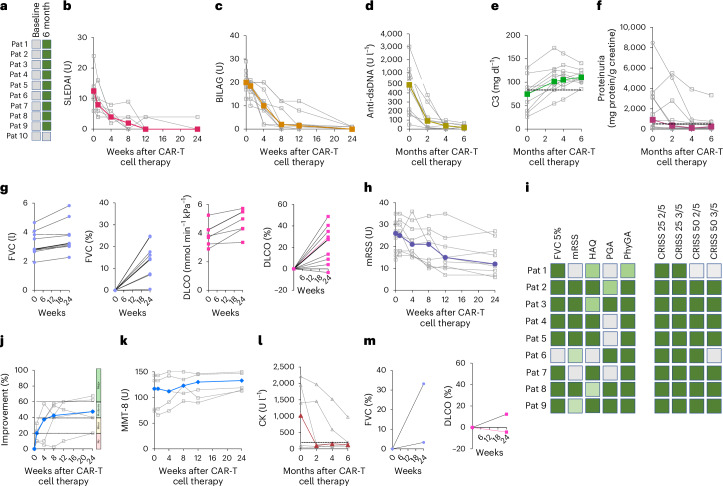
Clinical efficacy of CAR-T cell treatment. DORIS remission in patients (Pat) with SLE (*N* = 10; green boxes indicate DORIS remission) (**a**), SLEDAI (*N* = 10) (**b**), BILAG scores (*n* = 10) (**c**), levels of dsDNA antibodies (**d**), complement factor C3 (**e**) and proteinuria (**f**). **g**, Lung function shown by absolute and relative changes of FVC and DLCO in patients with SSc (*n* = 9). **h**, mRSS scores (*n* = 9). **i**, CRISS response. Left: individual components (improvement of FVC > 5%, dark green; improvement of mRSS > 25%, light green; improvement of mRSS > 50%, dark green; improvement of HAQ, patient-based global disease activity (PGA) and physician global disease activity (PhyGA) > 25%, light green, >50%, dark green). Right: 25% and 50% CRISS responses in 2/5 and 3/5 components. **j**–**l**, Total improvement score (**j**), MMT-8 (**k**) and serum creatinine kinase (CK) level (**l**) in patients with idiopathic IIM (*n* = 5). **m**, Lung function in patients with IIM with interstitial lung disease (*n* = 2). Bold colored lines in graphs show medians of individual patients (grey lines).

**Extended Data Fig. 6:**
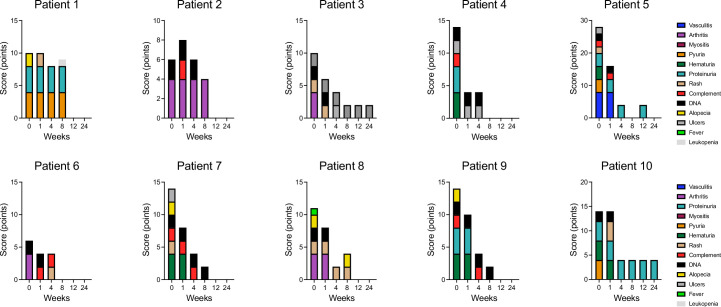
Individual SLEDAI courses. Graphs show the courses of Systemic Lupus Erythematosus Activity Indices (SLEDAI, SELENA version) in the 10 patients with SLE; DNA, anti-double stranded DNA antibodies.

**Extended Data Fig. 7:**
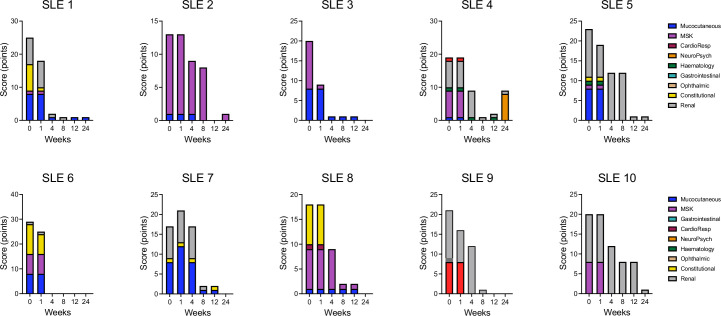
Individual BILAG courses. Graphs show the courses of British Isles Lupus Assessment (BILAG) scores in the 10 patients with systemic lupus erythematosus (SLE); 12 points is corresponding BILAG-A (very severe disease activity requiring immunosuppressive treatment and high doses of glucocorticoids), 8 points is corresponding BILAG-B (moderate disease activity requiring hydroxychloroquine treatment and lower doses of glucocorticoids), 1 point is corresponding BILAG-C (low disease activity requiring non-steroidal ant-inflammatory drugs or analgesics) and 0 points is corresponding BILAG-D and BILAG-E (no current disease activity or never involved); MSK, musculoskeletal involvement.

Efficacy of CD19 CAR-T cell therapy was assessed after 6 months, requiring the fulfilment of DORIS remission in SLE, ACR moderate/major response in IIM and no progression of lung disease in SSc. To continue with phase 2a, at least four patients had to reach these efficacy endpoints in phase 1. The clinical efficacy endpoint was met in all eight patients (five SLE, two SSc, one IIM) in phase 1. In phase 2a, 14 of the 16 patients met the clinical efficacy endpoint (overall response rate of 87.5%, 90% confidence interval 65.6−97.7). One patient with IIM did not adequately improve in muscle strength, probably due to longstanding disease and atrophy and, therefore, did not meet ACR moderate/major response criteria. One patient with SLE did not achieve significant reduction of proteinuria (<500 mg protein/g creatinine) and, therefore, did not meet DORIS remission at 6 months (Fig. 3a). However, this patient with SLE remained drug free, and the SLEDAI decreased from 14 to 4 and the British Isles Lupus Assessment Group (BILAG) decreased from 20 to 1. Despite being lower than baseline, increased proteinuria of two additional patients SLE persisted at month 6 (669 mg protein/g creatinine and 764 mg protein/g creatinine). However, kidney biopsies of both patients showed no signs of active lupus nephritis (one patient with thrombotic microangiopathy as described above and one patient with a chronicity index of 4/12 and an activity index of 0/24). Both patients remained drug free within 18 months of follow-up. The steering committee of the study, therefore, assigned these patients to DORIS remission (Fig. 3a). The SLEDAI score significantly decreased from a median of 12.5 (9.0−14.0) units to 0 units (Fig. 3b and Supplementary Table 3). Furthermore, all BILAG A and BILAG B activity at baseline disappeared at 6 months of follow-up (Fig. 3c). Individual SLEDAI and BILAG courses are depicted in Extended Data Figs. 6 and 7. All patients with SLE showed positive anti-double-stranded DNA (dsDNA) antibodies at baseline, which seroconverted to a seronegative state within 6 months (Fig. 3d). Complement factor C3 was low in 7 out of 10 patients with SLE and normalized in all of them at follow-up (Fig. 3e). Proteinuria was present in 7 out of 10 patients with SLE and decreased in 6 out of 7 patients compared to baseline, with 4 patients reaching levels below 500 mg protein/g creatinine. There was no new-onset proteinuria and no worsening of proteinuria despite cessation of all immunosuppression (Fig. 3f).

In the nine patients with SSc, no worsening of lung function was noted. Instead, forced vital capacity (FVC) significantly increased from a median of 2.83 l to 3.23 l (+14.1%), and DLCO significantly increased from a median of 4.23 mmol ml^−^^1^ kPa^−^^1^ to 5.48 mmol ml^−1^ kPa^−^^1^ (+29.5%) (Fig. 3g and Supplementary Table 3). mRSS scores significantly decreased from a median of 26 to 12 units (Fig. 3h and Supplementary Table 3). The ACR CRISS (Composite Response Index in Systemic Sclerosis) 25 response was fulfilled in all patients, CRISS 50 (2/5 items) in eight of nine patients and CRISS 50 (3/5 items) in seven of nine patients (Fig. 2i). In IIM, four out of five patients reached ACR moderate/major response, with MMT-8 scores increasing from a median of 117 to 133 units (Fig. 3j,k). Creatine kinase levels were increased in three of five patients with IIM prior to therapy, with normalization observed in two out of three patients and a marked decrease in one patient 6 months after CD19 CAR-T cell infusion (Fig. 3l and Supplementary Table 3). Lung function in patients with IIM with interstitial lung disease remained stable (Fig. 3m).

**Extended Data Fig. 8:**
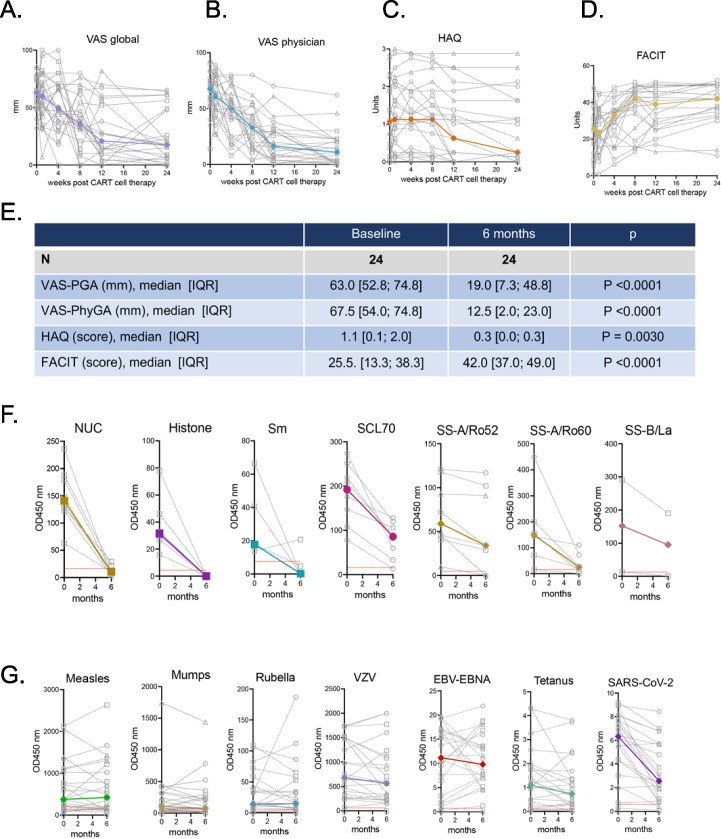
Efficacy of CAR T-cell treatment on patients-related outcomes, autoantibody and vaccination-related antibody levels. Effects of treatment with chimeric antigen receptor (CAR) T-cells on (**a**) patient-based global disease activity measured by visual analogue scale (VAS; N = 24), (**b**) physician-based global disease activity measured by visual analogue scale (VAS; N = 24), (**c**) physical function measured by health assessment questionnaire (HAQ; N = 24) and (**d**) fatigue measured by Functional Assessment of Chronic Illness Therapy - Fatigue (FACIT; N = 24); (**e**) Changes in patient-related outcomes between baseline and 24-week follow-up. Visual Analogue Scale (VAS) for Patient-rated Global Disease Activity (PGA) and Physician-rated Global Disease Activity (PGA); Health Assessment Questionaire (HAQ); Functional Assessment of Chronic Illness Therapy – Fatigue Scale (FACIT). P-values are based on Wilcoxon signed rank test. (**f**) Effects on autoantibodies against nucleosomes (N = 8; in SLE), histones (N = 6; in SLE), SCL70 (N = 8; in SSc), SS-A/Ro60 (N = 6, in all), SS-A/Ro52 (N = 8; in all). (**g**) Effects on vaccination-related antibodies against measles, mumps, rubella, varicella zoster virus (VZV), Epstein Barr Virus (EBV), tetanus, and severe acute respiratory distress syndrome coronavirus- 2 (SARS-CoV-2) (N = 24).

With respect to patient-related outcomes, CD19 CAR-T cell treatment induced a significantly improved global health state as evaluated by both patients and physicians (Extended Data Fig. 8a,b). Furthermore, functional outcome was greatly enhanced (Extended Data Fig. 8c) and disease-associated fatigue significantly reduced (Extended Data Fig. 8d) in patients of all three diseases (Extended Data Fig. 8e).

### Autoantibodies and vaccination responses

**Extended Data Fig. 9:**
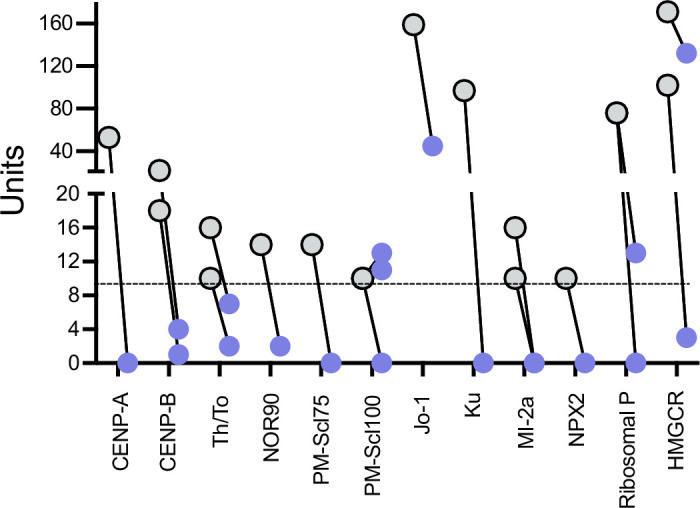
Effects of CAR T-cell therapy on additional autoantibody responses. Graphs show baseline (grey circles) and 6-months follow-up (blue circles) of autoantibody levels against various autoantigens in individual patients. CENP, centromere protein; HMGCR, 3-hydroxy-3-methylglutaryl-coenzyme A reductase; NOR90, nucleolus organizing regions 90; NPX2, nuclear matrix protein 2; PM-SCL, polymyositis-systemic sclerosis.

In addition to anti-dsDNA antibodies shown in Fig. 3d, other SLE-related autoantibodies also decreased. In patients with SLE, the levels of autoantibodies against nucleosomes, histones and Smith antigen consistently decreased and became negative in most patients (Extended Data Fig. 8f). In SSc, autoantibodies against Scl70 decreased by 66% after 6 months. Moderate decreases, but no seroconversion, were observed for anti-SS-A/Ro52, anti-SS-A/Ro60 and anti-SS-B/La antibodies, suggesting their dominant production by CD19^−^ plasma cells. Further autoantibody reactivities and their changes after CD19 CAR-T cell therapy are depicted in Extended Data Fig. 9. Antibody titers to previous vaccinations remained stable, showing no decreases in IgG responses against measles, mumps, rubella, varicella zoster virus, Epstein−Barr virus and tetanus. A moderate decrease was observed for titers of IgG antibodies against SARS-CoV-2 (Extended Data Fig. 8g). Stability of vaccination antibodies pertained to patients both receiving or not receiving intravenous immunoglobulin substitution (Supplementary Fig. 1). Details on anti-tetanus antibody responses and timing and doses of intravenous immunoglobulin substitution are shown in Supplementary Fig. 2.

### Follow-up

**Extended Data Fig. 10:**
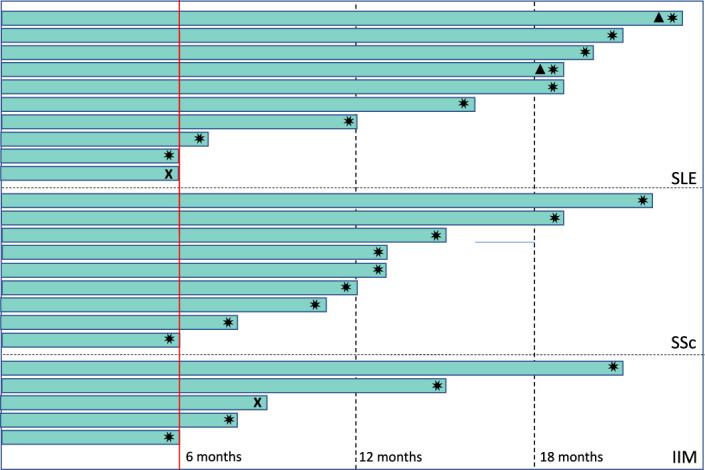
Duration of drug-free remission/no progression state. Bars show the time from CAR T-cell infusion to last follow up in each patient of the CASTLE study. Green colour indicates immunosuppression and glucocorticoid- free state. Stars indicate DORIS remission in systemic lupus erythematosus (SLE), 2016 ACR/EULAR Moderate or Major Response in idiopathic inflammatory myositis (IIM) and no progression of interstitial lung disease in systemic sclerosis (SSc). Triangles indicate kidney damage manifesting as proteinuria (SLE patient #1) and increased retention parameters (SLE patient #4). X indicates that one SLE patient (#10) has not reached DORIS remission due to proteinuria at 6 months and one IIM patient (#3) had not fulfilled 2016 ACR/EULAR Moderate or Major Response criteria due to muscular weakness (myopathy).

Within a median follow-up of 13 (8−19) months, no relapses have occurred among the 24 CASTLE patients. Swimmer plots in Extended Data Fig. 10 show individual follow-up times attributing to a total of 327 patient-months without therapy and a maximal follow-up time of 23 months. None of the patients showed signs of disease progression or relapse. Furthermore, no patient required reintroduction of immunosuppressants or glucocorticoids. This observation also pertains to the two patients (one SLE and one IIM) who did not achieve the 6-month efficacy endpoint; notably, disease activity of the SLE patient decreased from a SLEDAI of 14 to 4 and a BILAG of 20 to 1 after 6 months with low levels of circulating CAR-T cells still present, suggesting that full response may not yet have been achieved. Additionally, the IIM patient (9-month follow-up) improved in overall disease state but did not sufficiently recover in muscle strength, most likely due to long disease duration (16 years) and disease-induced and previous treatment-induced muscle wasting. Taken together, clinical responses and drug-free state were maintained in the CASTLE study patients over a median follow-up period of 1 year.

## Discussion

CASTLE is a phase 1/2a basket study that aimed to investigate the feasibility, safety and efficacy of autologous CD19 CAR-T cell therapy in patients with refractory and progressive SLE, SSc and IIM. The study showed favorable safety and good efficacy of CD19 CAR-T cell therapy with Zorpo-cel in these three pathophysiology-related autoimmune diseases. In the study, no higher-grade CRS, no ICANS and no relevant hematotoxicity were observed. Absence of higher-grade toxicities is a critical factor for the feasibility of CD19 CAR-T cell therapy in autoimmune disease and, ultimately, may facilitate its future outpatient use.

CASTLE consisted of a patient population with highly active, severe and treatment-refractory autoimmune diseases that required rapid control of their disease processes. The study population was relatively young (median age of 39 years), and the disease duration was fairly short (4 years) despite a history of a median of four ineffective prior immunosuppressive treatment regimens during this time, reflecting aggressiveness of the disease and urgent need for effective therapy. Although such patients with severe and progressive disease constitute only a fraction of patients with SLE, SSc and IIM, they may benefit the most from deep immune interventions, such as CD19 CAR-T cell therapy, before irreversible organ damage has occurred. Most notably, a stable immunosuppression-free and glucocorticoid-free state was achieved in all 24 patients not only up to but also beyond the 6-month endpoint, documenting a median of 13 months and a maximum of 23 months of follow-up. None of the patients progressed or flared during this observation time. Treatment responses were profound throughout all three indications, with DORIS remission achieved in most patients with SLE, CRISS 50 in all patients with SSc and ACR major/moderate responses in most patients with IIM. Organ damage before CAR-T cell therapy—for example, due to uncontrolled inflammation or additional events—has to be considered when interpreting outcomes; hence, we observed residual proteinuria despite biopsy-proven absence of SLE activity in two patients and persisting muscular weakness likely due to longstanding disease in a patient with IIM. Differentiation between residual activity and damage or other causes of pathology seems critical to appropriately tailor the therapeutic path in these situations. We consider biopsies of the affected tissues as a helpful approach to possible differential diagnosis and subsequent patient management.

The CASTLE study also confirmed earlier observations that CD19 CAR-T cell therapy does not erase the vaccination status, leaving preexisting vaccination-based IgG responses largely intact while eliminating most, but not all, of the autoantibodies (for example, immune responses against dsDNA, histones and nucleosomes as well as against Smith, CENP-B, Ku and Mi-2 antigens). Some autoantibodies strongly decreased (for example, Scl-70 by 66% after 6 months) but did not yet seroconvert, leaving the chance for further decrease after longer follow-up as previously demonstrated^16^. Some autoantibodies only moderately decreased after therapy, suggesting their, at least partial, plasma cell dependence, as plasma cells do usually not express CD19 and, therefore, escape CD19 CAR-T-cell-mediated cytotoxicity. Notably, there is currently no evidence that the persistence of, for example, anti-SS-A/Ro antibodies poses patients at higher risk for relapse. In fact, the pathogenicity of some of the autoantibodies, related to SLE, SSc and IIM, is not proven, whereas other functions of B cells, such as antigen presentation, should be considered as playing a crucial role in triggering autoimmune disease. Therefore, patients who do not seroconvert should not be preemptively reexposed to immunosuppressive drugs; rather, measurement of clinical disease activity should guide such decisions.

The overall safety profile of Zorpo-cel, also compared to previous experience in malignant disease, was favorable^17^. Serious adverse events were mostly defined by prolonged hospitalization. None of the serious adverse events were life-threatening, and 14 out of 15 resolved without sequelae. All CRS was mild, and no ICANS and no relevant hematotoxicity were observed. LICATS was observed in the majority of the patients, but the events were mild. The most common adverse events were reversible neutropenia and leukocytopenia (related to lymphodepletion) as well as mild infections and nausea. One DLT event (kidney injury) occurred. This event started with an SLE flare after cessation of immunosuppression necessitating high-dose glucocorticoids treatment, which facilitated CMV reactivation and was accompanied by thrombotic microangiopathy. We considered CMV reactivation as the most likely cause of thrombotic microangiopathy in the kidneys^18^, although two cases of thrombotic microangiopathy after CAR-T cell treatment have been reported in B cell malignancies independent of CMV reactivation/infection. Their course (2 months after CAR-T cell infusion) and clinical presentation (thrombocytopenia), however, did not match our case^19^. Although the patient recovered with no further signs of SLE activity, she suffered from kidney damage due to a combination of thrombotic microangiopathy, hypertensive crisis and acute SLE flare. The event led to adaptation of patient management with the goal of best possible disease control before CAR-T cell infusion. With this approach, no further toxicities occurred. Generally, a shorter pause of immunosuppressive therapy should be considered in autologous CAR-T cell therapy to avoid flares. This can be realized by shorter manufacturing times as point-of-care production. In addition, as there is currently no evidence that immunosuppression generally impairs the manufacturing of CAR-T cells, stopping such treatments before and around leukapheresis seems not necessarily required.

Reversible delayed neutropenia (>28 days after CAR-T cell infusion) was observed in three patients, all of whom had a diagnosis of SLE. Bone marrow biopsy was inapparent, and no anti-neutrophil autoantibodies were found. The pathogenesis of delayed neutropenia is not fully clear, but it may represent a bone marrow manifestation of LICATS or a transient alteration of the stem cell niche in the bone marrow. Thus, stromal cell-derived factor 1 (SDF-1) levels have shown to be related to the presence of delayed neutropenia^20^. SDF-1 regulates the development of hematopoietic stem cells, neutrophils and very early B cells in the bone marrow. The initial recovery of early B cells in the bone marrow may compete for SDF-1 levels and, therefore, impair neutrophil development and migration. Another safety issue could theoretically arise from delayed reconstitution of B cells. Thus, four of the twenty-four patients did not reconstitute B cells after 6 months; however, among these four, two reconstituted shortly thereafter. Although we did not find a particular safety signal in these patients, prolonged B cell depletion may promote a higher risk for infection.

A strength of this study is its phase 1/2a basket trial design according to a Bryant and Day two-stage optimal framework and formal power calculation, which is unprecedented in studying CAR-T cell treatment of autoimmune diseases. The Bryant and Day design rejects the new agent if either the response rate is inadequate or the toxicity is excessive. The strength of such two-stage designs for binary or composite endpoints is to include subgroups while maintaining competitive statistical performances and preventing the inflation of sample size^21–23^. Rubinstein^24^ explicitly mentioned the Bryant and Day design as an innovative phase 2 design in his landmark review paper on the evolution of phase 2 trials. Although CASTLE enrolled a rather small number of patients, the trial size is similar to that of other studies for treatment-refractory autoimmune diseases. Notably, pivotal phase 2 studies for CAR-T cells in autoimmune diseases are rather ‘small’ single-arm studies as well, as these studies are designed to test the likelihood that a CAR-T cell product induces drug-free remission or non-progression of a treatment-refractory autoimmune disease against the likelihood that the patient will spontaneously achieve such drug-free remission or non-progression, which is very unlikely. Therefore, the sample size of such studies is rather small—for example, 25 patients for the pivotal study for CD19 CAR-T cells in the treatment of stiff-person syndrome^25^. Another key strength is its convincing risk−benefit ratio, with only one infection-associated DLT event but otherwise minimal CAR-T-cell-related toxicities combined with robust clinical efficacy. Distinguishing CASTLE from other ongoing trials on CAR-T cells in autoimmune diseases, the fresh-in/fresh-out point-of-care product may have contributed to the favorable risk−benefit profile.

The lack of a control arm is a limitation of the study. Although such an approach would be optimal from a causal inference standpoint, it is hardly feasible in an early development setting, as a placebo arm would have been ethically challenging. Also, a best practice comparator arm would have been challenging because patients already experienced the failure of multiple effective treatment options, half of them being treated with protein-based B cell depleters. Furthermore, the profound overall treatment response with drug-free remissions observed in our study is extremely unlikely to be a spontaneous occurrence given the potentially fatal natural course of severe systemic autoimmune diseases, attesting to the efficacy of CAR-T cell treatment. This concept is also endorsed by upcoming pivotal studies of CAR-T cell treatment in autoimmune diseases. Another limitation is that bridging and flare therapy before CAR-T cell treatment may have influenced results. However, this seems unlikely as bridging therapy was based on an average of 7.5 mg of prednisolone per day, which was gradually tapered and stopped with the CAR-T cell infusion, and flare treatment with glucocorticoids was necessary in only four patients (one of them also receiving an additional short course of mycophenolate)—agents that were previously used in these patients with no sufficient control of the disease and that were also stopped with CAR-T cell infusion. Nonetheless, these data provided valuable information that immunosuppression might need to be maintained longer, especially during leukapheresis and CAR-T cell production, to shorten the drug-free interval and de-risk flares. Finally, the use of urine protein-to-creatinine ratio instead of 24-h urine protein quantification, a standard approach for measuring proteinuria, is a limitation of the study. However, it has been shown that these two parameters are well correlated with each other, making substantial deviations unlikely^26^.

In summary, the CASTLE study showed safety and preliminary efficacy of Zorpo-cel in patients with SLE, SSc and IIM. The once-in-a-time treatment approach leading to sustained drug-free remission is particularly attractive and unprecedented in autoimmune disease. T cell engagers targeting B cells^27,28^ and plasma cells^28,29^ have shown efficacy in treatment-refractory autoimmune diseases. So far, it is unclear whether such agents can indeed reset the immune system, similar to CAR-T cells, and allow the achievement of sustained drug-free remission. Innovative trial designs are needed to answer this question. CASTLE adds substantial new insights into CD19 CAR-T cell therapy of autoimmune disease and will help to design the pivotal study on Zorpo-cel in autoimmune diseases.

## Methods

### Patients

CASTLE (NCT06347718; EudraCT: 2022-001366-35; EUCT: 2024-516819-24-00 after transfer of the EU clinical trial registry system; Deutsches Register Klinischer Studien DRKS00032279) is a basket study that recruited patients with active, severe and treatment-resistant SLE, SSc and IIM. The protocol of the CASTLE study was amended in January 2024 and in June 2025, the latter due to sponsor change. The study protocol of the CASTLE study can be found in the supplementary material. Regarding eligibility, patients with SLE had to (1) fulfill the 2019 ACR/European Alliance of Associations for Rheumatology (EULAR) classification criteria of SLE^30^; (2) show active disease, defined as at least one organ system with a BILAG A score (severe disease activity) or two or more organ systems with a BILAG B score (moderate disease activity); (3) show positivity for anti-dsDNA, anti-histone, anti-nucleosome or anti-Sm autoantibodies; and (4) exhibit insufficient response or intolerance to at least two immunomodulatory drugs. Patients with SSc had to (1) fulfill the 2013 ACR/EULAR classification criteria of SSc^31^; (2) show positivity for at least one SSc-specific autoantibody; (3) exhibit signs for fast disease progression with mRSS of 10−35 units at screening and maximal disease duration of 7 years, increased acute phase reactants and progression mRSS of ≥3 units over 6 months; and (4) exhibit insufficient response or intolerance to at least two immunomodulatory or anti-fibrotic drugs. Patients with IIM had to (1) fulfill the 2017 ACR/EULAR classification criteria for probable or definite IIM^32^; (2) show active myositis in muscle biopsy or muscle magnetic resonance imaging and/or signs of interstitial lung disease related to IIM; (3) show positivity for at least one myositis-specific autoantibody; and (4) exhibit insufficient response or intolerance to at least two immunomodulatory drugs. Patients younger than 18 years and those with severely impaired renal (estimated glomerular filtration rate (eGFR) ≤ 30 ml min^−1^ m^−^^2^), liver (Child Pugh C), heart (New York Heart Association (NYHA) IV and ejection fraction ≤ 30%) or pulmonary (FVC and DLCO < 30%) function were excluded from the study. Also, patients with the diagnosis of a malignant disease in the last 5 years before screening and those with concomitant severe infections were excluded from the study. All participants provided written informed consent in compliance with Declaration of Helsinki principles.

### CAR-T cell manufacturing and treatment

Zorpo-cel consists of autologous anti-CD19 CAR-T cells, derived from a CD4/CD8-enriched leukapheresis product. Immunosuppressive drugs were stopped 21 days before leukapheresis, and glucocorticoids were tapered to a maximum of 10 mg per day. Leukapheresis was performed in each individual patient at day −13 for CAR-T cell generation with an anti-CD19 CAR-expressing pLTG1563-based lentiviral vector encoding for a second-generation CAR with an FMC63 single-chain variable fragment (scFv; anti-CD19 binding domain), 4-1BB co-stimulatory domain and CD3ζ stimulatory domain. CAR-T cells were manufactured using the CliniMACS Prodigy device (Miltenyi Biotec). Before the infusion of 1 × 10^6^ CAR-T cells per kilogram of body weight, all patients received lymphodepleting chemotherapy with fludarabine (25 mg m^−^^2^ per day intravenously on days −5 to −3) and cyclophosphamide (1,000 mg m^−^^2^ intravenously on day −3). Further details on CD19 CAR-T cell manufacturing and the treatment procedure are outlined in the appendix (pages 17−18).

### Study objectives and endpoints

The primary objective of the CASTLE study was to assess the safety and tolerability of Zorpo-cel in patients with SLE, SSc and IIM. Secondary objectives were (1) to assess the clinical efficacy of Zorpo-cel and (2) to investigate the duration of B cell depletion and CAR-T cell persistence. The primary endpoint was the incidence and severity grading (grades 0−4) of CRS and of ICANS within the first 4 weeks after ATMP administration. The secondary endpoint was the overall response rate at week 24 measured by disease activity composite indexes, each of them validated for the specific disease: DORIS remission in SLE^33^, 2016 ACR/EULAR moderate/major response in IIM^34^ and no progression of interstitial lung disease with worsening of FVC of more than 10% or worsening of FVC of 5−10% plus increase in respiratory symptoms or worsening of FVC of 5−10% plus progression of high-resolution computed tomography changes in SSc. Additional secondary endpoints were the duration of B cell depletion and persistence of CAR-T cells. No sex-specific analysis was carried out due to the rather small number of male patients in the study and the different diseases studied as part of the basket format.

### Safety assessments

Patients were screened daily for CRS and ICANS during the first 4 weeks after CAR-T cell infusion. CRS and ICANS were assessed according to American Society for Transplantation and Cellular Therapy consensus criteria^35^. CRS and ICANS higher than grade 2 were considered dose-limiting observations. This was also applicable for grade 3 or higher organ toxicity not preexisting or related to underlying disease occurring within 30 days after CAR-T cell administration. Myelotoxicity was defined as a DLT event if grade 3 or 4 neutropenia or thrombocytopenia for more than 28 days after CAR-T cell therapy occurred. In addition to the per-protocol analyses, bone marrow suppression after lymphodepleting therapy was assessed according to the categories of early ICAHT^36^ defined as follows: grade 1 (neutrophils ≤500/μl less than 7 days); grade 2 (neutrophils ≤500/μl less than 14 days); grade 3 (neutrophils ≤500/μl less than 30 days or ≤100/μl for 7−13 days); grade 4 (neutrophils ≤500/μl for ≥30 days or ≤100/μl for ≥14 days). Late ICAHT was defined as neutropenia after day 30 with ≤1,500/μl cells (grade 1), ≤1,000/μl cells (grade 2), ≤500/μl cells (grade 3) and ≤100/μl (grade 4) measured at two timepoints. Furthermore, due to recent insights^37^, LICATS was assessed according to the following grades: grade 1, spontaneous resolution; grade 2, glucocorticoid use; grade 3, glucocorticoid use plus prolonged hospitalization; grade 4, intensive care treatment. In addition, patients were monitored for infections over the entire 6-month study period. Adverse events, serious adverse events and adverse events of special interest were graded according to CTCAE version 5. DLT was defined as CRS higher than grade 2 or ICANS higher than grade 2 or any organ toxicity of at least grade 3, which was not preexisting and not due to the underlying disease and occurred within 30 days of cell infusion and did not resolve to grade 2 within 7 days, or at least grade 3 clinically relevant neutropenia or thrombocytopenia lasting at least 28 days from the time of infusion. The study would have been stopped if more than four of eight patients in phase 1 or more than six of sixteen patients in phase 2a had experienced DLT events.

### Efficacy assessments

Key efficacy parameters were DORIS remission in SLE, 2016 ACR/EULAR moderate/major response in IIM and no progression of interstitial lung disease in SSc. In addition, in patients with SLE, the SLEDAI and BILAG responses were assessed^38^; in patients with IIM, the MMT-8 score^39^ was used; and in patients with SSc, the mRSS^40^ along with CRISS 25 and CRISS 50 responses^41^ were used. Inflammatory serum markers, including the levels of C-reactive protein (CRP), interleukin-6 (IL-6) and ferritin were assessed in all patients. In addition, the serum levels of IgG, IgA and IgM were measured. In patients with SLE, serum levels of complement factor C3 and urine protein excretion were recorded, whereas, in patients with IIM, the serum levels of creatine kinase were recorded. With respect to patient-related outcomes, Patient’s and Physician’s Global Assessments based on a visual analog scale (VAS; 0−100 mm), Health Assessment Questionnaire–Disability Index (HAQ-DI) and Functional Assessment of Chronic Illness Therapy (FACIT)−Fatigue were recorded. Methods for quantification of CAR-T cells and leucocyte subsets, autoantibodies and vaccination-related antibodies as well as B cell analyses are described in the Supplementary Information.

### CAR T-cell production

The investigational medicinal product, MB-CART19.1, consisted of autologous CD19 CAR-transduced CD4/CD8 enriched T cells, derived from a leukapheresis product and processed using the CliniMACS Prodigy device (Miltenyi Biotec). Leukapheresis products were processed on day −13 without prior cryopreservation within their shelf life by enrichment of CD4^+^ and CD8^+^CD3^+^ T cells. The cell count used in enrichment was limited to an upper level of 1.5 × 10^9^ cells. On day −12, cells were cultured in TexMACS media supplemented with IL-7 and IL-15 (Miltenyi Biotec) and human AB serum (ZKT). All materials used were fully Good Manufacturing Pactice (GMP) compliant. For lentiviral transduction, a total cell count of 1 × 10^8^ cells was used as starting material. T cells were activated for transduction with polymeric nanomatrix conjugated to humanized CD3 and CD28 (Miltenyi Biotec, T Cell TransAct). Cells were transduced with a self-inactivating lentiviral vector expressing a CAR directed against human CD19. The second-generation lentiviral vector was kindly provided by Miltenyi Biotec. The vector encodes for an scFv, derived from the murine anti-human CD19 antibody FMC63, that binds to exon 4 of human CD19. Furthermore, it contains the information for a CD8-derived hinge region, a TNFRSF19-derived transmembrane domain, a CD3ζ intracellular domain and a 4-1BB co-stimulatory domain. Cells were expanded for 12 days under cleanroom conditions at the certified GMP laboratory of the Universitätsklinikum Erlangen (Department of Medicine 5, Hematology and Oncology) using the CliniMACS Prodigy system (Miltenyi Biotec) that performs all manufacturing steps in a single automated and functionally closed system. Final release tests and in-process controls included cellular composition, transduction rate, viability, microbiological control and endotoxin and mycoplasma testing according to *European Pharmacopeia*. MB-CART19.1 was produced for each patient individually (personalized therapy).

### CAR-T cell treatment procedure

Before leukapheresis, T cell targeted therapy was stopped for 3 weeks, and prednisolone dose was reduced to less than 10 mg per day. Patients received lymphodepleting chemotherapy with fludarabine 25 mg m^−2^ per day intravenously on days −5, −4 and −3 and cyclophosphamide 1,000 mg m^−2^ per day intravenously on day −3 before CAR-T cell transfer. On day 0, all patients received the investigational medicinal product MB-CART19.1, consisting of autologous CD19 CAR-transduced T cells at a dose of 1 × 10^6^ CAR-T cells per kilogram of body weight. CAR-T cells were administered as a short infusion (at day 0) after prophylactic application of antihistamines and acetaminophen. After CD19 CAR-T cell therapy, all patients received oral prophylaxis with acyclovir (800 mg daily for 12 months) and cotrimoxazol (960 mg three times weekly until a CD4 T cell count >200/μl was reached). Patients were monitored every day for signs of CRS and ICANS over a period of 10 days.

### Monitoring of CAR-T cells and leucocyte subsets

Absolute cell counts were determined with BD Trucount tubes (BD Biosciences) according to the manufacturer’s instructions. The following antihuman antibodies were used for flow cytometry for monitoring leukocytes and CAR-T cells after treatment: anti-CD3 (clone SK7), anti-CD4 (clone SK3), anti-CD8 (clone SK1), anti-CD14 (clone MφP9), anti-CD19 (clone SJ25C1), anti-CD45 (clone 2D1) and anti-CD56 (clone NCAM16.2) (all BD Biosciences) and CD19 CAR Detection Reagent (clone REA746) (Miltenyi Biotec). Data were acquired on an LSR Fortessa (BD Biosciences) and analyzed by FlowJo version 10 software (Tree Star). All measurements were taken from distinct samples. The gating strategy was as follows: time parameter was used to monitor instrument stability; doublets were excluded by forward scatter height (FSC-H)/forward scatter area (FSC-A); CD45^+^ events were gated; lymphocytes were determined by FSC-A/side scatter area (SSC-A); and viable T cells were gated by CD3^+^ and further subdivided in CAR^+^ and CAR^−^ T cells.

### Quantification of autoantibodies against nuclear antigens

Antibodies against several nuclear antigens were assessed by commercial ELISAs (Orgentec), including those against dsDNA (ORG 604; cutoff: 20 U ml^−1^), nucleosomes (ORG 528; 20 U ml^−1^), Sm (ORG 510; 20 U ml^−1^) and SS-A/Ro52 (ORG 652; 20 U ml^−1^). Autoantibody profiles for IIM and SSc were measured by immunoblots from Euroimmun. All measurements were taken from distinct samples.

### Quantification of vaccination responses

IgG antibodies against measles (cutoff 150 mIU ml^−1^), mumps (cutoff 70 U ml^−1^), varicella zoster virus (cutoff 70 U ml^−1^) and Epstein−Barr virus IgG (cutoff 70 U ml^−1^) were analyzed by ELISAs from Virion/Serion. IgG antibodies against rubella (cutoff 5 IU ml^−1^) were analyzed by Bioline ELISA from Abbott; IgG responses against tetanus (cutoff 0.15 U ml^−1^) were assessed by ELISA from VaccZyme (The Binding Site); and antibodies against SARS-CoV-2 (cutoff 0.8 optical density at 450 nm) were analyzed by Euroimmun. All measurements were taken from distinct samples.

### Characterization of B cells

To investigate the B cell compartment, isolated peripheral blood mononuclear cells (PBMCs) were stained with the following surface marker antibodies at baseline, after short-term reconstitution (3−6 months) and after long-term follow-up (12−24 months). The following antibodies were used: CD3-SparkBlue 550 (BioLegend, SK7), CD19-BV421 (BioLegend, HIB19), CD20-AF700 (BioLegend, 2H7), CD27–PE-Cy7 (BioLegend, M-T271) and CD38–PerCP-Cy5.5 (BD Biosciences, HIT2). Additionally, CD21-PE (BioLegend, Bu32) and IgD-BV785 (BioLegend, IA6-2) were used for baseline and short-term reconstitution timepoints and CD21-PE (BioLegend, Bu32) and IgD-PE (BioLegend, IA6-2) for long-term follow-up samples. Zombie NIR (BioLegend) and Fixable Viability Stain 780 (Thermo Fisher Scientific) were used as viability dye, respectively. PBMCs were acquired on a Cytek Northern Lights spectral analyzer and analyzed using FlowJo. B cells were gated on live CD45^+^CD3^−^CD19^+^ cells. Subpopulations of B cells were gated as CD20^+^CD38^+^CD21^−^CD27^−^ immature B cells, CD22^+^CD27^−^ naive B cells, CD20^−^CD27^+^ plasmablasts and CD22^+^CD27^+^ memory B cells. Memory B cells were further divided into CD27^+^IgD^+^ pre-class switched memory B cells and CD27^+^IgD^−^ class switched memory B cells. Furthermore, in patients with SLE, CD11c^+^CD21^low^ activated memory B cells were determined.

### Statistics

The endpoints were based on safety and efficacy according to a Bryant and Day two-stage optimal design^21^. The null hypothesis, that the true toxicity rate is 50%, was tested against a one-sided alternative of an acceptable toxicity rate of 20% or lower. At the same time, a null hypothesis that the true response rate is 30% was tested against a one-sided alternative of a response rate equal to or higher than 60%. The statistical tests were performed accepting a one-sided type I error (*α*) of 0.05 and a type II error (*β*) of 0.20 (power 80%). In phase 1, eight patients were recruited, with stopping rules for relevant toxicity and inadequate response. Relevant toxicity was defined by more than four patients experiencing a DLT event. Inadequate response was defined as fewer than four patients reaching the aforementioned efficacy endpoints. If no more than four patients experienced relevant toxicities and at least four patients reached the efficacy endpoint, phase 2a of the study would start with the inclusion of an additional sixteen patients. Stopping rules in phase 2a were defined as more than six patients experiencing relevant toxicities as described above. Quantitative variables were summarized as medians and IQRs; categorical items were presented as absolute counts and percentages. The Wilcoxon signed-rank test was used to assess differences in parameters between baseline and the 6-month evaluation. Power calculations for design and statistical analyses were performed with R (version 4.0.1) software.

### Reporting summary

Further information on research design is available in the Nature Portfolio Reporting Summary linked to this article.

## Online content

Any methods, additional references, Nature Portfolio reporting summaries, source data, extended data, supplementary information, acknowledgements, peer review information; details of author contributions and competing interests; and statements of data and code availability are available at 10.1038/s41591-025-04185-6.

## Supplementary information

Supplementary Information Supplementary Figs. 1 and 2, Supplementary Tables 1−3 and CASTLE Study Protocol

Reporting Summary

## Source data

Source Data Raw data for figures

## Data Availability

All numeric data of the figures of this paper are in the source data file. Additional information can be obtained from the corresponding author upon reasonable request via email to georg.schett@uk-erlangen.de within 6 weeks. Patient data can be shared only in pseudonymized form; otherwise, there are no restrictions to data access. Source data are provided with this paper.
