# Supplementary material for: CD19 CAR-T cells for treatment-refractory autoimmune diseases: the phase 1/2 CASTLE basket trial
Source: Nat Med. 2026 Jan 7;32(3):1142–51. doi: 10.1038/s41591-025-04185-6 (PMC13004673; doi:10.1038/s41591-025-04185-6)
Supplement: Supplementary file 1 — Supplementary Figs. 1 and 2, Supplementary Tables 1−3 and CASTLE Study Protocol [file 41591_2025_4185_MOESM1_ESM.pdf]

nature medicine

Supplementary information https://doi.org/10.1038/s41591-025-04185-6

CD19 CAR-T cells for treatment-refractory autoimmune diseases: the phase 1/2 CASTLE basket trial

In the format provided by the authors and unedited

Supplementary Material

Table of contents	page 1
Supplementary Figure 1	page 2
Supplementary Figure 2	page 3
Table S1	pages 4-7
Table S2	page 8
Table S3	page 9
CASTLE Study Protocol	pages 10-148

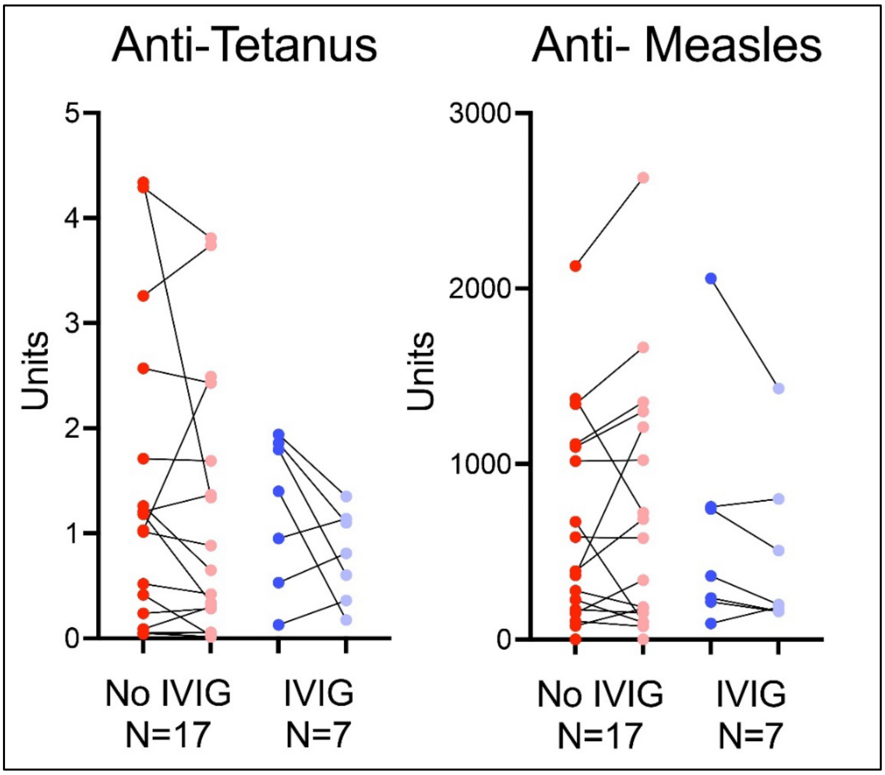

Suppl. Figure 1. Vaccination antibodies related to IVIG substitution.

Graph shows the course of anti-tetanus and anti-measles titers pre and post CD19 CAR-T cell therapy in respect to substitution of intravenous Immunoglobulin (IVIG). No significant difference can be observed between patients receiving or not receiving IVIG.

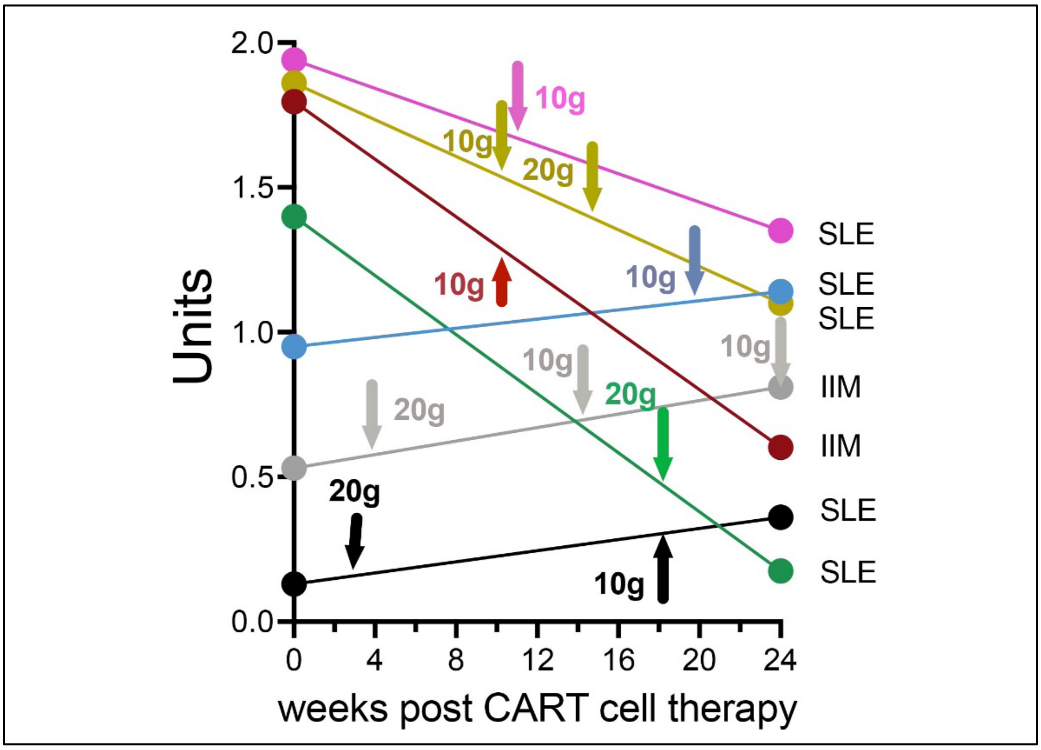

Suppl. Figure 2. Anti-tetanus antibody responses and timing and doses of IVIG-substitution.

Graph shows detailed anti-tetanus antibody response (units) in seven patients that received intravenous immunoglobulins post CD19 CAR-T cell therapy (5 patients with systemic lupus erythematosus (SLE), 2 patients with idiopathic inflammatory myositis (IIM)). Doses of IVIG are marked by arrows. IVIG substitution has no impact on anti-tetanus antibody response.

Suppl. Table 1: Adverse events (1)

CTCAE category and AE term	All	SLE	SSc	IIM
**Blood and lymphatic system disorders**	98	66	17	15
Anemia	19	14	2	3
Leukopenia	35	26	4	5
Leukocytosis	2	1	1	0
Lymphopenia	3	2	1	0
Neutropenia	34	20	9	5
Pancytopenia	1	1	0	0
Thrombocytosis	1	0	1	0
Thrombopenia	8	6	0	2
**Cardiac disorders**	8	3	3	2
Tachycardia	4	2	1	1
Palpitation	2	1	0	1
Suspected Myocarditis	1	0	1	0
Atrial Fibrillation	1	0	1	0
**Endocrine disorders**	1	1	0	0
Adrenal insufficiency	1	1	0	0
**Eye disorders**	1	1	0	0
Unspecific pain	1	1	0	0
**Gastrointestinal disorders**	42	18	16	8
Abdominal pain	3	2	-	1
Diarrhea	8	1	5	2
Dysphagia	2	1	1	-
Hemorrhoids	2	-	2	-
Koprostasis	1	1	-	-
Meteorism	1	1	-	-
Mucositis	2	2	-	-
Nausea	14	7	4	3
Obstipation	2	1	-	1
Tooth pain	1	1	-	-
Paradontitis	1	-	1	-
Perianal ulceration	1	-	1	-
Reflux	3	1	2	-
Dry mouth	1	-	-	1
**General/administration site conditions**	19	7	9	3
Edema	6	3	2	1
Chills	1	0	1	0
Pain	2	1	1	0
Fatigue	5	2	2	1
Fever	2	0	2	0
Lymphadenopathy	2	0	1	1
Injection site pain	1	1	0	0
**Hepatobiliary disorders**	1	1	0	0
Acalculous cholezystitis	1	1	0	0

Suppl. Table 1: Adverse events (2)

CTCAE category and AE term	All	SLE	SSc	IIM
**Immune system disorders**	49	27	16	6
Anaphylaxis	1	1	0	0
Cytokine release syndrome	18	9	6	3
Hypogammaglobulinemia	30	17	10	3
**Infections and infestations**	60	26	18	16
CMV reactivation with pneumonia	1	1	0	0
Community acquired pneumonia	3	1	2	0
SARS-Cov2 Infection	5	2	1	2
Digital wart	1	1	0	0
Gastroenteritis	2	1	1	0
Herpes simplex labialis	3	2	0	1
Infection with unknown focus	3	1	2	0
Influenza A infection	1	1	0	0
Sinusitis	4	1	3	0
Upper respiratory infection	25	10	8	7
Urinary tract infection	5	2	1	2
Vaginal mykosis	3	1	0	2
Oral Infections	5	3	1	1
Perianal ulceration	1	0	1	0
**Injuries**	1	0	1	0
Serial rib fractures	1	0	1	0
**Investigations**	19	13	2	4
Creatin kinase increased	3	2	0	1
Decreased CD4+ T-cells grade 3	1	1	0	0
Transaminases increased	2	2	0	0
Hyperlipidemia	1	1	0	0
Hypertriglyceridemia	1	1	0	0
Creatinine increased	2	1	0	1
CRP increased	3	1	1	1
NT-proBNP increased	1	1	0	0
INR increased	1	0	0	1
Leukocyturia	1	0	0	1
Microhematuria	1	0	0	1
Positive blood culture (contamination)	1	1	0	0
Proteinuria	1	1	0	0

Suppl. Table 1: Adverse events (3)

CTCAE category and AE term	All	SLE	SSc	IIM
**Meatbolism and nutrition disorders**	9	6	1	2
Folic acid deficiency	2	2	0	0
Hypokalemia	3	2	0	1
Hyponatremia	1	0	0	1
Iron deficiency	3	2	1	0
**Musculoskeletal disorders**	27	14	6	7
Arthralgia	10	3	3	4
Arthritis	1	0	0	1
Back pain	2	1	0	1
Bursitis	1	0	1	0
Costal pain	1	0	1	0
SLE flare (before ATMP administration)	4	4	0	0
Polyserositis in context of SLE flare	1	1	0	0
Mucositis win context of SLE flare	1	1	0	0
Rash in context of SLE flare	1	1	0	0
Morning stiffness joints	1	1	0	0
Muscle cramps	1	1	0	0
Myalgia	2	0	1	1
Polychondritis	1	1	0	0
**Neoplasms benign, malignant**	0	0	0	0
**Nervous system disorders**	18	10	2	6
Dizziness	3	2	0	1
Anosmia	1	1	0	0
Headache	8	4	1	3
Neuropathic pain feet	1	0	0	1
Parestesia	1	0	1	0
Diplopia (transient)	1	1	0	0
Transient ischemic attack	1	1	0	0
Tremor hands	1	0	0	1
Visual disturbance (transient)	1	1	0	0

Suppl. Table 1: Adverse events (4)

CTCAE category and AE term	All	SLE	SSc	IIM
**Pregnancy**	0	0	0	0
**Psychiatric disorders**	5	4	0	1
Nightmares	1	1	0	0
Insomnia	3	2	0	1
Concentration impairment	1	1	0	0
**Renal and urinary disorders**	10	10	0	0
Acute kidney injury	9	9	0	0
Intra- and postrenal urinary obstruction	1	1	0	0
**Reproductive system and breast**	2	1	0	1
Dysmenorrhea	1	1	0	0
Menopausal symptoms	1	0	0	1
**Respiratory and thoracic disorders**	3	2	0	1
Coughing	1	1	0	0
Pleural effusion with dyspnoe	2	1	0	1
**Skin and subcutaneous tissue disorders**	35	21	7	7
Alopecia	7	4	1	2
Contact eczema	2	1	0	1
Ulceration	3	1	2	0
Rash	6	5	1	0
Exanthema face	1	0	0	1
Pruritus	5	3	1	1
Mucositis	3	3	0	0
Scaling of skin	2	0	2	0
Subungual hemorrhage	1	1	0	0
Skin lesions, other	4	3	0	1
Teleangiectasia	1	0	1	0
**Surgical and medical procedures**	2	2	0	0
Postinterventional (ovariectomy) pain	1	1	0	0
Ulceration after Shaldon catheter	1	1	0	0
**Vascular disorders**	4	2	1	1
Orthostatic disorder	2	1	0	1
Hypotension	1	0	1	0
Hypertension	1	1	0	0

Suppl. Table 2A. Serious Adverse Events

SAE	Disease	IMP-related	Resolved
**Infections**			
SARS-CoV2 pneumonia	IIM	No	Yes
CMV Pneumonia*	SLE	Yes	Yes
Non-specified pneumonia	SSc	Yes	Yes
Infection of unknown origin	SSc	No	Yes
**Hematopoietic System**			
Neutropenia Grade 4	SLE	Yes	Yes
Neutropenia Grade 4	SLE	Yes	Yes
Neutropenia Grade 4	SLE	Yes	Yes
Neutropenia Grade 4	SLE	Yes	Yes
**Neurologic Disorders**			
Suspected TIA	SLE	Yes	Yes
**Immune System Disorder**			
CRS I (prolonged hospital stay)	SSc	Yes	Yes
CRS I (prolonged hospital stay)	SLE	Yes	Yes
**Gastrointestinal disorders**			
Acalculous cholezystitis*	SLE	No	Yes
Abdominal pain	SLE	Yes	Yes
**Renal and urinary disorders**			
Acute kidney injury grade 4*	SLE	Yes	Yes**
Increased creatinine (prolonged hospital stay)	SLE	Yes	Yes

* SAE occurred in the same patients; ** resolution with sequeleae; CRS, cytokine release syndrome CMV, cytomegaly virus; SARS-CoV2, severe respiratory distress syndrome coronavirus 2.

Suppl. Table 2B: Adverse events of special interest (AESI)

CTCAE category and AE term	All	SLE	SSc	IIM	Resolved
**CRS**	18	9	6	3	Yes
CRS grade 1	17	9	5	3	
CRS grade 2	1	0	1	0	
Flare before ATMP administration	4	4	0	0	Yes
Acute Kidney Injury	1	1	0	0	Yes*

CRS, cytokine release syndrome; CTCAE, Common Terminology Criteria for Adverse Events.

Suppl. Table 3. Clinical outcomes before and after CAR T-cell therapy

	Baseline	6 months	p
**N (SLE)**	**10**	**10**	
SLEDAI (score), median [IQR]	12.5 [9.0; 14.0]	0 [0; 0]	P <0.0001
BILAG (score), median [IQR]	20.0 [18.0; 23.0]	0 [0.0; 1.0]	P <0.0001
**N (SSc)**	**9**	**9**	
mRSS (score), median [IQR]	26.0 [17.0; 30.5]	12.0 [8.0; 21.5]	P <0.0001
FVC (L), median [IQR]	2.83 [2.71; 3.96]	3.23 [3.06; 4.47]	P = 0.0054
DLCO (L), median [IQR]	4.23 [3.47;6.07]	5.48 [4.31; 7.18]	P = 0.0076
**N (IIM)**	**5**	**5**	
MMT8 (score), median [IQR]	117 [76; 138]	147 [128; 150]	P = 0.06
CK (units/L), median [IQR]*	1948 [1405; 2212]	200 [127; 975]	P = 0.019

BILAG, British Isles Lupus Assessment Group; CK, creatine kinase; DLCO, diffusion capacity of carbon monoxide; FVC, forced vital capacity; IIM, idiopathic inflammatory myositis; SLE, systemic lupus erythematosus; MMT8, manual muscle testing 8; mRSS, modified Rodnan skin score; SLEDAI, SLE disease activity index; SSc, systemic sclerosis; *assessed in the 3 patients with elevated CK at baseline.

Clinical Study Protocol

__CA__R-T cells in __S__ys__T__emic B cel__L__ mediated autoimmun__E__ Disease - __CASTLE__

Protocol version	*5.1*
Date	*15.12.2023*
Study code	*CASTLE*
EudraCT-Nr.	*2022-001366-35*
Study short title	*CASTLE*
Sponsor	*Universitätsklinikum Erlangen*<br>*represented by the Dean*<br>*Medizinische Fakultät*<br>*Friedrich-Alexander-Universität Erlangen-Nürnberg*<br>*Maximiliansplatz 2*<br>*91054 Erlangen*<br>*Phone: +49 9131 85 47047*<br>*Fax: +49 9131 85 35120*
Sponsor Designee	*Prof. Dr. med. Georg Schett*<br>*Chairman Medizinische Klinik 3*<br>*Universitätsklinikum Erlangen*<br>*Ulmenweg 18*<br>*91054 Erlangen*

Confidential and proprietary

The contents of this document are confidential and proprietary to the Sponsor.

Unauthorized use, disclosure or reproduction is strictly prohibited. This document or parts thereof may not be disclosed to parties not associated with the clinical investigation without the prior written consent of the Sponsor.

Gender-specific notation is omitted in favor of the readability and comprehensibility of the protocol.

Signature Pages

Sponsor

Date	*Prof. Dr. med. Markus F. Neurath*
	Dekan (Dean), Medizinische Fakultät<br>*Friedrich-Alexander-Universität Erlangen-Nürnberg*

Sponsor Designee

Date	*Prof. Dr. med. Georg Schett*

Chairman Medizinische Klinik 3

Universitätsklinikum Erlangen

Ulmenweg 18

91054 Erlangen

Site Principle Investigator

By my signature, I agree to personally supervise the conduct of this study and to ensure its conduct in compliance with the protocol, informed consent, the Declaration of Helsinki, ICH Good Clinical Practices guidelines, and the applicable parts of the local regulations governing the conduct of clinical studies.

Date	*Prof. Dr. med. Georg Schett*

Chairman Medizinische Klinik 3

Universitätsklinikum Erlangen

Ulmenweg 18

91054 Erlangen

List of Abbreviations

**ACPA**	Anti-Citrullinated Protein Antibodies
**ACR**	American College of Rheumatology
**AD**	Available Data
**AE(SI)**	Adverse Event (of Special Interest)
**ALC**	Absolute Lymphocyte Count
**ALT (SGPT)**	Alanine transaminase (Serum glutamate pyruvic transaminase)
**AMG**	Arzneimittelgesetz (German Drug Law)
**ANA**	Antinuclear Antibodies
**ANC**	Absolute Neutrophil Count
**ALC**	Absolute Lymphocyte Count
**AR**	Adverse Reaction
**AST (SGOT)**	Aspartate transaminase (serum glutamic oxaloacetic transaminase)
**ATC**	Anatomical Therapeutic Chemical (ATC) Classification
**ATMP**	Advanced Therapy Medicinal Product
**BAFF**	B cell activating factor
**BILAG**	British Isles Lupus Assessment Group
**BL**	Baseline
**BW**	Body Weight
**CA**	Competent Authority
**CAR**	Chimeric Antigen Receptor
**CCS**	Center for Clinical Studies
**CD**	Cluster of Differentiation
**CNS**	Central Nervous System
**COX**	Cyclooxygenase
**CRF**	Case Report Form (may be implemented as paper-based CRF)
**CRO**	Clinical Research Organisation
**CRP**	C-reactive protein
**CRS**	Cytokine-release syndrome
**CSF**	Cerebrospinal Fluid
**CSP**	Clinical Study Protocol
**CTA**	Clinical Trial Authorization
**CTCAE**	Common Toxicity Criteria for Adverse Events
**CTM**	Clinical Trial Manager

**DLCO**	Diffusion Capacity of the Lungs
**DLT**	Dose Limiting Toxicity
**DM**	Dermatomyositis
**DMARD**	Disease Modifying Anti-Rheumatic Drug
**DMC**	Data Monitoring Committee
**(ds)DNA**	(double-stranded) Deoxyribonucleic Acid
**DSUR**	Development Safety Update Report
**EC**	Ethics Committee
**ECG**	Electrocardiogram
**EMA**	European Medicines Agency
**ENA**	Extractable Nuclear Antigen
**ESR**	Erythrocyte Sedimentation Rate
**EU**	European Union
**EudraCT**	European Union Drug Regulating Authorities Clinical Trials
**EULAR**	European League Against Rheumatism
**FACIT**	Functional Assessment of Chronic Illness Therapy
**FBC**	Full Blood Count
**FCBP**	Females of Childbearing Potential
**FSFV**	First Subject First Visit
**FU**	Follow Up
**FVC**	Forced Vital Capacity
**GC**	Glucocorticoid
**GCP**	Good Clinical Practice
**GCP-V**	GCP-Verordnung
**GFR**	Glomerular Filtration Rate
**GGT**	Gamma-Glutamyl Transferase (transpeptidase)
**GMP**	Good Manufacturing Practice
**HAQ-DI**	Health Assessment Questionnaire – Disability Index
**HIV**	Human Immunodeficiency Virus
**ICANS**	Immune-effector Cell Associated Neurotoxicity Syndrome
**ICD**	International Classification of Diseases
**ICE**	Immune Effector Cell-associated Encephalopathy
**ICF**	Informed Consent Form
**ICH**	International Conference on Harmonization
**ICMJE**	International Committee of Medical Journal Editors

**ICU**	Intensive Care Unit
**IEC**	Independent Ethics Committee
**IFM**	*IT für Forschung & Management*
**IFN**	Interferon
**IL**	Interleukin
**INR**	International Normalized Ratio
**IMP**	Investigational Medicinal Product
**IMPD**	Investigational Medicinal Product Dossier
**IPC**	In-Process-Control
**IRB**	Institutional Review Board
**ISF**	Investigator Site File
**IUD**	Intrauterine Device
**IUS**	Intrauterine hormone-releasing System
**LDH**	Lactat Dehydrogenase
**LLDAS**	Lupus Low Disease Activity State
**LOCF**	Last Observation Carried Forward
**LSLV**	Last Subject Last Visit
**MedDRA**	Medical Dictionary for Regulatory Activities
**MIK**	Medical Center for Information and Communication Technology
**MMF**	Mycophenolate Mofetil
**MMT**	Manual Muscle Test
**MPA**	Microscopic Polyangiitis
**MRI**	Magnetic Resonance Imaging
**mRSS**	Modified Rodnan Skin Score
**N**	Number
**NK**	Natural Killer Cell
**NSAID**	Non-Steroidal Anti-Inflammatory Drug
**ORR**	Overall Response Rate
**PEI**	Paul-Ehrlich-Institut
**PhGA**	Physician’s Global Assessment
**PM**	Polymyositis
**PRO**	Patient Reported Outcomes
**PtGA**	Patient’s Global Assessment
**PTT**	Partial Thromboplastin Time
**RF**	Rheumatoid Factor

**RNA**	Ribonucleic Acid
**SAE**	Serious Adverse Event
**SAP**	Statistical Analysis Plan
**SAR**	Serious Adverse Reaction
**SC**	Subcutaneous
**SDV**	Source Data Verification
**SIN**	Self-inactivating
**SLE**	Systemic Lupus Erythematosus
**SLEDAI**	Systemic Lupus Erythematosus Disease Activity Index
**SMB**	Safety Management Board
**SSC**	Systemic Sclerosis
**SUSAR**	Suspected Unexpected Serious Adverse Reaction
**TB(C)**	Tuberculosis
**TLC**	Total Lung Capacity
**TNF**	Tumor Necrosis Factor
**ULN**	Upper Limit of Normal
**V**	Visit
**VAS**	Visual Analogue Scale

Table of Contents

Signature Pages 11
List of Abbreviations 14
1 SYNOPSIS 25
2 ASSESSMENT SCHEDULE 30
3 STUDY ADMINISTRATIVE STRUCTURE 34
4 BACKGROUND INFORMATION 36
4.1 Investigational product 36
4.1.1 Background information on the Investigational Product 36
4.1.2 Role of B cells in systemic autoimmune diseases 36
4.1.3 Indication 37
4.1.3.1 Systemic Lupus Erythematosus (SLE) 38
4.1.3.2 Systemic sclerosis (SSc) 38
4.1.3.3 Dermatomyositis/Polymyositis (DM/PM) 39
4.2 Preliminary data 39
4.2.1 Preclinical models 39
4.2.2 Clinical data 41
4.3 Rationale 43
4.3.1 Rationale of the study 44
4.3.2 Study design 46
4.3.3 Selection of dose and dose regimen 46
4.3.4 Lymphodepleting chemotherapy 46
4.3.5 Subject population 47
4.4 Risk-benefit assessment 47
4.4.1 Assumed benefits for the subjects 47
4.4.2 Anticipated risks for the subjects 48
4.4.2.1 Risks due to study-related procedures 48
4.4.2.2 Risks due to lymphodepleting chemotherapy 48
4.4.2.3 Risks due to CAR T cell therapy 48
4.4.3 Benefit-risk ratio 49
5 STUDY OBJECTIVES 50
5.1 Primary Objective 50
5.1.1 Primary Endpoint 50
5.2 Secondary Objectives 50
5.2.1 Secondary endpoints 50
5.2.1.1 Clinical efficacy 50
5.2.1.2 Cellular and humoral response 51
5.2.1.3 Additional endpoints 51
5.3 Exploratory objectives 52
5.4 Complementary Research Program 52
6 INVESTIGATIONAL PLAN 53
6.1 Overall study design 53

6.1.1 Study flow chart 56
6.1.2 Number of subjects 56
6.1.3 Study duration 56
6.1.4 Inclusion procedure 56
6.1.5 Definition of a Dose Limiting Toxicity 57
6.1.6 Management of toxicities 57
6.1.7 Stopping rules 58
6.1.7.1 Stage 1 58
6.1.7.2 Stage 2 58
6.1.8 Safety Management Board 59
6.1.9 End of study 59
6.2 Treatment procedure 59
6.2.1 Screening (Visit 1) 60
6.2.2 Leukapheresis (Visit 2) 63
6.2.3 Lymphodepletion (Visit 3) 63
6.2.3.1 Prior to lymphodepletion procedure 63
6.2.3.2 Days -4 and -3 64
6.2.4 Treatment phase (Visit 4) 65
6.2.4.1 Day 0, pre-dose 65
• Check of additional criteria before IMP administration (see section 7.3.4) 65
6.2.4.2 Day 0, dosing 66
6.2.4.3 Day 0, post-dose (1 and 2 hours) 66
6.2.4.4 Day 0, post-dose (4 hours) 66
6.2.5 Short-term Follow-up (Visit 5-10) 66
6.2.6 Mid-term Follow-up (Visits 11-14) 67
6.2.6.1 Week 8 (Visit 11) 67
6.2.6.2 Week 12 (Visit 12) 68
6.2.6.3 Week 16 (Visit 13) 69
6.2.6.4 Week 24 (Visit 14) 69
6.2.7 Additional Response-Assessment Visit 70
6.2.8 Safety Follow-up Visits (e.g. for flare of disease) 70
6.2.9 Unscheduled Visits 71
6.2.10 Continuation of Treatment after the End of the Clinical Study 71
6.2.11 Complementary Research Program 72
7 STUDY POPULATION 73
7.1 Selection of study population 73
7.2 Gender distribution 73
7.3 Inclusion criteria 73
7.3.1 General inclusion criteria 73
7.3.2 Disease-specific inclusion criteria 74

7.3.2.1 SLE subjects 74
7.3.2.2 SSc subjects 74
7.3.2.3 DM/PM subjects 75
7.3.3 Additional criteria before lymphodepletion 75
7.3.4 Additional criteria before IMP administration 76
7.4 Exclusion criteria 76
7.5 Assignment to treatment 77
7.6 Subject completion and discontinuation 78
7.6.1 Definitions 78
7.6.2 Handling of flare of disease 78
7.6.3 Procedure for handling discontinuations 78
8 STUDY MEDICATION AND ADMINISTRATION 80
8.1 Lymphodepletion 80
8.2 Investigational Medicinal Product MB-CART19.1 80
8.2.1 Manufacturing of the IMP 81
8.2.1.1 Leukapheresis (Day -13) 81
8.2.1.2 Preparation (Day -12 to -1) 81
8.2.1.3 Packaging 81
8.2.1.4 Labeling 81
8.2.1.5 Storage 81
8.2.2 Transport of the IMP 82
8.2.3 Dosage of the IMP (Day 0) 82
8.2.3.1 Dosing schedule: single dose i.v. 82
8.2.3.2 Measures in case of “failed” In-Process-Control (IPC) during Manufacturing ... 82
8.2.3.3 Measures in case subject is unable to be dosed on Day 0 82
8.2.3.4 Measures in case of insufficient cell numbers of IMP 83
8.2.3.5 Measures in case of IMP being Out Of Specification (OOS) 83
8.2.4 Preparations and administration of IMP 84
8.3 Method of blinding 84
8.4 Drug accountability 84
8.5 Auxiliary Medicinal Products / Non-Investigational Medicinal Products 85
8.6 Concomitant medication 85
8.6.1 Concomitant medication at Screening 86
8.6.2 Concomitant medication during lymphodepletion 86
8.6.3 Prohibited concomitant medication 87
8.6.4 Concomitant medication in case of flare 87
8.7 Rescue medication 87
9 METHODS OF ASSESSMENTS 88
9.1 Medical history and concomitant medication 88
9.2 Disease activity assessments 88

9.2.1 General assessments and questionnaires 88
9.2.1.1 Patient's and Physician's Global Assessment (PtGA/ PhGA) of disease activity 88
9.2.1.2 Health Assessment Questionnaire-Disability Index (HAQ-DI)....88
9.2.1.3 Functional Assessment of Chronic Illness Therapy – Fatigue (FACIT Fatigue) 89
9.2.1.4 EORTC QLQ-C30 89
9.2.2 SLE specific assessments and questionnaires 89
9.2.2.1 British Isles Lupus Assessment Group (Easy-BILAG) index....89
9.2.2.2 Systemic Lupus Erythematosus Disease Activity Index (SLEDAI)....89
9.2.3 SSC specific assessments and questionnaires....90
9.2.3.1 modified Rodnan Skin Score (mRSS) 90
9.2.4 DM/PM specific assessments and questionnaires 90
9.2.4.1 Physician's Global Assessment (PhGA) of extramuscular activity....90
9.2.4.2 Manual Muscle Testing (MMT)....90
9.3 Safety assessments....90
9.3.1 Laboratory assessments 90
9.3.1.1 Safety laboratory....91
9.3.1.2 Study-specific laboratory....91
9.3.1.3 Other testings 92
9.3.2 B cell Depletion and Ig Levels 92
9.3.3 Clinical safety 93
9.3.3.1 Vital signs 93
9.3.3.2 Lung function 93
9.3.3.3 Brain MRI....93
9.3.3.4 ECG and echocardiography....93
9.3.4 Physical examination....93
9.3.5 Persistence and phenotyping of infused MB-CART19.1 94
9.3.6 Physical function 94
9.4 Complementary research program 95
9.5 Total amount of blood 95
10 SAFETY ASSESSMENTS 97
10.1 Adverse Events 97
10.1.1 Definition of Adverse Events....97
10.1.2 Reporting of Adverse Events....97
10.1.2.1 Required information....98
10.1.2.2 Intensity 98
10.1.2.3 Causal relationship 99
10.1.2.4 Expectedness 99
10.1.2.5 Actions taken 99
10.1.2.6 Outcome 100
10.2 Serious Adverse Events....100

10.2.1 Definition of Serious Adverse Events 100
10.2.2 Reporting of Serious Adverse Events 102
10.2.3 Sponsor´s assessment of Serious Adverse Events 103
10.3 Adverse Events of Special Interest 104
10.4 Toxicities related to lymphodepleting therapy 104
10.5 Breaking the blind 105
10.6 Pregnancy 105
10.6.1 Contraception 105
10.6.2 Reporting of pregnancy 106
10.7 Premature termination of the study 106
10.8 Development Safety Update Report (DSUR) 107
10.9 Communication Plan of Safety Data 107
11 STUDY MANAGEMENT AND ADMINISTRATION 108
11.1 Adherence to protocol 108
11.2 Local data and biobank (TARDA = Translational Arthritis Research Database) 108
11.3 Source Documentation 108
11.4 Case Report Form 109
11.5 Monitoring 109
11.6 Source data verification 110
11.7 Archiving and data retention 110
12 STATISTICAL METHODS 111
12.1 Statistical evaluations 111
12.1.1 Study variables 111
12.1.1.1 Primary safety outcome variable 111
12.1.1.2 Secondary outcome variable 111
12.1.1.3 Exploratory variables 112
12.1.2 Analysis population 112
12.2 Determination of sample size 112
12.3 Statistical analysis 113
12.3.1 Dataset to be analysed 113
12.3.2 Analysis of safety 113
12.3.3 Analysis of efficacy 113
12.4 Missing Data 114
13 ETHICS AND REGULATORY 115
13.1 Investigator obligations 115
13.2 Ethics Committee and Competent Authority 115
13.3 Patient Information and Informed Consent 116
13.4 Duration of the study 117
13.5 Subject privacy 117
13.6 Insurance 117
13.7 Publication 118

14 APPENDICES 119
14.1 Grading of CRS 119
14.2 Management of CRS 120
14.3 Grading of ICANS 121
14.4 Management of ICANS 122
14.5 Grading of CAR T cell specific organ toxicity and action 123
14.6 Patient's and Physician's Global Assessment (PtGA/ PhGA) of disease activity ... 126
14.7 Health Assessment Questionnaire-Disability Index (HAQ-DI) 127
14.8 Functional Assessment of Chronic Illness Therapy – Fatigue (FACIT Fatigue) 129
14.9 QLQ-C30 130
14.10 British Isles Lupus Assessment Group (BILAG) 2004 index 132
14.11 Systemic Lupus Erythematosus Disease Activity Index 2000 (SLEDAI-2K) 134
14.12 DORIS remission criteria of SLE 135
14.13 modified Rodnan Skin Score (mRSS) 136
14.14 Physician's Global Assessment (PhGA) of extramuscular activity 136
14.15 Manual Muscle Testing (MMT) 137
14.16 2016 ACR/EULAR Criteria for Clinical Response in Adult DM/PM 139
14.17 2019 ACR/EULAR classification criteria of SLE 140
14.18 2013 ACR/EULAR classification criteria of SSc 141
14.19 2017 ACR/EULAR classification criteria for probable or definite DM or PM 142
15 REFERENCES 143

List of Figures

Figure 1: Overall study design. 54
Figure 2: Study flow chart. 56
Figure 3: Schematic overview of study procedure. 60

List of Tables

Table 1: Characterization of the three diseases indicated for the study treatment. 38
Table 2: Current status of CAR T cell treatment in autoimmune disease with safety and efficacy data. 43
Table 3: Total amount of blood per visit. 95

1 SYNOPSIS

<table>
<tr><td>Protocol title</td><td>CAR-T cells in SysTemic B celL mediated autoimmunE Disease</td></tr>
<tr><td>Short titel</td><td>CASTLE</td></tr>
<tr><td>Study phase</td><td>I/II</td></tr>
<tr><td>Indication</td><td>refractory systemic autoimmune disease</td></tr>
<tr><td>Study design</td><td>a prospective, open-label, single-dose, non-randomized, interventional basket trial</td></tr>
<tr><td>Study objectives</td><td><u>Primary Objective</u>
• To assess the safety of anti-CD19 CAR T cell therapy in subjects with active B-driven autoimmune disease (SLE, SSc and DM/PM).
<u>Primary endpoint</u>
• Incidence and grading of severity (graded 0-4) of Cytokine Release Syndrome (CRS) and of CAR T cell Associated Neurotoxicity Syndrome (ICANS) within the first 4 weeks after ATMP administration.
<u>Secondary Objectives</u>
• To assess the clinical efficacy of anti-CD19 CAR T cell therapy in subjects with active B-driven autoimmune disease (SLE, SSc and DM/PM).
• To investigate the duration of B cell depletion after anti-CD19 CAR T cell administration.
• To investigate the duration of CAR T cell persistence after anti-CD19 CAR T cell administration.
• To investigate the changes in the levels of disease-associated serum autoantibodies.
• To investigate the in vivo cellular kinetics of MB-CART19.1, immunogenicity and feasibility of the manufacturing process.
<u>Secondary endpoints</u>
Clinical efficacy
• Overall Response Rate (ORR) at week 24 measured by specific disease activity composite indexes, each of them validated for the specific disease:
- SLE: Fulfillment of DORIS remission criteria of SLE at week 24.
- SSc: No progression of interstitial lung disease with worsening of FVC1 (>10%) or worsening of FVC1 (5-10%) plus increase in respiratory symptoms or worsening of FVC1 (5-10%) plus progression of high-resolution computed tomography changes after 24 weeks.
- DM: 2016 ACR/EULAR Moderate or Major Response. No progression of interstitial lung disease with worsening of FVC1 (>10%) or worsening of FVC1 (5-10%) plus increase in respiratory symptoms or worsen-ing of FVC1 (5-10%) plus progression of high-resolution computed tomography changes after 24 weeks.
Cellular and humoral response
• Duration of persistence of CAR T cells in the peripheral blood
• Duration of B cell depletion in the peripheral blood
• Levels of respective serum autoantibodies at week 24 including incidence of sero-conversion
- SLE: ANA, anti-dsDNA, anti-nucleosomes, anti-Sm, anti-cardiolipin IgG, C3 C4
- SSc: ANA, anti-SCL70, anti-RNA polymerase III, anti-topoisomerase
- DM: ANA, anti-Mi2, anti-Tif1, anti-MDA5, anti-Jo1, anti-NXP2
• Expansion of CAR T cells in the patient over time
• Success of the manufacturing process by GMP certification of the product
Additional endpoints
• General:</td></tr>
</table>

	- Patient's Global Assessment (PtGA) of disease activity (VAS 0-100mm)<br>- Physician's Global Assessment (PhGA) of disease activity (VAS 0-100mm)<br>- Health Assessment Questionnaire – Disease Index (HAQ-DI)<br>- Functional Assessment of Chronic Illness Therapy - Fatigue (FACIT Fatigue)<br>- Core Quality of Life (EORTC QLQ-C30)<br>• SLE:<br>- British Isles Lupus Assessment Group (BILAG) index<br>- Systemic Lupus Erythematosus Disease Activity Index (SLEDAI)<br>• SSc:<br>- modified Rodnan Skin Score (mRSS)<br>• DM/PM:<br>- Physician's global assessment (PhGA) of extramuscular activity<br>- Manual Muscle Testing (MMT)<br>**Exploratory objectives**<br>• To analyze the changes in B cell receptor repertoire.<br>• To evaluate the therapy-induced alterations of B compartments (permitting to evaluate the "tolerogenic reset").
**Main inclusion criteria**	***General inclusion criteria***<br>• *Subjects must understand and voluntarily sign an informed consent form including written consent for data protection,*<br>• *Adults aged ≥ 18 years at time of consent,*<br>• Adequate renal (eGFR > 30 ml/min/m2), liver (no Child Pugh C), heart (at worst NYHA III, EF > 30%) and pulmonary (FV and DLCO ≥ 30%) function,<br>• *Male subjects unless surgically sterile, must agree to use two acceptable methods for contraception (e.g. spermicide and condom) during the trial and refrain from fathering a child starting from the time of signing the Informed Consent Form (ICF) until 12 months after dosing of the IMP,*<br>• *Females of childbearing potential (FCBP) must have a negative urine pregnancy test at screening and must agree to use a highly effective contraceptive method (Pearl index <1) starting from the time of signing the ICF and for 12 months after dosing of the IMP,*<br>• *Must be able to adhere to the study visit schedule and other protocol requirements,*<br>• *Double vaccination against SARS-CoV-2 or SARS-CoV-2-Infection within the last 6 months.*<br>***Disease-specific inclusion criteria***<br>• *SLE subjects*<br>- Fulfilling the 2019 ACR/EULAR classification criteria of SLE,<br>- Positivity of anti-dsDNA (> 4 U/l), anti-histone (+ or more), anti-nucleosome (+ or more) or anti-Sm antibodies (+ or more) at screening or by documented medical history,<br>- Active disease at screening, defined as ≥ 1 organ system with a British Isles Lupus Assessment (BILAG) A score (severe disease activity) or ≥ 2 organ systems with a BILAG B score (moderate disease activity),<br>- Insufficient response or intolerance/ contraindication to glucocorticoids and to at least 2 of the following treatments: azathioprine, hydroxychloroquine, mycophenolate mofetil, belimumab, methotrexate, *rituximab, cyclophosphamide*. Insufficient response is defined as having increased disease activity based on the definition explained in the previous bullet point.<br>- *SSc subjects*<br>- Fulfilling the 2013 ACR/EULAR classification criteria of SSc,

	- Positivity (+ or more) for at least one SSc-specific parameter (Scl70, RNA polymerase, Th/To, RP11/12, U3RNP autoantibodies) at screening or by documented medical history,<br>- Signs for fast progression including (i) disease duration ≤7 years (from onset of first non-Raynaud manifestation), (ii) mRSS score 10-35 at screening, (iii) elevated acute phase reactant levels (CRP ≥ 6 mg/L, ESR ≥ 28mm/h or platelet count ≥ 330 G/L), (iii) mRSS increase ≥ 3 units or involvement of one new body area or mRSS increase ≥ 2 units in one body area or ≥1 tendon friction rub over 6 months,<br>- Insufficient response or intolerance/ contraindication to at least 2 of the following treatments: *m*ycophenolate mofetil, *azathioprine, cyclophosphamide,* nintedanib, methotrexate*, rituximab*. Insufficient response is defined as having increased disease activity based on the definition explained in the previous bullet point.<br>- *DM/PM subjects*<br>- Fulfilling the 2017 ACR/EULAR classification criteria for probable or definite DM or PM,<br>- Presence of active myositis in muscle biopsy or muscle MRI and/or signs of interstitial lung disease related to DM/PM,<br>- Positivity (+ or more) for at least one myositis-specific antibody (aminoacyl tRNA synthetases, Mi2, MDA5, SAE, SRP, ARS, HMGCR, MJ, TIF1gamma) at screening or by documented medical history,<br>- In patients with active myositis: Muscle weakness as defined by MMT < 142 and 2 of the following criteria: VAS patients Global ≥ 2cm, VAS physician Global ≥ 2cm, HAQ > 0.25, at least one muscle enzyme > 1.3 times upper limit of normal, VAS global extra muscular activity ≥ 2cm,<br>- Insufficient response or intolerance/ contraindication to glucocorticoids and to at least 2 of the following treatments: azathioprine, *cyclophosphamide, m*ycophenolate mofetil, ciclosporin A, tacrolimus, methotrexate, rituximab, *intravenous immunoglobulins*. Insufficient response is defined as having increased disease activity based on the definition explained in the previous bullet point.
**Main exclusion criteria**	• *Clinical suitability for a less burdensome and/or approved therapeutic approach, as judged by the investigator,*<br>• *ANC < 1.000/mm^3, ALC < 500/mm^3 or hemoglobin < 8g/dl*<br>• *Uncontrolled severe concomitant disease, such as cancer (except basal or squamous cell skin cancer) and diabetes mellitus,*<br>• Severely impaired renal (eGFR ≤ 30 ml/min/m^2), liver (Child Pugh C), heart (NYHA IV, EF ≤ 30%) and pulmonary (FV and DLCO < 30%) function,<br>• *Any condition, including the presence of laboratory abnormalities, which places the subject at unacceptable risk if he/she were to participate in the study or confounds the ability to interpret data from the study,*<br>• Prior treatment with anti-CD19 antibody therapy, adoptive T cell therapy or any prior gene therapy product (e.g. CAR T cell therapy),<br>• History of bone marrow/ hematopoietic stem cell or solid organ transplantation,<br>• *Any concomitant severe active infection, e.g. HIV, hepatitis B or C, SARS-CoV 2 (COVID 19), or active tuberculosis as defined by a positive Quantiferon TB-test. If presence of latent tuberculosis is established then treatment according to local guidelines must have been initiated prior to enrollment,*<br>• Diagnosis of severe neuropsychiatric SLE, inclusion body myositis or limited SSc,<br>• *Pregnant or lactating females,*<br>• Females who are intending to conceive during the study,<br>• *Known hypersensitivity to any drug components,*<br>• *Malignancy in the last 5 years before screening,*

	• *Requirement for immunization with live vaccine during the study period or within 14 days preceding leukapheresis,*<br>• *Subjects who are younger than 18 years or are incapable to understand the aim, importance and consequences of the study and to give legal informed consent (according to § 40 Abs. 4 and § 41 Abs. 2 and Abs. 3 AMG),*<br>• *Have a history of alcohol or substance abuse within the preceding 6 months that, in the opinion of the Investigator, may increase the risks associated with study participation or study agent administration, or may interfere with interpretation of results,*<br>• *Subjects who possibly are dependent on the Sponsor, the Principal Investigator or Investigator (e.g. family members).*
**Interventions**	*Single intravenous infusion of a freshly produced Advanced Therapy Medicinal Product (ATMP) of autologous and expanded T cells that were ex vivo transduced with a CD19-CAR construct.*<br>*No control intervention (e.g. other immunosuppressive therapy).*<br>*Accompanying measures: leukapheresis and lymphodepleting therapy for conditioning.*
**Investigational product, dosage and route of administration**	*Fresh ATMP from autologous T cells ex vivo lentivirally transduced with the vector (anti-CD19 CAR pLTG1563) and expanded. Leukapheresis will be performed at the collection center according to local standard practice. The leukapheresis product of the subject will be used for the individual manufacturing of MB-CART19.1 by using the automated CliniMACS Prodigy System. CD4+ and CD8+ cells are enriched and activated, followed by lentivirus-based transduction with the CD19 CAR construct. Then the CD19 CAR transduced T cells are expanded and finally formulated (ATMP). ATMP manufacturing will be performed in certified locations under licenses by local regulatory authorities. T cells are activated, lentivirally transduced, expanded, and formulated. Quality controls are performed in-process and on the final product.*<br>Single infusion of freshly produced ATMP ($1x10^6$ autologous T cells/kg body weight) in infusion solution at a final volume adapted to the subjects' weight, over a time period of approx. 5 – 20 minutes (intravenous infusion via a large peripheral vein or central line).
**Procedures**	***Leukapheresis: Day -13***<br>*Leukapheresis will be performed inpatient according to local standard practice.*<br>***IMP-Manufacturing: Days -12 to -1***<br>*The leukapheresis product will be used for the individual manufacturing of CD19-CART by using the automated CliniMACS Prodigy System. CD4+ and CD8+ cells are enriched and activated, followed by lentivirus-based transduction with the CD19 CAR construct. Then the CD19 CAR transduced T cells are expanded and finally formulated.*<br>***Lymphodepleting therapy: Days -6 to -3***<br>*Lymphodepletion will be performed inpatient. All subjects will receive fludarabine 25 $mg/m^2/d$ intravenously (iv) on days -5, -4, and -3, and cyclophosphamide 1000 $mg/m^2/d$ iv on day -3 for lymphodepletion. A minimum interval of 48 hours after infusion of cyclophosphamide prior to administration of IMP has to be held.*<br>*For subjects with GFR between 30 and 70 ml/min the fludarabine dose will be reduced by up to 50% and the GFR of the subject will be monitored closely according to the institutional standards (at least once daily).*<br>***Experimental Intervention: Day 0***<br>*Subjects will receive a single infusion of freshly prepared CD19 CART.*<br>*Tocilizumab will be applied in dose 8 mg/kg BW if adverse events require damping of MB-CART19.1-mediated cytokine release and its symptoms. In case of adverse events, a strict reporting procedure will be established.*
**Follow-up per subject**	*The follow-up of the subjects will be performed in two steps:*<br>***Short-term: Days 1 to 28***<br>*Subjects will be inpatient at least until day 10 and followed-up closely with monitoring for drug-related toxicities, i.e. Cytokine Release Syndrome (CRS) and Immune-effector Cell Associated Neurotoxicity Syndrome (ICANS) until 28 days after treatment.* Blood samples for the determination of persistence and

	phenotype of infused CD19 CART will be taken. Circulating B cells will be measured, clinical and laboratory response will be assessed and AEs will be documented.<br>***<u>Mid-term: Weeks 4 to 24</u>***<br>*Until week 24, clinical and laboratory responses will be assessed. In addition, blood samples for the determination of persistence and phenotype of infused CD19 CART will be taken and the length of B cell depletion will be determined.* After the end of week 12, i.e., with start of week 13, only SAEs and AESI and their related concomitant medication will be documented and reported until week 24.
**Study duration per subject**	Including a screening period of up to 6 weeks, the study duration per subject is maximally 30 weeks.
**Number of visits**	14 visits
**Number of subjects**	24 subjects
**Planned study period**	Estimated start (First Subject First Visit, FSFV): May 2023<br>Estimated end (Last Subject Last Visit, LSLV): May 2026
**Statistical methods**	*The safety and efficacy endpoints are the Overall Response Rate (ORR) and the safety according to a Bryant and Day two stage optimal design:*<br>*Stage 1: 8 subjects will be accrued, with stopping rules for* relevant toxicity as defined by more than 4 subjects experiencing either CRS > grade 2 or ICANS > grade 2 or organ toxicity (cardiac, dermatologic, gastrointestinal, hepatic, pulmonary, renal/genitourinary, or neurologic) ≥ grade 3, which is not pre-existing and not due to the underlying condition and occurs within 30 days of cell infusion and does not resolve to grade 2 within 7 days or ≥ grade 3 clinically relevant neutropenia or thrombocytopenia lasting ≥ 28 days from the time of infusion) *or inadequate response (less than 4 responding subjects).*<br>*Stage 2:* 16 subjects will be accrued with stopping rules for relevant toxicity as defined by more than 6 subjects experiencing relevant toxicities as described above.<br>*Power calculations were performed with R (Version 4.0.1) software.*

2 *ASSESSMENT SCHEDULE*

All visits marked with an asterisk (*) must also be attended by an Investigator from Med 3, as disease-specific examinations are carried out and scores are collected. At all other visits, the presence of an Investigator from Med 5 is sufficient.

	Screening (outpatient)	Leuka-pheresis (inpatient)	Lympho-depletion (inpatient)	Treatment (inpatient)				Short-term Follow-up (inpatient at least until D10, afterwards outpatient)						Mid-term FU (outpatient)			
Study Day / Week	D-45 to D-14	D-13	D-6 to D-3	D0				D1	D2	D7	D10	D14	D28	W8	W12	W16	W24
Time Frame	±1day	±1day	±1day								±1day	±2days	±2days	±4days	±4days	±4days	±4days
Visit	V1*	V2	V3*	V4				V5	V6	V7	V8	V9*	V10*	V11*	V12*	V13	V14*
Time Point				0h*	1h	2h	4h										
**Assessments**																	
Informed Consent[1]	✓																
Inclusion & exclusion criteria	✓		✓[2]	✓													
Demography	✓																
Medical History	✓																
Prior/Concomitant Medication	✓	✓	✓	✓	✓	✓	✓	✓	✓	✓	✓	✓	✓	✓	✓	✓	✓
Physical examination	✓	✓	✓	✓				✓	✓	✓	✓	✓	✓	✓	✓	✓	✓
Vital signs[3]	✓	✓	✓	✓	✓	✓	✓	✓	✓	✓	✓	✓	✓	✓	✓	✓	✓
Brain MRI	✓																
**Leukapheresis**		✓															
Chest X-ray (to check position of CVC)			✓														
**IMP Administration**				✓													
12-lead ECG + Echocardiography	✓																

[1] Investigators of both, M3 and M5 required
[2] Only once prior to lymphodepletion procedure
[3] Vital signs: blood pressure, heart rate and body temperature

	Screening (outpatient)	Leuka-pheresis (inpatient)	Lympho-depletion (inpatient)	Treatment (inpatient)				Short-term Follow-up (inpatient at least until D10, afterwards outpatient)						Mid-term FU (outpatient)			
Study Day / Week	D-45 to D-14	D-13	D-6 to D-3	D0				D1	D2	D7	D10	D14	D28	W8	W12	W16	W24
Time Frame	±1day	±1day	±1day								±1day	±2days	±2days	±4days	±4days	±4days	±4days
Visit	V1*	V2	V3*	V4				V5	V6	V7	V8	V9*	V10*	V11*	V12*	V13	V14*
Time Point				0h*	1h	2h	4h										
**Assessments**																	
Continuous ECG				✓													
Screening laboratory tests[4]	✓																
Serum antibody levels[5]	✓																✓
Test for SARS-CoV-2 infection[6]		✓															
Pregnancy test	✓		✓[7]														
Fertility protection and consultation	✓																
Hematology[8]	✓	✓	✓🖷	✓				✓	✓	✓	✓	✓	✓	✓	✓	✓	✓
Lymphocyte subset analysis	✓			✓						✓			✓		✓		✓
Clinical chemistry	✓[9]	✓	✓🖷 / 9 / 10	✓📼				✓	✓	✓📼	✓	✓	✓📼	✓	✓📼	✓	✓📼
Coagulation (PTT, INR)	✓	✓	✓🖷	✓[11]				✓	✓[11]	✓[11]	✓	✓	✓	✓	✓	✓	✓
Immunology	✓			✓						✓		✓	✓	✓	✓	✓	✓
ESR	✓			✓						✓		✓	✓	✓	✓	✓	✓
Urine Analysis	✓		✓🖷	✓						✓		✓	✓	✓	✓	✓	✓

[4] Laboratory tests performed at screening visit: TBC, hepatitis B, hepatitis C, HIV 1/2, TSH basal
[5] Serum antibody levels for tetanus, measles, SARS-CoV-2 and EBV
[6] SARS-CoV-2 PCR test is performed as part of the inpatient admission for visit 2
[7] Pregnancy test and urine analysis only once prior to lymphodepletion procedure; Hematology, clinical chemistry and coagulation prior to lymphodepletion procedure and on Day -3
[8] Hematology: FBC with differential white blood cell count
[9] Clinical chemistry: additional (extended level) parameters on indicated visits
[10] eGFR: will be done in subjects with moderate impairment of renal function daily during lymphodepleting chemotherapy
[11] Coagulation additional parameters: Fibrinogen, Antithrombin III, D-Dimers

	Screening (outpatient)	Leuka-pheresis (inpatient)	Lympho-depletion (inpatient)	Treatment (inpatient)				Short-term Follow-up (inpatient at least until D10, afterwards outpatient)						Mid-term FU (outpatient)			
Study Day / Week	D-45 to D-14	D-13	D-6 to D-3	D0				D1	D2	D7	D10	D14	D28	W8	W12	W16	W24
Time Frame	±1day	±1day	±1day								±1day	±2days	±2days	±4days	±4days	±4days	±4days
Visit	V1*	V2	V3*	V4				V5	V6	V7	V8	V9*	V10*	V11*	V12*	V13	V14*
Time Point				0h*	1h	2h	4h										
**Assessments**																	
Serum IL6, procalcitonin, Ferritin				✓				✓	✓	✓	✓	✓	✓				
Ig Serum levels[12]			✓[13]										✓	✓	✓	✓	✓
CMV-DNA Beta-D-Glukan				✓						✓							
Adverse Events	✓	✓	✓	✓	✓	✓	✓	✓	✓	✓	✓	✓	✓	✓	✓	✓	✓
Neurological Assessments[14]				✓				✓	✓	✓	✓	✓	✓		✓		
PtGA and PhGA of disease activity	✓			✓						✓			✓	✓	✓		✓
HAQ-DI	✓			✓						✓			✓	✓	✓		✓
FACIT Fatigue	✓			✓						✓			✓	✓	✓		✓
QLQ-C30	✓			✓						✓			✓	✓	✓		✓
Disease specific PROs and composite scores[15]	✓			✓						✓			✓	✓	✓		✓
Lung function[16]	✓																✓
CT Thorax[17]	✓																✓

[12] Serum levels of IgG (+subclasses), IgA and IgM
[13] Only once prior to lymphodepletion procedure
[14] Neurological assessments will be done at the indicated timepoints and daily in all subjects with clinical signs of neurotoxicity
[15] Disease-specific assessments: ***SLE***: BILAG, SLEDAI; ***SSc***: mRSS; ***DPM***: MMT, extramuscular activity; underlined assessments required for study eligibility on screening visit
[16] Lung function: Forced Vital Capacity (FVC), Total Lung Capacity (TLC) and DLCO (diffusion capacity). At Screening in all subjects, at visit 14 / week 24 only in SSc and DPM with pulmonary involvement
[17] CT Thorax: only in SSc. If HR-CT has been done during the last 6 months as part of clinical routine, no baseline investigation will be necessary

	Screening (outpatient)	Leuka-pheresis (inpatient)	Lympho-depletion (inpatient)	Treatment (inpatient)				Short-term Follow-up (inpatient at least until D10, afterwards outpatient)						Mid-term FU (outpatient)			
Study Day / Week	D-45 to D-14	D-13	D-6 to D-3	D0				D1	D2	D7	D10	D14	D28	W8	W12	W16	W24
Time Frame	±1day	±1day	±1day								±1day	±2days	±2days	±4days	±4days	±4days	±4days
Visit	V1*	V2	V3*	V4				V5	V6	V7	V8	V9*	V10*	V11*	V12*	V13	V14*
Time Point				0h*	1h	2h	4h										
**Assessments**																	
complementary research Med 5	✓[18]		✓	✓					✓	✓	✓	✓	✓	✓	✓		✓
complementary research Med 3	✓		✓										✓	✓	✓		✓
Exploratory biomaterial[19]	✓																✓
Physical function[20]	✓	✓											✓		✓		✓

[18] Complementary research Med 5 blood will be collected on last day of screening i.e., on the day of the inpatient admission before apheresis (D -14)
[19] Exploratory biomaterial: stool probe (microbiome)
[20] Physical function: ***SLE:*** general physical activity (accelerometry): Wearable is issued only on V1 – Screening (not on V2 – Leukapheresis) and worn according to protocol; ***SSc:*** hand function; ***DPM:*** 2-minute walk test

3 *STUDY ADMINISTRATIVE STRUCTURE*

**Sponsor**	*Universitätsklinikum Erlangen*<br>*represented by the Dean Professor Dr. med. Markus F. Neurath*<br>*Medizinischen Fakultät*<br>*Friedrich-Alexander-Universität Erlangen-Nürnberg*<br>*Maximiliansplatz 2*<br>*91054 Erlangen*<br>*Tel.: 09131/85-47047*<br>*Fax: 09131/85-35120*
**Sponsor designee**	*Professor Dr. med. Georg Schett*<br>*Department of Internal Medicine (Med 3)*<br>*Universitätsklinikum Erlangen*<br>*Ulmenweg 18*<br>*91054 Erlangen*<br>*Tel.: 09131/85-32093*<br>*Fax: 09131/85-35784*<br>*E-Mail: georg.schett@uk-erlangen.de*
**Site principle Investigator**	*Professor Dr. med. Georg Schett*<br>*Department of Internal Medicine (Med 3)*<br>*Universitätsklinikum Erlangen*<br>*Ulmenweg 18*<br>*91054 Erlangen*<br>*Tel.: 09131/85-32093*<br>*Fax: 09131/85-35784*<br>*E-Mail: georg.schett@uk-erlangen.de*
**Site contact Dept. of Hematology/ Oncology**	*PD Dr. med. habil. Fabian Müller*<br>*Department of* Hematology/ Oncology (Med 5)<br>*Universitätsklinikum Erlangen*<br>*Ulmenweg 18*<br>*91054 Erlangen*<br>*Tel.: 09131/85-43107*<br>*E-Mail: fabian.mueller@uk-erlangen.de*
**Manufacturer of ATMP**	*Dr. rer. nat. Michael Aigner*<br>*Department of* Hematology and Internal Oncology (Med 5)<br>*Universitätsklinikum Erlangen*<br>*Ulmenweg 18*<br>*91054 Erlangen*<br>*Tel.: 09131/85-43160*<br>*E-Mail: michael.aigner@uk-erlangen.de*
**Contract Research Organization (CRO)**	Universitätsklinikum Erlangen<br>Center for Clinical Studies (CCS Erlangen)<br>Krankenhausstraße 12<br>91054 Erlangen<br>Tel.: 09131/85-47023<br>Fax: 09131/85-35120<br>E-Mail: info.ccs@uk-erlangen.de

**Statistician**	*Dr. Koray Tascilar*<br>*Department of Internal Medicine (Med 3)*<br>*Universitätsklinikum Erlangen*<br>*Ulmenweg 18*<br>*91054 Erlangen*<br>*Tel.: 09131/85-36924*<br>*Fax: 09131/85-35784*<br>*E-Mail: koray.tascilar@uk-erlangen.de*

4 BACKGROUND INFORMATION

4.1 Investigational product

4.1.1 Background information on the Investigational Product

The investigational medicinal product (IMP) "**MB-CART19.1**" consists of autologous CD19 Chimeric Antigen Receptor (CAR) transduced CD4/CD8 enriched T cells, derived from a leukapheresis product and processed by using the CliniMACS Prodigy® device. MB-CART19.1 is a suspension of fresh CD4/CD8-enriched CD3+ T cells that have been gene-modified with a self-inactivating (SIN) lentiviral vector expressing a CAR directed against human CD19. The SIN lentiviral vector is derived from clinically validated viral vectors described in the literature with a fully characterized sequence ([1], [2], [3]).

MB-CART19.1 will be manufactured in one certified manufacturing site (GMP laboratory of the Universitätsklinikum Erlangen, *Department of* Hematology and Internal Oncology; Med 5). The CliniMACS Prodigy® performs all manufacturing steps in a single automated and functionally closed system ([4]). MB-CART19.1 will be manufactured for each subject individually (personalized therapy) and is derived from one leukapheresis collection of each subject. After the apheresis and T cell enrichment the cells are activated, transduced with a lentiviral vector delivering the genetic information for the CD19 CAR and expanded. Thus, the IMP is an autologous T cell preparation potentially containing minor contaminations with other autologous defined cell types defined in the IMPD and considered acceptable. After infusion, the modified subjects' T cells will eliminate the CD19 expressing cells, including autoreactive B cells. Miltenyi Biotec provides the CliniMACS Prodigy® platform and all its components for selection, activation, transduction and amplification of T cells. MB-CART19.1 are to be applied as a single infusion in subjects with systemic autoimmune disease, such as Systemic Lupus Erythematosus (SLE), Systemic Sclerosis (SSc) and Dermatomyositis (DM).

4.1.2 Role of B cells in systemic autoimmune diseases

B cells have become more appreciated as major players and also as therapeutic targets in systemic autoimmune diseases in recent years. In healthy individuals, a large fraction of immature B cells is autoreactive, but autoreactive cells producing potentially harmful antibodies are progressively eliminated by central and peripheral tolerance checkpoints during the B cell maturation and selection processes. Although the mechanisms that silence autoreactive B cells

at the two checkpoints might be very different, failure to remove autoantibodies at either stage likely results in increased number of naive circulating self-reactive B cells in the periphery and, therefore, increased susceptibility to autoimmunity, as it has been hypothesized in autoimmune systemic diseases. The pathogenic roles for B cells in these diseases include autoantibody dependent and independent functions whose importance may vary across diseases or even in subsets of patients with the same disease. Even though clinical presentation and organ involvement may be different, these diseases share common pathways of B cell activation. Autoantibodies and autoantibody-producing B cells have been identified as key drivers of disease, and B cell targeting by the monoclonal antibody rituximab, targeting the surface molecule CD20 on B cells, has been widely used to treat systemic autoimmune diseases. While efficacy of rituximab is not complete and a considerable proportion of patients with systemic autoimmune diseases fail to respond to rituximab, it is still used and plays a key role in the treatment of SLE ([5]), SSc ([6]) and DM/PM ([7]). Antibodies targeting CD19 (such as tafasitamab) have been recently developed for the treatment of B cell lymphoma and leukemia, but have not been used in autoimmune disease to date ([8]).

Of note rituximab does not deplete B cells in the tissues well and thus a considerable number, if not the majority, of B cells escape depletion, making a reset of the immune alterations observed in systemic autoimmune diseases unlikely. Thus, the “inaccessibility” and persistence of autoreactive B cells, residing within lymphatic organs and inflamed tissues, limits the efficacy of B cell depletion via CD20 targeting antibodies ([9], [10]). In addition, another concern is related to the fact that CD20 is not expressed by plasmablasts and long-lived plasma cells, which are involved in autoantibody formation in systemic autoimmune diseases, and escape their depletion by rituximab. In contrast, CD19 is also expressed on plasmablasts and could thus bear the advantage to better target autoantibody producing cells. Nonetheless depth of B cells depletion appears to be a crucial factor also with CD19 targeted approaches as reports have shown that CD19 CAR T cell therapy can be effective in patients with B cell malignancies that previously failed anti-CD19 antibodies ([11]).

4.1.3 Indication

Systemic autoimmune diseases, such as Systemic Lupus Erythematosus (SLE), Systemic Sclerosis (SSc), and Dermatomyositis/Polymyositis (DM/PM) are among the most life-threatening diseases. Despite the substantial advances in their management, some patients with systemic

autoimmune diseases fail to respond to the current state-of-the art therapies, and are at high risk for severe organ failure and even death. Therefore, new treatments for such refractory cases are urgently needed. CASTLE is a basket study on three prototypic systemic autoimmune diseases, which (a.) show a severe life-threatening disease course, (b.) are associated with a current high unmet need for treatment, (c.) show an involvement of B lymphocytes in disease pathogenesis and (d.) respond at least partially to treatment with B cell depleting antibodies.

Table 1: Characterization of the three diseases indicated for the study treatment.

	**Systemic Lupus Erythematosus**	**Systemic Sclerosis**	**Dermato-myositis/ Polymyositis**
Autoantibodies	dsDNA, Sm, Ro52, Histones, Nucleosomes	e.g. Centromers, Scl 70,75,100, NOR90, RP	e.g. Mi2, Tif1, MDA5, Jo1, NXP2
Current Therapies	Glucocorticoids, MMF, HCQ, CYC, RTX	Glucocorticoids, MMF, CYC, MTX, Nintedanib, Tocilizumab	Glucocorticoids, MMF, CYC, RTX
Medical need	Clinical remission often not achievable, limited treatment options		

4.1.3.1 Systemic Lupus Erythematosus (SLE)

SLE is a systemic autoimmune disease with a prevalence of 0.1% that predominantly affects young women ([12]). SLE is characterized by a break of B cell tolerance and the emergence of autoantibodies directed against double-stranded (ds) DNA, and other nuclear antigens, which subsequently trigger immune complex-induced inflammation in an array of different organs, such as the kidneys, the brain, the joints and the skin. SLE can also affect the hematopoietic system with autoimmune-induced cytopenia and can affect the vessels leading to anti-phospholipid antibody-triggered thrombotic events, endocarditis or neurological symptoms (headache and organic encephalopathy). Persistent disease activity is associated with cumulative organ damage and premature mortality ([13]). Furthermore, like with any other systemic autoimmune disease, there is no strategy for cure of SLE, which would allow a “reset” of the immune system, and patients therefore usually require life-long medical treatment.

4.1.3.2 Systemic sclerosis (SSc)

SSc is a systemic autoimmune disease with a prevalence of 0.1% that predominantly affects the skin and the lungs. From a clinical point of view, SSc is one of the most severe systemic rheumatic diseases carrying an increased mortality rate. ACR/EULAR classification criteria were set in 2013, which were designed to allow diagnosis of early disease prior to the development of overt fibrotic manifestations ([14]). SSc is characterized by vasculopathy and a break in B cell tolerance leading

to autoantibodies (e.g. against SCL70) that precede the onset of organ fibrosis. Fibrotic manifestations predominantly affect the skin and the lungs. Therapeutic options are rather limited and modestly effective to date, resulting in a high morbidity and mortality in SSc ([15]). Dysregulation of B cell homeostasis and infiltration of fibrotic tissue by B cells has been demonstrated in SSc, which together with robust autoantibody formation indicates B cells being as key driver of SSc ([16]). B cell depleting therapy using rituximab has shown beneficial effects on the progression of skin fibrosis in a phase 2/3 study ([17]).

4.1.3.3 Dermatomyositis/Polymyositis (DM/PM)

Adult dermatomyositis/polymyositis (DM/PM) is a group of rare (prevalence 1/100.000) autoimmune diseases that involve the skin, the striated muscle (predominately in proximal muscles, leading to progressive muscle weakness) and the lungs (severe interstitial lungs disease). DM/PM is characterized by a break in B cell tolerance towards several intracellular proteins, among them RNA synthetases (anti-synthetase syndrome; e.g. anti-Jo-1 antibodies), factors involved in gene transcription (Mi-2, NXP-2) and anti-viral immunity (MDA-5) ([18]). Some forms of DM/PM (e.g. MDA-5) are associated with severe progressive interstitial lung disease with high therapeutic resistance and premature death. To date, treatment of DM is confined to broad-spectrum immunosuppressive drugs as well as to B cell depleting agents, which show partial efficacy in DM but do often not completely halt disease progression.

4.2 Preliminary data

4.2.1 Preclinical models

A strong preclinical rationale supports the use of anti-CD19 CAR T cells in systemic autoimmune disease. Experimental animal models back the concept that anti-CD19 CAR T cell therapy could be a powerful approach to target autoimmunity and inflammation in B cell mediated autoimmune disease. The only good models for systemic autoimmunity exist for SLE, which also reflect the role of the autoimmune part, i.e. the B cell influence on the disease. Hence, one study by Kansal *et al*. showed that anti-CD19 CAR T cell therapy improves the NZB × NZW F_1 model as well as in the MRL-lpr models of SLE ([19]). Both models base on the development of systemic autoimmunity reflected by anti-nuclear and anti-double stranded DNA antibody formation and the development of nephritis and premature death. $CD8^+$ T cells expressing CD19-targeted chimeric antigen receptors (CARs) persistently depleted $CD19^+$ B cells, eliminated autoantibody

production and also reversed glomerulonephritis and other disease manifestations in both experimental models. CAR T cells were found over 1 year in vivo. Of note, adoptively transferred T cells from the spleens of CAR T cell–treated mice also depleted CD19$^+$ B cells and reduced disease in naive autoimmune mice, indicating that disease control was cell-mediated. In a second study, Jin et al. also showed that anti-CD19 CAR T cells are effectively controlling autoantibody production and inhibiting glomerulonephritis in the MRL-lpr mouse model of SLE ([20]). They showed that anti-CD19 CAR T cells lead to a more sustained B cell-depletion than antibody treatment. Anti-CD19 CAR T cells did not only prevent disease before the onset of symptoms but also displayed therapeutic benefits at a later stage after disease progression. Furthermore, the authors showed that a 4-1BB co-stimulatory motif had better therapeutic efficiency than a CD28 co-stimulatory motif. These two models impressively support the role of anti-CD19 CAR T cell therapy in systemic autoimmune disease. Of note, disease pathogenesis with T cell mediated B cell activation, autoantibody production and autoantibody mediated tissue damage is very similar in SLE, SSc and DM. This is also supported by the aforementioned clinical efficacy of B cell depleting antibodies used in all three diseases.

Established preclinical models to test anti-CD19 CAR T cells only exist in SLE. For myositis several preclinical models have been published but none of them has been sufficiently standardized ([21]). Published models of myositis are poorly reproducible, methodological complex and do not reflect the pathophysiology of human myositis. For instance, many models base on infections or use species other than mice or reflect inclusion body myositis, which is not based on adaptive immune system pathology. Most importantly, none of the models reflect T cell- and B cell activation associated with myositis-specific autoantibody formation. The only model of myositis associated with ANA (but not disease-specific antibody) formation has been published 20 years ago and has virtually not been used since then ([22]). Hence, no suitable models to address the role of B cells in myositis exist to date.

While experimental models for fibrosis exist, they reflect the common effector phase of fibrosis but not B cell mediated autoimmunity ([23]). The bleomycine-models of fibrosis leads to inflammation followed by fibrosis after exposure to the cytostatic agent bleomycin. The model is not dependent on T cell/B cell activation and does not develop disease-specific autoantibodies. The tight skin (Tsk) mouse models of skin fibrosis (Tsk-1 and Tsk-2) are based on a mutated fibrillin-1 protein that is deposited in the extracellular space. These models do not show inflammatory infiltrates and do not lead to accumulation of T cells or B cells in the affected skin ([24]). Furthermore, autoantibodies are inconsistently found in this model, mostly against the

mutated fibrillin. Anti-nuclear antibodies are absent. Due to lack of inflammation, which is a hallmark of human SSc, these models are considered to reflect more the tight skin disease in humans than SSc. In the absence of an adequate target (B cells) in the skin these models are not useful to study the effects of CD19-targeted CAR T cell therapy in SSc. Furthermore, the murine sclerodermatous chronic graft versus host disease model, which is based on transplantation of splenocytes of LP/J (H-2^b) mice into irradiated C57/Bl6 (H-2^b) mice with a minor HLA mismatch does not involve B cells and does also not lead to the production of autoantibodies associated with systemic sclerosis in humans. Hence, no suitable models to address the role of B cells in myositis exist to date.

In summary, no suitable mouse models for myositis and systemic sclerosis exist to date that would allow to study the role of CD19 CAR T cells in respective human diseases in a reliable or meaningful way. In contrast, ample clinical evidence suggests that all three diseases are sensitive to B cells depletion highlighting their common pathophysiology with B cell mediated autoantibody production as central pathophysiologic event.

4.2.2 Clinical data

Our current experience on the treatment of life-threatening autoimmune disease with MB-CART19.1 extends to SLE, DM/PM and SSc. To date, 7 patients with life-threatening and treatment resistant SLE, 3 patients with life-threatening and treatment resistant SSc and 2 patients with life-threatening and treatment resistant DM/PM have been successfully treated with MB-CART19.1 (Table 2) ([25], [26], [27], [28]). All patients failed on multiple standard treatments including pulsed steroids, hydroxychloroquine, mycophenolate mofetil, cyclophosphamide, tacrolimus, intravenous immunoglobulins, rituximab and belimumab and had severe organ involvement of the kidneys, the lungs and the heart.

After MB-CART 19.1 administration, CAR T cells showed a consistent expansion during the first 9 days with maximal circulating cell numbers ranging from 25 CAR T cells/microliter to 2.477 CAR T cells/microliter. Thereafter, circulating CAR T cells rapidly decreased potentially populating the tissue niches. B cells were completely absent within the peripheral blood from day 2 after CAR T cells administration. Safety of CAR T cell treatment is of paramount importance. None of the patients showed higher grade (grade 3 or 4) Cytokine Release Syndrome (CRS) or Immune-effector Cell Associated Neurotoxicity Syndrome (ICANS). Even, no grade 2 CRS or ICANS was observed. Some, but not all patients showed very mild signs of CRS (grade 1: mild fever) and one patient had very mild signs of ICANS (grade 1: mild reversible ataxia). No other side effects, i.e.

no infections occurred. Also, no prolonged (> 10 days) neutropenia and thrombocytopenia occurred. With respect to efficacy, all patients showed high-level clinical responses with DORIS remission of SLE, ACR Major Response of DM/PM and improvement of manifestations of SSc with healing of digital ulcers, improvement of Raynaud´s phenomenon and no worsening of lung function. Furthermore, all patients could successfully stop their immune suppressive treatment including glucocorticoids. No relapses occurred with follow-up periods up to 23 months after CAR T cell therapy. Patients with sufficiently long follow-up time also seroconverted showing loss of autoantibodies against double-stranded DNA (dsDNA) in SLE, loss of autoantibodies against Jo-1 in DM/PM and loss of autoantibodies against RP11 in SSc. Furthermore, all patients with sufficient long follow-up time (10/12) showed reconstitution of B cells between 52 and 205 days after CAR T cell therapy. Despite B cell reconstitution, autoimmune disease did not recur in any of the patients.

Taken together, these data, though limited in its number, provide a highly consistent picture of MB-CART19.1 therapy in SLE, DM/PM and SSc suggesting very good safety (no CRS > 1; no ICANS > 1) and efficacy (drug-free remission including stop of glucocorticoids or improvement in case of SSc) among the three autoimmune diseases.

	Disease	Sex	Age	Months post CAR T cell therapy	B cell aplasia (days)/ B cell reconstitution	CRS	ICANS	Drug -Free State	Sero-Conversion	Clinical Response
**1**	SLE	F	22	23	148/ Yes	0	0	Yes	Yes	DORIS Remission
**2**	SLE	M	23	18	136/ Yes	1	0	Yes	Yes	DORIS Remission
**3**	SLE	F	23	15	120/ Yes	0	0	Yes	Yes	DORIS Remission
**4**	SLE	F	25	15	93/ Yes	1	0	Yes	Yes	DORIS Remission
**5**	SLE	W	19	13	52/ Yes	0	0	Yes	Yes	DORIS Remission
**6**	SLE	F	39	10	205/ Yes	1	0	Yes	Yes	DORIS

										Remission
**7**	SLE	F	33	6	58/ Yes	0	0	Yes	Yes	DORIS Remission
**8**	DM/PM	M	42	10	128/ Yes	1	0	Yes	Yes	ACR Major Response
**9**	DM/PM	F	43	5	93/ Yes	1	1	Yes	Not Yet*	ACR Major Response
**10**	SSc	M	60	9	77/ Yes	1	0	Yes	Yes	Improvement**
**11**	SSc	M	36	3	-/ Not Yet*	0	0	Yes	Not Yet*	Improvement**
**12**	SSc	M	36	2	-/ Not Yet*	1	0	Yes	Not Yet*	Improvement**

Table 2: Current status of CAR T cell treatment in autoimmune disease with safety and efficacy data.

ACR: American Colleague of Rheumatology; CRS: Cytokine Release Syndrome; DM/PM: Dermatomyositis/Polymyositis; DORIS: Definitions of Remission in SLE; ICANS: Immune effector cell-associated neurotoxicity syndrome; SLE: Systemic Lupus Erythematosus; SSc: Systemic Sclerosis; (*) too short follow up to observe B cell reconstitution or seroconversion. (**) Improvement of Raynaud's phenomenon and digital ulcers with no worsening lung function.

4.3 Rationale

SLE, DM/PM and SSc are usually treated with glucocorticoids and traditional immune suppressive drugs such as mycophenolate mofetil (MMF). Most of the drugs are not approved for their use in SLE, myositis and systemic sclerosis. In addition, in SLE hydroxychloroquine and the monoclonal antibody belimumab are used, in DM/PM occasionally intravenous immunoglobulins are used, while in systemic sclerosis the tyrosine kinase inhibitor nintedanib is approved for interstitial lung disease including interstitial lung disease in conjunction with systemic sclerosis.

Guidelines for treatment of DM/PM ([29]) suggest to use glucocorticoids before using traditional immune suppressive drugs or intravenous immunoglobulins. None of these agents is approved in DM/PM. Failure on glucocorticoids and two or more immune suppressive drugs is considered as a resistant case with overall poor prognosis and low chance to get in remission.

Guidelines for the treatment of SSc ([30], [31]) are based on the respective organ involvement and contain mostly supportive and symptomatic measures. Very few immunosuppressive drugs have shown efficacy in SSc and none of them are approved. There is currently no guideline how to treat SSc with high risk for progression, although most often MMF is used. MMF shows limited

effects in SSc. In case of progressive lung fibrosis, which is not the patient population included in this study, rather toxic regimens like repeated cyclophosphamide courses and even bone marrow transplantation is suggested. Furthermore, nintedanib is used if interstitial lung disease is present. Overall, effective immune intervention in SSc are very limited to date and progressive resistant disease is associated with poor prognosis.

Guidelines for the treatment of SLE ([32], [33], [34]) also consider organ involvement. Glucocorticoids and hydroxychloroquine are widely suggested in SLE, followed by the use of MMF in case of kidney involvement. There is guideline for treatment escalation in SLE if drugs don't work and the disease progresses. Failure on glucocorticoids and two or more immune suppressive drugs is considered as a resistant case with overall poor prognosis and low chance to get in remission.

Sizeable numbers of patients do not respond to these treatments and suffer from substantial therapy-induced adverse effects, which include recurrent infections, osteoporosis or accelerated cardiovascular disease and infertility ([35]). Failure to treatment results in organ damage and premature death, which is dramatic as most of the patients with systemic autoimmune diseases are of young age. Furthermore, the necessity to control disease activity requires the use of high and sustained doses of glucocorticoids leading to additional toxicity and comorbidity.

4.3.1 Rationale of the study

SLE, SSc and DM/PM share a common pathogenesis with a breach in immune tolerance against nuclear antigens leading to the emergence of anti-nuclear antibodies (ANA). This process requires the formation of autoimmune B cells that are a source for cytokine production, act as antigen presenting cells and are responsible for the synthesis of disease-associated autoantibodies. The role of B cells in SLE, SSc and DM/PM is illustrated by the fact that all three autoimmune diseases are sensitive to B cell depleting therapy with the CD20-targeted antibody rituximab ([5-7]). Furthermore, immunohistochemistry studies have demonstrated that B cells are infiltrating the inflammatory lesions of SLE, SSc and DM/PM. In SLE, for instance, B cells and plasma cells are present in the inflamed kidneys and contribute to local autoantibody production ([36, 37]). In SSc, B cells infiltrates are found in the lesional skin and are enriched in the affected lung of SSc patients ([38, 39]). In DM/PM, B cells and plasma cells are enriched in the inflammatory infiltrates in the affected muscles and are also present in the associated interstitial lung disease, where they are associated with resistance to treatment ([40, 41]). Taken together, these data consistently show that B cells and plasmablasts/plasma cells are an essential part of

the inflammatory lesions in SLE, SSc and DM/PM. Furthermore, it has been shown that conventional B cell depletion with CD20 targeted antibodies does not deplete tissue memory B cells in the secondary lymphatic tissues of patients with SLE ([42]) and kidney transplant recipients ([9]) as well as rheumatoid arthritis synovial tissue ([43]), indicating that antibody-based B cell targeting is not efficient in eradicating the autoimmune B cell clones in the tissues. Therefore, deep targeting of B cells could provide a tool to eliminate B cells and autoimmune B cells clones from the tissues and induce drug-free remission of disease.

Our current experience (see 4.2.2) on the treatment of SLE, DM/PM and SSc with MB-CART19.1 support this concept and suggest that CAR T cell mediated depletion of B cells is feasible, safe and efficacious in these B cell mediated autoimmune diseases. We observe that disease activity successively decreases during the B cell aplasia phase in the first 3 months after CAR T cell administration. These effects occur despite the complete cessation of all immune suppressive treatment including glucocorticoids. Moreover, we observe the disappearance of disease specific autoantibodies including dsDNA autoantibodies in SLE, Jo-1 autoantibodies in DM/PM and RP11 in SSc indicating that autoimmune B cells clones were tackled by this treatment. In addition, we observe ongoing remission of autoimmune diseases, drug-free state and absence of autoantibodies despite reconstitution of B cells 4-5 months after CAR T cell treatment indicating that immune reconstitution is not associated with recurrence of disease in these patients. Analysis of the recurring B cells have suggested a thorough reboot of the B cell system with abrogation of memory B cells and plasmablasts and the emergence of a naïve non-calls switched B cells system. Furthermore, long-term vaccination responses remained untouched by CAR T cell treatment indicating that these responses de-rive from long-lived plasma cells, which do not express CD19 on their surface and therefore not touched by CAR T cells. Based on these data, we think that CAR T cell treatment has a high impact on patients with SLE, DM/PM and SSc as it allows to (i) abrogate autoimmunity by completely depleting autoimmune B cells clones and rebooting the B cells system, (ii) reach deep remission of disease with resolution of B cell mediated inflammation, removal of autoantibodies and protection from consecutive organ damage and (iii) yield sustained clinical effects with absence of disease despite B cell reconstitution.

Based on these data, the rationale of CD19-targeted CAR T cell therapy in autoimmune dis-ease is to induce persistent disease remission and restore humoral immune tolerance by deep and sustained elimination of autoreactive memory B cells.

4.3.2 Study design

The project is a prospective, open-label, single-dose, non-randomized, interventional phase I/II clinical study, using a basket design, to assess the safety, feasibility, and preliminary efficacy of *ex vivo* generated autologous anti-CD19 CAR T cells targeting a common pathogenic pathway (activated B cells and plasmablasts) in subjects with severe, treatment-failure systemic autoimmune disease.

4.3.3 Selection of dose and dose regimen

Subjects will receive a single intravenous infusion of 1x 10^6 /kg body weight CAR T cell by trained investigators following international quality standards (as performed by our certified CAR T cell center). The consortium had meetings with Paul-Ehrlich-Institute (PEI) to discuss this specific study in June 2021 and February 2022 including the rationale to go for a fixed MB-CART19.1 dose. Proceeding with a fixed dose of 1x 10^6 /kg body weight CAR T cell was considered as the best strategy based on (i) the good safety and efficacy observed in the first 5 patients with autoimmune disease treated with 1x 10^6 /kg body weight MB-CART19.1 and (ii) the lack of a dose response-related safety signal between 0.5 x 10^6 and 3 x 10^6 /kg body weight MB-CART19.1 administered in the lymphoma studies. As toxicities of MB-CART19.1, like any other CD19- CAR T cell therapy, is not related to the number of CAR T cells infused but the number of target B cells in the body, much less toxicities are expected in autoimmune disease considering that total B cell numbers in the body are folds lower than in B cell malignancies. This situation attributes to all three autoimmune diseases investigated in this study.

4.3.4 Lymphodepleting chemotherapy

Lymphodepleting pretreatment was shown to substantially favor *in vivo* expansion of adoptively transferred tumor-antigen specific T cells ([44], [45]) and was used in all previous successful clinical CAR T cell studies ([46], [47], [48], [49], [50], [51]). Based on the available data, all subjects will receive in vivo T cell depletion with cyclophosphamide at 1000 mg/m^2 on day -3 and fludarabine at 25 mg/m^2 on days -5 to -3 prior to T cell infusion in order to enhance expansion of adoptively transferred CD19 CAR T cells.

4.3.5 Subject population

The study will recruit subjects with severe, treatment-failure systemic autoimmune disease, i.e. Systemic Lupus Erythematosus (SLE), Systemic Sclerosis (SSc) and Dermatomyositis/Polymyositis (DM/PM).

4.4 Risk-benefit assessment

4.4.1 Assumed benefits for the subjects

CAR T cells have been genetically engineered to express CAR molecules directed against antigens expressed on malignant cells. As such, CAR T cells have heralded a new era in cancer immunotherapy ([52]). CAR T cells targeting the B cell surface antigen CD19 are already successfully used against B cell-derived leukemia and lymphoma ([53]). In accordance with the properties of endogenous cytotoxic T cells and in contrast to conventional therapeutic antibodies, CAR T cells can achieve a sustained and deep depletion of B cells within the affected tissues. Moreover, CAR T cells directly eradicate target cells, whereas monoclonal antibodies rely on antibody-dependent cellular toxicity, a mechanism that is often blunted in patients with systemic autoimmune disease.

By repurposing anti-CD19 CAR T cells to treat refractory subjects, we aim to induce long-lasting and drug-free remission in systemic autoimmune disease. Our approach could lead to restoration of a sustained immune tolerance, which might shift the current therapeutic paradigms for SLE and potentially other B cell-driven autoimmune disorders such as SSc and DM. While emphasis of this early-phase study is on feasibility and safety, we expect a substantial therapeutic benefit for a proportion of the treated subjects. Beyond the possibility that patients with life-threatening B cell-driven autoimmune disorders benefit from such treatments, this study will also promote the development of gene-engineered T cell therapies in inflammation medicine in general.

Patients with SLE, DM/PM and SSc that show a progressive disease course despite treatment with glucocorticoids and two or more immunosuppressive drugs are critically ill and have an overall poor prognosis with respect to maintenance of organ function and their quality of life. Lung and heart involvement are life threating complications in these diseases that lead to premature death. Also, drug toxicities with continuous glucocorticoid intake and cycling through several immunosuppressive drugs are substantial. Hence, CAR T cell treatment offers such patients a huge benefit and the chance to (i) abrogate the disease, (ii) stop the drugs and (iii) face long-term remission. This attributes to all three diseases in the basket.

4.4.2 Anticipated risks for the subjects

4.4.2.1 Risks due to study-related procedures

- Study-related routine procedures such as blood, leukapheresis and radiological imaging procedures are known and local standard procedures will be applied.
- Administration of the IMP will be performed according to detailed instructions given in section 8.2.4 in order to minimize risks such as extravasation and loss of the IMP during infusion.

4.4.2.2 Risks due to lymphodepleting chemotherapy

- Spurious cytopenia due to conditioning treatments with cyclophosphamide and fludarabine has been reported after CAR T cell treatment, which is managed by standardized hygienic measures and short-term antibiotic prophylaxis.

4.4.2.3 Risks due to CAR T cell therapy

- Cytokine Release Syndrome (CRS) and Immune-effector Cell Associated Neurotoxicity Syndrome (ICANS) have been reported in cancer patients ([54]). Both adverse events can be life threating, but are becoming manageable with increasing clinical experience and timely intervention with anti- IL-6 receptor antibody, corticosteroids and anti-epileptic drugs ([54]). The interdisciplinary staffs involved in the CAR T cell program at the study site is therefore extensively trained in the management in such adverse events. The safety of Miltenyi's CAR-vector based T cells (MB-CART19) is currently addressed in two clinical trials in pediatric acute lymphatic leukemia (NCT03321123) and in CD19 positive pediatric and adults B cell malignancies (NCT03853616). Of note, some but not all of the twelve patients with autoimmune disease treated with Miltenyi's CAR-vector based T cells (MB-CART19) so far, showed very mild signs of CRS (grade 1: mild fever) and one patient had very mild signs of ICANS (grade 1: mild reversible ataxia). As the incidence of CRS/ICANS correlates with B cell mass, we think that severe CRS/ICANS are unlikely to occur in patients with systemic autoimmune diseases, as the B cell mass in such conditions is many folds lower than the ones observed in B cell malignancies. In addition, we are planning to reduce the dose at $0.5x10^6$/kg bodyweight if a CRS/ICANS > grade 2 should appear.

- Prolonged B cell depletion can result in increased risk for infections due to leukopenia and/or hypogammaglobinemia, which is managed by antibiotic prophylaxis and (if necessary) immunoglobulin substitution. We consider the risk of prolonged B cell depletion as low since our observations show that all patients with sufficient long follow-up time (10/12) to date fully reconstituted with B cells between 52 and 205 days after CAR T cell infusion. In accordance, only one of the twelve patients required spurious immunoglobulin substitutions, which was well tolerated. Even if B cell depletion would last longer in one or the other patient continuous monitoring of immunoglobulin levels and the possibility to substitute immunoglobulins would make this risk very well manageable.

4.4.3 Benefit-risk ratio

Because the subject population eligible for this study is characterized by severe and uncontrolled disease, which has already failed on standard treatment and because the twelve patients treated so far had no or only mild side effects, the benefit-risk ratio of this study is estimated to be highly positive. In addition, since CRS and ICANS are considered to be related to the B cell mass and the subsequent lysis of B cells, the likelihood of CRS/ICANS in this subject population is very low, as the B cell mass in patients with systemic autoimmune disease is low as compared to lymphoma/leukemia patients.

As mentioned above, the risk for prolonged B cell depletion after CD19 CAR T cell therapy is considered to be low in autoimmune diseases. Hence, all patients with SLE that were treated with CD19 CAR T cells fully reconstituted in B cells between 52 and 205 days after CAR T cell infusion. This observation is not completely unexpected as autoimmune patients do not show bone marrow depression like lymphoma or leukemia patients, but rather exhibit a hyperactive normal bone marrow that can reconstitute if CAR T cells are exhausted.

5 STUDY OBJECTIVES

5.1 Primary Objective

- To assess the safety of anti-CD19 CAR T cell therapy in subjects with active B-driven autoimmune disease (SLE, SSc and DM/PM).

5.1.1 Primary Endpoint

- Incidence and grading of severity (graded 0-4) of Cytokine Release Syndrome (CRS) and of CAR T cell Associated Neurotoxicity Syndrome (ICANS) within the first 4 weeks after ATMP administration.

5.2 Secondary Objectives

- To assess the clinical efficacy of anti-CD19 CAR T cell therapy in subjects with active B-driven autoimmune disease (SLE, SSc and DM/PM).
- To investigate the duration of B cell depletion after anti-CD19 CAR T cell administration.
- To investigate the duration of CAR T cell persistence after anti-CD19 CAR T cell administration.
- To investigate the changes in the levels of disease-associated serum autoantibodies.
- To investigate the in vivo cellular kinetics of MB-CART19.1, immunogenicity and feasibility of the manufacturing process.

5.2.1 Secondary endpoints

5.2.1.1 Clinical efficacy

- Overall Response Rate (ORR) at week 24 measured by specific disease activity composite indexes, each of them validated for the specific disease:
    - SLE: Fulfillment of DORIS remission criteria of SLE at week 24 ([55]).
    - SSc: No progression of interstitial lung disease with worsening of FVC1 (>10%) or worsening of FVC1 (5-10%) plus increase in respiratory symptoms or worsening of FVC1 (5-10%) plus progression of high-resolution computed tomography changes after 24 weeks ([56]).

- DM: 2016 ACR/EULAR Moderate or Major Response ([57]). No progression of interstitial lung disease with worsening of FVC1 (>10%) or worsening of FVC1 (5-10%) plus increase in respiratory symptoms or worsen-ing of FVC1 (5-10%) plus progression of high-resolution computed tomography changes after 24 weeks.

5.2.1.2 Cellular and humoral response

- Duration of persistence of CAR T cells in the peripheral blood (CAR+ CD3+ cells assessed by FACS > 0)
- Duration of B cell depletion in the peripheral blood (CD19+ B cells assessed by FACS equal to 0)
- Levels of respective serum autoantibodies at week 24 including incidence of sero-conversion (anti-dsDNA < 4U/L; ANA <1.100; all others “negative”)
    - SLE: ANA, anti-dsDNA, anti-nucleosomes, anti-Sm, anti-cardiolipin IgG, C3 C4
    - SSc: ANA, anti-SCL70, anti-RNA polymerase III, anti-topoisomerase
    - DM: ANA, anti-Mi2, anti-Tif1, anti-MDA5, anti-Jo1, anti-NXP2
- Expansion of CAR T cells in the patient over time
- Success of the manufacturing process by GMP certification of the product

5.2.1.3 Additional endpoints

- General:
    - Patient’s Global Assessment (PtGA) of disease activity (VAS 0-100mm)
    - Physician’s Global Assessment (PhGA) of disease activity (VAS 0-100mm)
    - Health Assessment Questionnaire – Disease Index (HAQ-DI)
    - Functional Assessment of Chronic Illness Therapy - Fatigue (FACIT Fatigue)
    - Core Quality of Life (EORTC QLQ-C30)
- SLE:
    - British Isles Lupus Assessment Group (BILAG) index ([58])
    - Systemic Lupus Erythematosus Disease Activity Index (SLEDAI) ([59])
    - Physical function by accelerometry
- SSc:

    - modified Rodnan Skin Score (mRSS) ([60])
    - Hand function by isometric grip strength, Moberg-Pick-Up Test, short form of the Michigan Hand Questionnaire (sMHQ)
- DM/PM:
    - Physician's Global Assessment (PhGA) of extramuscular activity
    - Manual Muscle Testing (MMT) ([61])
    - Physical function by 2-minute-walk with gait assessment

5.3 Exploratory objectives

- To analyze the changes in B cell receptor repertoire.
- To evaluate the therapy-induced alterations of B compartments (permitting to evaluate the "tolerogenic reset").

5.4 Complementary Research Program

Sample collection for Complementary Research Program is optional and will only be performed in subjects who expressively have given their consent in writing after the Investigator has informed them about the details of this procedure. Sampling and storage instructions will be provided in separate documents. The Investigator will record all samples taken in a sample log in the Investigator Site File (ISF). The samples will be transferred into a local biobank; otherwise, they will be destroyed after completion of the analyses.

The following analyses are planned:

- Serum levels of BAFF, type I interferon, IFN-gamma, IL-6, TNF-alpha
- Circulating T lymphocyte subsets
- T cell and B cell receptor sequencing
- Single cell RNA sequencing including IFN signature
- Serum antibody levels against tetanus, measles, SARS-CoV-2 and EBV
- Exploratory biomaterial: stool probe (microbiome), serum and EDTA blood

6 INVESTIGATIONAL PLAN

6.1 Overall study design

This is a prospective, open-label, single-dose, non-randomized, interventional phase I/II study to assess the safety and efficacy of anti-CD19 CAR T cell therapy in subjects with active B cell driven autoimmune disease (SLE, SSc and DM/PM).

A basket design will be followed, which attributed to the fact that SLE, SSc and DM/PM share a common pathophysiology that is characterized by the generation of autoimmune B cell clones that lead to formation of autoantibodies and tissue damage. The study thereby conceptualizes that deep depletion of B cells by CD19 CAR T cells can reset autoimmunity and eradicate the progression of severe autoimmune disease by the abrogation of autoimmune B cell clones and cessation of autoantibody production. The basket design thereby also considers the effect of peripheral B cell depletion by monoclonal antibodies has been described. Based on the shared pathophysiology and described effects of B cell depletion in these diseases there is a high chance that CD19 CAR T cell therapy will be effective in all three autoimmune diseases, SLE, DM/PM and SSc.

CASTLE study primarily aims to define safety of CD19 CAR T cell therapy in these diseases as well as collect preliminary data on efficacy. With respect to safety considerations, there is no concern that safety of CD19 CAR T cell therapy is different among the three autoimmune diseases, which supports the basket design. Thus, all three diseases are non-malignant diseases, in which the overall B cell burden is many folds lower than the one in B cell malignancies. Therefore, the risk of CRS and ICANS is expected to low and comparable in all three diseases.

Furthermore, the basket design will allow a broader group of patients with severe life-threating autoimmune diseases that have previously failed on standard treatment regimen to get exposed to a potentially highly effective treatment. Following such approach safety and preliminary efficacy data can be gained in a faster and more comprehensive way

A total of 24 subjects with B cell mediated autoimmune disease will be included in the CASTLE study, following a Bryant and Day two stages design (Figure 1):

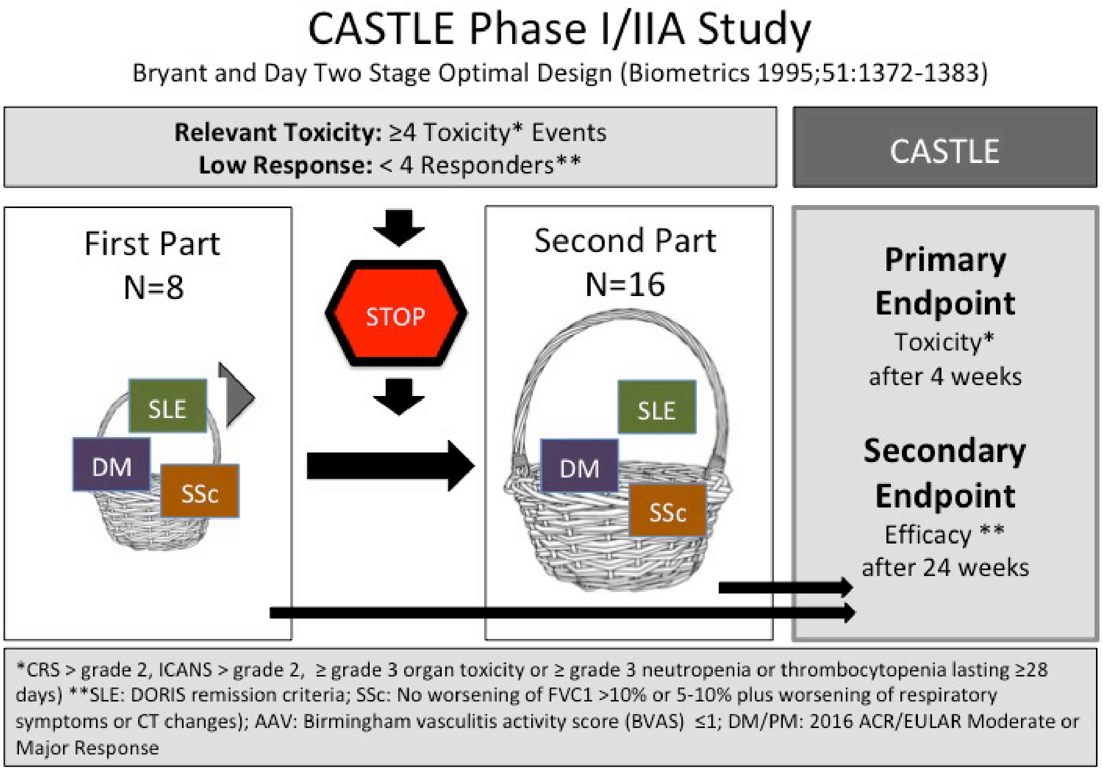

Figure 1: Overall study design.

In the first stage of the study, 8 subjects will be recruited. Safety with respect to Dose Limiting Toxicity (DLT) will be assessed (primary safety endpoint). DLT is defined as 4 or more of the following events: CRS > grade 2, ICANS > grade 2, organ toxicity (cardiac, dermatologic, gastrointestinal, hepatic, pulmonary, renal/genitourinary, or neurologic) ≥ grade 3, which is not pre-existing and not due to the underlying disease and occurs within 30 days of cell infusion and does not resolve to grade 2 within 7 days or ≥ grade 3 clinically relevant neutropenia or thrombocytopenia lasting ≥ 28 days from the time of infusion. If DLT occurs the study will be stopped. If CRS/ICANS > grade 2 occurs in 2 subjects, the dose of ATMP will be reduced from 1 to 0.5 x10^6 CAR T cells/kg body weight. If additional 2 CRS/ICANS > grade 2 occurs in the reduced dose, the study will be stopped. If no more than 3 DLTs occur and if clinical efficacy (see secondary efficacy endpoint) is observed in 4 or more subjects, the second stage (basket) will be started. In the second stage of the study, 16 additional subjects will be recruited.

Subjects' recruitment and outpatient visits will be performed in the Departments of Internal Medicine or Hematology and Internal Oncology. Treatment of subjects and the inpatient visits will be performed in the respective wards of the departments. The responsible Intensive Care Unit

(ICU) must be informed about the clinical study before inclusion of the first subject and the respective dosing date to ensure that the medical staff of the ICU ward is able to react appropriately without any timely delay in case of emergency. All structural and personnel requirements for performing the planned regular study-related investigations are given.

Subjects will be screened between day -45 and day -15. If the subject meets all of the inclusion and none of the exclusion criteria, he/she will be included in the clinical study and follow the study flow chart shown in Figure 2.

Leukapheresis of the subject will be performed at the collection site on day -13 according to local standard practice. The leukapheresis product will be transferred to the manufacturing site. The leukapheresis product will be used for the individual manufacturing of MB-CART19.1 by using the automated CliniMACS Prodigy System. The manufacturing of MB-CART19.1 will start on day -13 and will be finished on day -1 (see section 8.2).

Lymphodepleting chemotherapy will be administered between days -5 and -3. On day 5 of the manufacturing process the In-process Control (IPC) will indicate if the manufacturing process is successful. Lymphodepleting chemotherapy will not be started until the positive result of the IPC was confirmed by the manufacturer.

The day of administration of fresh MB-CART19.1 will be assigned as day 0 at the study site. The IMP must not be infused shorter than 48 hours after cyclophosphamide infusion.

During the short-term follow-up (FU), subjects will be hospitalized for at least 10 days after infusion of the IMP with close monitoring of their vital functions and lab parameters for signs of Adverse Events (AEs). Short-term FU ends with day 28 where safety and response will be assessed.

In the mid-term FU until week 24, clinical responses in all three disease cohorts will be assessed. Furthermore, Serious Adverse Events (SAEs) and Adverse Events of Special Interest (AESI) will be documented and reported.

Persistence of MB-CART19.1 in peripheral blood will be measured throughout all phases of the study after administration.

6.1.1 Study flow chart

Pre-Treatment			Treatment	Follow-Up (FU)	
**Days -45 to -15**	**Day -14**	**Days -5 to -3**	**Day 0**	**Short-term FU Days 1 to 28**	**Mid-term FU Weeks 4 to 24**
Screening	Leukapharesis	Lymphodepletion	IMP MB-CART-19.1 Infusion		
		Inpatient	*Inpatient*	*Inpatient at least until day 10*	*Outpatient*

Long-term Follow-Up
**Annualy up to 15 years**
Safety and Response Assessments

Figure 2: Study flow chart.

6.1.2 Number of subjects

A total number of 24 subjects shall be evaluated.

6.1.3 Study duration

The study is planned to start in May 2023 with respect to the first subject included (First Subject First Visit, FSFV, defined as first screening visit). Including a screening period of up to 6 weeks, the study duration per subject is maximally 30 weeks. Assuming a recruitment phase of about 30 months for enrollment of 24 subjects, the study is expected to last approximately until May 2026 (Last Subject Last Visit, LSLV).

6.1.4 Inclusion procedure

In the first stage of the study, as soon as a subject is available for screening, the Clinical Trial Manager (CTM) will coordinate dates for the screening and for leukapheresis (Day -14) with the responsible persons and institutions. Once the subject has been successfully screened and the final leukapheresis date is known, CTM will give written “green light” to proceed and enroll the subject.

Dosing of the second subject can only be performed at least 28 days after dose administration of the first subject and after written confirmation of the site principle investigator that there are no safety concerns to continue. In case of safety concerns, the interval may be increased to more than 28 days.

In the second stage of the study, enrollment will be done without timely restrictions.

6.1.5 Definition of a Dose Limiting Toxicity

Safety evaluations will be made based on the Common Terminology Criteria for Adverse Events (CTCAE Version 5.0) and according to ([62]).

Dose Limiting Toxicity (DLT) will be evaluated within 30 days after infusion of MB-CART19.1 and is defined as

- CRS > grade 2 or
- ICANS > grade 2 or
- organ toxicity (cardiac, dermatologic, gastrointestinal, hepatic, pulmonary, renal/genitourinary, or neurologic) ≥ grade 3, which is not pre-existing and not due to the underlying disease and occurs within 30 days of cell infusion and does not resolve to grade 2 within 7 days or
- ≥ grade 3 clinically relevant neutropenia or thrombocytopenia lasting ≥ 28 days from the time of infusion.

As an additional safety measure, an interval of at least 30 days between the treatment of the first and second subject is mandatory.

6.1.6 Management of toxicities

CRS with hemodynamic instability and ICANS, caused by in vivo expansion of CAR T cells, are manageable with IL-6 receptor inhibitor, corticosteroids and anti-epileptic drugs. The interdisciplinary staff involved in the project is extensively trained to treat these AEs. Moreover, incidence of CRS/ICANS correlates with tumor mass suggesting low risk in subjects with systemic autoimmune disorders. CRS and ICANS will be graded and managed according to a published consensus grading scale and corresponding treatment recommendations ([63]): Mild forms will be treated with novaminsulfone (up to 4 g/day) or alternatively by ibuprofen (up to 1800 mg/day). Moderate forms will receive additional glucocorticoids up to 1mg/kg. Severe forms will be treated by tocilizumab infusion at a dose of 8 mg/kg body weight. A more detailed tabular overview is shown in the Appendix of this protocol (14.1 to 14.4).

Any organ toxicity not otherwise explained is caused by CAR T cell administration until proven otherwise. Grading will be performed according to CTCAE (Version 5.0) with grade 5 being

defined as organ-specific side effects that lead to death. Corresponding treatment is provided according to the grade, please refer to Appendix 14.5.

A highly experienced team of hematologists will manage cytopenia and hypogammaglobinemia, AEs linked to preparatory lymphodepletion and/or CAR T cells treatment. The increased risk for infections due to leukopenia and/or hypogammaglobinemia (because of the on-target B cell depletion) will be managed by mandatory standardized antibiotic (with cotrimoxazole and ciprofloxacin in case of neutropenia) and anti-viral (aciclovir) prophylaxis in all subjects and (if necessary) immunoglobulin substitution.

6.1.7 Stopping rules

6.1.7.1 Stage 1

- 8 subjects will be accrued with stopping rules for dose-limiting toxicity (DLT) as defined by more than 4 subjects experiencing either
  - CRS > grade 2 or
  - ICANS > grade 2 or
  - organ toxicity (cardiac, dermatologic, gastrointestinal, hepatic, pulmonary, renal/genitourinary, or neurologic) ≥ grade 3, which is not pre-existing and not due to the underlying condition and occurs within 30 days of cell infusion and does not resolve to grade 2 within 7 days or
  - ≥ grade 3 clinically relevant neutropenia or thrombocytopenia lasting ≥ 28 days from the time of infusion)
- The study will be also stopped if inadequate response is observed, i.e. less than 4 subjects responding.

6.1.7.2 Stage 2

- 16 subjects will be accrued with stopping rules for relevant toxicity as defined by more than 6 subjects experiencing relevant toxicities as described above.

6.1.8 Safety Management Board

The SMB consists of a minimum of two external medical experts and a statistician. After completion of the first stage of the study, i.e. when the first eight subjects have completed the short-term FU (thus the SMP will start evaluation within the first 4 weeks after administration of the IMP in the first subject), an interim safety report will be prepared including AEs/SAEs and other safety and toxicity results. The responsibility of the SMB will be to review the clinical safety, toxicity and laboratory data in each disease cohort and to give advice to the Sponsor and the Investigators how to proceed with the study. Details are specified in the DSMB Charter.

6.1.9 End of study

The end of study will be defined as the last visit of the last subject (LSLV) after completion of mid-term follow-up until week 24.

6.2 Treatment procedure

With respect to the treatment, each individual subject recruited will undergo the following procedure: After screening consenting and fulfilment of inclusion/exclusion criteria subjects will receive a leukapheresis from the peripheral blood to obtain T cells for manufacturing the Advanced Therapy Medicinal Product (ATMP) from autologous T cells. During the manufacturing, subjects will receive lymphodepletion before the administration of the freshly produced ATMP, consisting of $1x10^6$/kg body weight CAR T cell targeting CD19 B cells (Figure 3).

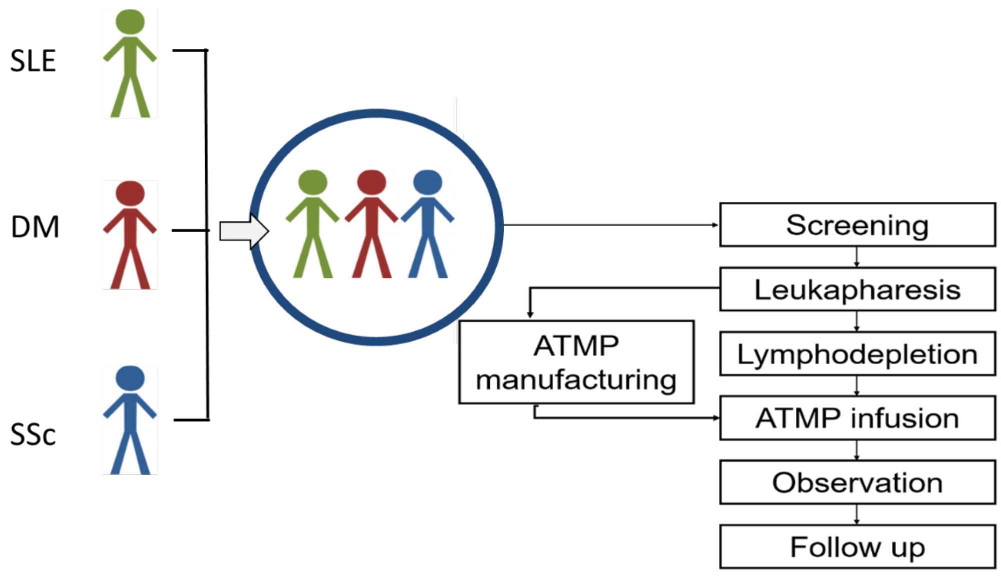

Figure 3: Schematic overview of study procedure.

6.2.1 Screening (Visit 1)

The subject will be informed about the study rationale, the study procedures and the risks of the study-specific diagnostics. Only after obtaining written informed consent, a screening evaluation to determine the subject's eligibility will be conducted within the days -45 and -14 before CAR T cell administration. Subjects meeting all the inclusion criteria and none of the exclusion criteria will be included into the study.

Following examinations will be performed at the screening visit:

- Written informed consent
- Demography
- Medical history including data of
  - initial diagnosis of systemic autoimmune disease
  - all prior therapies for underlying disease and response to prior therapy
  - relevant prior and all concomitant diseases
  - presence of B-symptoms (defined as fever >38.3°C, drenching night sweats or unexplained weight loss of more than 10% of body mass over past 6 months)
  - other presumably disease associated symptoms like chills, fatigue, pruritus, alcohol induced pain
- Relevant prior and all concomitant medication for other diseases

- Physical examination including height and weight, skin of the whole body, nasal cavities, eyes, ears, and the respiratory, cardiovascular, gastrointestinal, neurological, lymphatic, and musculoskeletal system
- Vital signs (heart rate, blood pressure and temperature)
- Brain MRI
- 12-lead-ECG and echocardiography
- Disease specific assessments:
    - SLE: British Isles Lupus Assessment (BILAG), Systemic Lupus Erythematosus Disease Activity Index (SLEDAI)
    - SSc: Modified Rodnan Skin Score (mRSS)
    - DM/PM: VAS global extramuscular activity, Manual Muscle Testing (MMT)
- Screening laboratory testing for latent tuberculosis (TBC) using QuantiFERON TB-Gold, Hepatitis B (HBsAg and/or HBcAb), Hepatitis C antibodies, HIV1/2 and TSH basal
- Serum antibody levels against tetanus, measles, SARS-CoV-2 and EBV
- Pregnancy testing for female subjects of child-bearing age
- Fertility counseling: Due to the effect of the conditioning regimen on the testes, male subjects should be advised to cryopreserve sperm prior to enrolment, if not already performed before
- Serum-creatinine and eGFR (calculated as CKD-EPI)
- Laboratory assessment (including safety laboratory):
    - Hematology: Full blood count (FBC) with differential white blood cell count (WBC).
    - lymphocyte subsets (CD3, CD4+ and CD8+ T cells, B cells, NK cells incl. CD3 counts [for leukapheresis])
    - Clinical chemistry (extended)
    - Coagulation
    - Immunology:
        - SLE: C3/C4, RF, ACPA, anti-dsDNA (ELISA and Crithidia test), ANA, ENA profile, anti-cardiolipin IgG/M, anti-ß2-glycoprotein IgG/M,
        - SSC: RF, ACPA, ANA, ENA profile, system sclerosis profile,
        - DM: RF, ACPA, ANA, ENA profile, myositis profile.
    - ESR

- Urine analysis

- Adverse Events
- Patient's Global Assessment (PtGA) and Physician's Global Assessment (PhGA) of Disease Activity
- PROs and composite scores:
  - general: HAQ-DI, FACIT Fatigue, QLQ-C30
- Lung function: Forced Vital Capacity (FVC), Total Lung Capacity (TLC) and DLCO (diffusion capacity) in all subjects
- CT Thorax: only in SSc or patients with DM/PM with lung involvement. If HR-CT has been done during the last 6 months as part of clinical routine, no baseline investigation will be necessary.
- Physical function:
  - SLE: accelerometry
  - SSc: Hand function by isometric grip strength, Moberg-Pick-Up Test, sMHQ
  - DM/PM: Physical function by 2-minute-walk with gait assessment
- Complementary Research:
  - Serum levels of BAFF, type I interferon, IFN-gamma, IL-6, TNF-alpha
  - Circulating T lymphocyte subsets
  - T cell and B cell receptor sequencing
  - Single cell RNA sequencing including IFN signature
  - Exploratory biomaterial: stool probe (microbiome), serum and EDTA blood
- Review of inclusion and exclusion criteria: Only subjects who meet the inclusion criteria and none of the exclusion criteria and who are eligible for and who underwent leukapheresis will be considered as enrolled into the study. Subjects who fail to meet the eligibility criteria will be allowed to be re-screened.
- All immunosuppressive treatment such as azathioprine, cyclophosphamide and mycophenolate will be stopped, while belimumab and glucocorticoids (up to 10 mg prednisolone/day) can be maintained until lymphodepletion (day -5).

6.2.2 Leukapheresis (Visit 2)

Leukapheresis will be performed inpatient (Med 3) according to local standard. On the day of leukapheresis prior to the procedure, the following assessments are required:

- Concomitant medication
- Physical examination including weight: the body weight of the subject will be noted on the accompanying leukapheresis sheet. It will be taken for the calculation of the final dose.
- Vital signs
- Safety laboratory
- Adverse Events
- Physical function:
    - SLE: accelerometry
    - SSc: Hand function by isometric grip strength, Moberg-Pick-Up Test, sMHQ
    - DM/PM: Physical function by 2-minute-walk with gait assessment
- SARS-CoV2 antigen testing via mucosal swab

In case of a clinically significant infection prior to leukapheresis, cell collection must be delayed until the infection resolves.

After eligibility confirmation, leukapheresis will be performed on day -13 to obtain leukocytes for the manufacturing of MB-CART19.1.

Cells obtained from apheresis will be transferred to the manufacturing site on the same day.

6.2.3 Lymphodepletion (Visit 3)

Lymphodepletion will be performed inpatient (Med 5). Prior to the start of lymphodepleting conditioning therapy (cyclophosphamide and fludarabine, days - 6 to -3), the following assessments are required:

6.2.3.1 Prior to lymphodepletion procedure

- Check of additional criteria before lymphodepletion (see section 7.3.3)
- Concomitant medication

- Physical examination
- Vital signs
- Chest X-ray to check the position of the central venous catheter
- Pregnancy testing for female subjects of child-bearing potential
- Laboratory: Hematology, clinical chemistry (extended), coagulation, urine analysis, Ig serum levels
- Adverse Events
- PtGA and PhGA of Disease Activity
- PROs and composite scores
- Persistence and phenotype of MB-CART19.1 in peripheral blood
- Complementary Research:
    - Serum levels of BAFF, type I interferon, IFN-gamma, IL-6, TNF-alpha
    - Circulating T lymphocyte subsets

For subjects with eGFR between 30 and 70 ml/min/1.73m^2 the recommended Fludarabine dose should be reduced by up to 50% and the eGFR of the subject should be monitored closely according to the institutional standards (at least once daily).

Additionally, before lymphodepletion the confirmation of the manufacturer must be available that the in-process-control of the drug substance is “passed”.

6.2.3.2 Days -4 and -3

- Concomitant medication
- Physical examination
- Vital signs
- Adverse Events
- Safety laboratory on day -3 only

For subjects eGFR between 30 and 70 ml/min/1.73m^2 the recommended Fludarabine dose should be reduced by up to 50% and the eGFR of the subject should be monitored closely according to the institutional standards (at least once daily).

6.2.4 Treatment phase (Visit 4)

Subjects are already inpatients due to the previous lymphodepletion and will remain hospitalized for administration of the ATMP and a subsequent observation period for a minimum of 10 days (see section 8.2.3). Safety and concomitant medication use will be assessed throughout treatment. SAEs will be graded according to CTCAE (version 5.0) and should be followed until no further change can be expected.

6.2.4.1 Day 0, pre-dose

- Check of additional criteria before IMP administration (see section 7.3.4)
- Concomitant medication
- Physical examination
- Vital signs
- Continuous ECG (until end of infusion)
- Laboratory: hematology, lymphocyte subsets, clinical chemistry (extended), coagulation (extended), immunology, ESR, urine analysis, additional safety parameters (serum IL-6, procalcitonin and ferritin), CMV-DNA and beta-d-glukan
- Adverse Events
- Neurological examination
- PtGA and PhGA of Disease Activity
- PROs and composite scores
- Complementary Research:
    - Serum levels of BAFF, type I interferon, IFN-gamma, IL-6, TNF-alpha
    - Circulating T lymphocyte subsets
    - Persistence and phenotype of MB-CART19.1 in peripheral blood

6.2.4.2 Day 0, dosing

The IMP will be administered as an intravenous infusion.

Recordings of continuous ECG will be stopped after end of infusion.

6.2.4.3 Day 0, post-dose (1 and 2 hours)

- Concomitant medication
- Vital signs
- Adverse Events

6.2.4.4 Day 0, post-dose (4 hours)

- Concomitant medication
- Vital signs
- Adverse Events

6.2.5 Short-term Follow-up (Visit 5-10)

<u>On days 1, 2, 7, 10, 14 and 28 the following assessments will be performed:</u>

- Concomitant medication
- Physical examination
- Vital signs
- Laboratory: hematology (all visits), clinical chemistry basic (V5, V6, V8, V9), clinical chemistry extended (V7, V10), coagulation basic (V5, V8, V9, V10), coagulation extended (V6, V7), immunology (V7, V9, V10), ESR (V7, V9, V10), urine analysis (V7, V9, V10), additional safety parameters (all visits), CMV and beta-d-glukan (V7), Ig serum levels (V10)

It is recommended to take samples daily if a subject suffers of fever, CRS and/or neurotoxicity until resolution. Lab results, which are related to any AE, should be entered in the CRF.

- Adverse Events
- Neurological examination
- Complementary research (V6-V10)
  - Persistence and phenotype of MB-CART19.1 in peripheral blood (on days 2, 7, 10, 14 and 28 only)

Additional assessments for day 7 and day 28 (V7, V10):

- PtGA and PhGA of Disease Activity
- Disease specific PROs and composite Scores
- Questionnaires (HAQ, FACIT, QLQ-C30)
- Physical function (day 28 only):
  - SLE: accelerometry
  - SSc: Hand function by isometric grip strength, Moberg-Pick-Up Test, sMHQ
  - DM/PM: Physical function by 2-minute-walk with gait assessment

6.2.6 Mid-term Follow-up (Visits 11-14)

In the second follow-up phase until week 24, the following assessments will be performed (outpatient):

6.2.6.1 Week 8 (Visit 11)

- Concomitant medication
- Physical examination
- Vital signs
- Laboratory: hematology, clinical chemistry (basic), coagulation (basic), immunology, ESR, urine analysis, Ig serum levels
- Adverse Events

- PtGA and PhGA of Disease Activity
- PROs and composite scores
- Complementary Research:
    - Serum levels of BAFF, type I interferon, IFN-gamma, IL-6, TNF-alpha
    - Circulating T lymphocyte subsets
    - Persistence and phenotype of MB-CART19.1 in peripheral blood

6.2.6.2 Week 12 (Visit 12)

- Concomitant medication
- Physical examination
- Vital signs
- Laboratory: hematology, clinical chemistry (extended), coagulation (basic), immunology, ESR, urine analysis, Ig serum levels
- Adverse Events
- Neurological examination
- PtGA and PhGA of Disease Activity
- PROs and composite scores
- Physical function:
    - SLE: accelerometry
    - SSc: Hand function by isometric grip strength, Moberg-Pick-Up Test, sMHQ
    - DM/PM: Physical function by 2-minute-walk with gait assessment
- Complementary Research:
    - Serum levels of BAFF, type I interferon, IFN-gamma, IL-6, TNF-alpha
    - Circulating T lymphocyte subsets
    - Persistence and phenotype of MB-CART19.1 in peripheral blood

After the end of week 12, i.e., with start of week 13, all SAEs and AESI and their related concomitant medication will be documented and reported until week 24.

6.2.6.3 Week 16 (Visit 13)

- Concomitant medication related to SAEs/AESI
- Physical examination
- Vital signs
- Laboratory: Hematology, clinical chemistry (basic), coagulation (basic), immunology, ESR, urine analysis, Ig serum levels
- SAEs/ AESI

6.2.6.4 Week 24 (Visit 14)

- Concomitant medication related to SAEs/AESI
- Physical examination
- Vital signs
- Laboratory: hematology, clinical chemistry (extended), coagulation (basic), immunology, ESR, urine analysis, Ig serum levels, serum antibody levels
- SAEs/ AESIs
- PtGA and PhGA of Disease Activity
- PROs and composite scores
- Lung function: Forced Vital Capacity (FVC), Total Lung Capacity (TLC) and DLCO (diffusion capacity) only in SSc and DM/PM with pulmonary involvement
- CT Thorax: only in SSc. If HR-CT has been done during the last 6 months as part of clinical routine, no baseline investigation will be necessary.
- Physical function:
  - SLE: accelerometry

    - SSc: Hand function by isometric grip strength, Moberg-Pick-Up Test, sMHQ
    - DM/PM: Physical function by 2-minute-walk with gait assessment
- Complementary Research:
    - Serum levels of BAFF, type I interferon, IFN-gamma, IL-6, TNF-alpha
    - Circulating T lymphocyte subsets
    - T cell and B cell receptor sequencing
    - Single cell RNA sequencing
    - Persistence and phenotype of MB-CART19.1 in peripheral blood
    - Stool Microbiome and secretory IgA

6.2.7 Additional Response-Assessment Visit

In case a subject experiences a relapse or a disease progression or early discontinues the active part of the study after IMP dosing the Investigator will make any reasonable effort to collect and report the following assessments. Please note, that a disease status assessment should be performed before any other therapeutic intervention (see sections 7.6.2 and 7.6.3).

- Concomitant medication, and in case after week 12: related to SAEs/AESI only
- Physical examination,
- Serum IgG, IgA and IgM levels and IgG subclasses
- Safety laboratory: hematology, clinical chemistry, coagulation, immunology, ESR
- Urine analysis
- SAEs, AESI and SARs,
- PtGA and PhGA of Disease Activity
- PROs and composite scores,
- Persistence and phenotype of MB-CART19.1 in peripheral blood

6.2.8 Safety Follow-up Visits (e.g. for flare of disease)

In case a subject cannot follow the investigations as planned in the active part of the study after IMP dosing, the Investigator will make any reasonable effort to collect and report the following

safety assessments at the appropriate time points when the regular visits should take place according to the visit schedule:

- Serum IgG, IgA and IgM levels and IgG subclasses
- Safety laboratory I: hematology, clinical chemistry, coagulation, immunology, ESR
- SAEs, AESI and SARs
- Persistence and phenotype of MB-CART19.1 in peripheral blood (only at same time points foreseen according to regular visits (V6-V12, V14))
- Complementary research (only at same time points foreseen according to regular visits (V9-V12, V14)).

In case the subject does not return to the site for any reason the Investigator should try to reach the subject via phone for AE documentation and reporting as defined in section 10.1.2.

6.2.9 Unscheduled Visits

It is at the discretion of the Investigator to appoint additional visits as medically necessary. The date and reason should be documented. Measures and assessments will be performed according to Investigator's decision and be documented in the CRF. An unscheduled visit will not affect the scheduling of the planned visits, i.e. the following regular visit will not be postponed.

6.2.10 Continuation of Treatment after the End of the Clinical Study

After completion of the study subjects will be further monitored for disease symptoms.

- If in remission, no treatment will be given.
- If disease recurs, glucocorticoids and immunosuppressive treatment (dependent on previous exposure, tolerance and efficacy) will be started.
- If study subjects do not improve in disease activity, glucocorticoids and immunosuppressive treatment (dependent on disease, previous exposure, tolerance and efficacy) will be started.

6.2.11 Complementary Research Program

Blood for immune monitoring assays for the complementary research program will be collected as described in the Assessment Schedule and in sections 9.4.

7 STUDY POPULATION

7.1 Selection of study population

Subjects will be selected among adult subjects with a confirmed diagnosis of an active B cell driven autoimmune disease SLE, SSc and DM/PM, according to ACR Classification criteria (ICD 10 GM M32, M33, M34).

7.2 Gender distribution

Both male and female subjects with SLE, SSc and DM/PM will be enrolled in this clinical study. As the IMP is not expected to differ in its effects in male and female subjects, subject recruitment does not take account of the distribution of gender.

7.3 Inclusion criteria

The study will enroll adult subjects with the following three systemic autoimmune diseases: (a) Systemic Lupus Erythematosus (SLE), (b) Systemic Sclerosis (SSc) and (c) Dermatomyositis/Polymyositis (DM/PM).

Only subjects meeting all of the following inclusion criteria at screening will be considered for study enrollment:

7.3.1 General inclusion criteria

- Subjects must understand and voluntarily sign an informed consent form including written consent for data protection,
- Adults aged ≥ 18 years at time of consent,
- Adequate renal (eGFR > 30 ml/min/m^2), liver (no Child Pugh C), heart (at worst NYHA III, EF > 30%) and pulmonary (FV and DLCO ≥ 30%) function,
- Male subjects unless surgically sterile, must agree to use two acceptable methods for contraception (e.g. spermicide and condom) during the trial and refrain from fathering a child starting from the time of signing the Informed Consent Form (ICF) until 12 months after dosing of the IMP,

- Females of childbearing potential (FCBP) must have a negative urine pregnancy test at screening and must agree to use a highly effective contraceptive method (Pearl index <1) starting from the time of signing the ICF and for 12 months after dosing of the IMP,
- Must be able to adhere to the study visit schedule and other protocol requirements,
- Double vaccination against SARS-CoV-2 or SARS-CoV-2-Infection within the last 6 months.

7.3.2 Disease-specific inclusion criteria

7.3.2.1 SLE subjects

- Fulfilling the 2019 ACR/EULAR classification criteria of SLE ([64]),
- Positivity of anti-dsDNA (> 4 U/l), anti-histone (+ or more), anti-nucleosome (+ or more) or anti-Sm antibodies (+ or more) at screening or by documented medical history,
- Active disease at screening, defined as ≥ 1 organ system with a British Isles Lupus Assessment (BILAG) A score (severe disease activity) or ≥ 2 organ systems with a BILAG B score (moderate disease activity) ([58]),
- *Insufficient response or intolerance/ contraindication to glucocorticoids and to at least 2 of the following treatments:* azathioprine, hydroxychloroquine, mycophenolate mofetil, belimumab, methotrexate, *rituximab, cyclophosphamide. Insufficient response is defined as having increased disease activity based on the definition explained in the previous bullet point.*

7.3.2.2 SSc subjects

- Fulfilling the 2013 ACR/EULAR classification criteria of SSc ([14]),
- Positivity (+ or more) for at least one SSc-specific parameter (Scl70, RNA polymerase, Th/To, RP11/12, U3RNP autoantibodies) at screening or by documented medical history,
- Signs for fast progression including (i) disease duration ≤ 7 years (from onset of first non-Raynaud manifestation), (ii) mRSS score 10-35 at screening, (iii) elevated acute phase reactant levels (CRP ≥ 6 mg/L, ESR ≥ 28mm/h or platelet count ≥ 330 G/L), (iii) mRSS

increase ≥ 3 units or involvement of one new body area or mRSS increase ≥ 2 units in one body area or ≥ 1 tendon friction rub over 6 months ([56]),

- *Insufficient response or intolerance/ contraindication to at least 2 of the following treatments: m*ycophenolate mofetil, *azathioprine, cyclophosphamide,* nintedanib, methotrexate*, rituximab. Insufficient response is defined as having increased disease activity based on the definition explained in the previous bullet point.*

7.3.2.3 DM/PM subjects

- Fulfilling the 2017 ACR/EULAR classification criteria for probable or definite DM or PM ([65]),
- Presence of active myositis in muscle biopsy or muscle MRI and/or signs of interstitial lung disease related to DM/PM,
- Positivity (+ or more) for at least one myositis-specific antibody (aminoacyl tRNA synthetases, Mi2, MDA5, SAE, SRP, ARS, HMGCR, MJ, TIF1gamma) at screening or by documented medical history,
- In patients with active myositis: Muscle weakness as define by MMT < 142 and 2 of the following criteria: VAS patients Global ≥2cm, VAS physician Global ≥ 2cm, HAQ > 0.25, at least one muscle enzyme > 1.3 times upper limit of normal, VAS global extra muscular activity ≥ 2cm,
- *Insufficient response or intolerance/ contraindication to glucocorticoids and to at least 2 of the following treatments:* azathioprine, *cyclophosphamide, m*ycophenolate mofetil, ciclosporin A, tacrolimus, methotrexate, rituximab, *intravenous immunoglobulins. Insufficient response is defined as having increased disease activity based on the definition explained in the previous bullet point.*

7.3.3 Additional criteria before lymphodepletion

- no evidence of a clinically significant active infection,
- serum creatinine < 2xULN,
- normal vital parameters, and

- no acute central neurological toxicity.

7.3.4 Additional criteria before IMP administration

- no evidence of a clinically significant active infection, and
- no acute central neurological toxicity.

7.4 Exclusion criteria

Subjects having any of the following criteria at screening will not be included in the study:

- Clinically suitability for a less burdensome and/or approved therapeutic approach, as judged by the investigator,
- ANC < 1.000/mm3, ALC < 500/mm^3 or hemoglobin < 8g/dl
- Uncontrolled severe concomitant disease, such as cancer (except basal or squamous cell skin cancer) and diabetes mellitus,
- Severely impaired renal (eGFR ≤ 30 ml/min/m^2), liver (Child Pugh C), heart (NYHA IV, EF ≤ 30%) and pulmonary (FV and DLCO < 30%) function,
- Any condition, including the presence of laboratory abnormalities, which places the subject at unacceptable risk if he/she were to participate in the study or confounds the ability to interpret data from the study,
- Prior treatment with anti-CD19 antibody therapy, adoptive T cell therapy or any prior gene therapy product (e.g. CAR T cell therapy),
- History of bone marrow/ hematopoietic stem cell or solid organ transplantation,
- Any concomitant severe active infection, e.g. HIV, hepatitis B or C, SARS-CoV 2 (COVID 19), or active tuberculosis as defined by a positive Quantiferon TB-test. If presence of latent tuberculosis is established then treatment according to local guidelines must have been initiated prior to enrollment,
- Diagnosis of severe neuropsychiatric SLE, inclusion body myositis or limited SSc,
- Pregnant or lactating females,

- Females who are intending to conceive during the study,
- Known hypersensitivity to any drug components,
- Malignancy in the last 5 years before screening,
- Requirement for immunization with live vaccine during the study period or within 14 days preceding leukapheresis,
- Subjects who are younger than 18 years or are incapable to understand the aim, importance and consequences of the study and to give legal informed consent,
- Have a history of alcohol or substance abuse within the preceding 6 months that, in the opinion of the Investigator, may increase the risks associated with study participation or study agent administration, or may interfere with interpretation of results,
- Subjects who possibly are dependent on the Sponsor, the Principal Investigator or Investigator (e.g. family members).

7.5 Assignment to treatment

No randomization is planned for this open label study.

Only subjects who comply with all in- and exclusion criteria and who are eligible for leukapheresis will be assigned to the course of the study treatments. Eligibility criteria will be verified and ineligible subjects will proceed off-study and no further follow-up will be obtained.

Subjects willing to participate in the study will sign an approved consent form prior to any study relevant treatment.

At the screening visit (V1) subjects will be assigned to an ascending 6-digit subject identification number, consisting of a one-digit site number, followed by a two-digit subject number, and a three-digit disease acronym (SLE, SSC, DPM), i.e. x-yy-zzz. Subject numbers will be assigned in a consecutive manner in the order of inclusion, e.g. the first subject who signs ICF receives the first number (for example at site 1 with diagnosed SLE: 1-01-SLE), the second subject receives the next number in sequence and so on.

This 6-digit subject identification number will be recorded on all study-specific documents of a specific subject, in the site subject file as well as in the Case Report Forms (CRFs).

The Investigator will keep a record with the names, the birth dates and the subject identification number of the subjects (subject identification list) to allow checking of data in the clinical files of each subject, when required. This record will remain exclusively at the study site.

7.6 Subject completion and discontinuation

7.6.1 Definitions

A **'completed'** subject is one, who has completed the trial until week 24 and data for primary endpoint evaluation is available. Afterwards, they will roll-over to a subsequent follow-up study collecting safety data at annual visits for up to 15 years after IMP infusion.

A **'screening failure'** is a subject who, after having signed the informed consent, failed a screening examination or is not eligible for IMP administration. Screening failures will be replaced.

A **'drop-out'** is a subject who stops prematurely after IMP administration due to lost to follow-up or withdrawal of consent. Drop-outs will not be replaced.

7.6.2 Handling of flare of disease

Flare of disease is not a considered reason for discontinuation of the study. Those subjects will stay in the study until week 24, withdrawal or death.

Flare of disease is defined as an AESI. It will be followed-up until resolution, loss to follow-up or death.

Subjects should receive a response assessment before any other therapeutic intervention (see section 6.2.9). In case of a disease flare, subjects may receive immunosuppressive medication at the discretion of the investigator such as glucocorticoids. Start and type of sequential therapy has to be documented.

Subjects will continue the visit schedule as planned, but with reduced evaluations - as “Safety Follow-up Visits” until week 24.

7.6.3 Procedure for handling discontinuations

All subjects are free to discontinue participation in this study at any time, for any reason, specified or unspecified, and without penalty or loss of benefits to which the subject is otherwise entitled. With respect to possible side effects of the IMP and the corresponding regulatory requirements for complete follow-up of subjects until 24 weeks after treatment, Investigators will make every

reasonable effort to keep each subject on the study until all planned assessments have been performed. If the reason for discontinuation allows the Investigator should identify if the subject wants to withdraw completely from the study and any follow-up, or if the subject accepts safety follow-up and the consent for this remains. The subject's decision and the follow-up activities should be appropriately documented.

The reasons for early discontinuation from participation of the study may include the following:

- The subject withdraws consent,
- The subject drops out from the study or fails to return to the site and does not respond to the attempts of the investigative site to contact him (lost to follow-up),
- Subject's death.

The Sponsor must be informed of any subject discontinuation (regardless of cause) within 24 hours.

Regardless of the reason for discontinuation, all subject data required at the time of discontinuation should be recorded in the CRF; the date and all reasons for discontinuation must be documented in subject's records.

When a subject, treated with the study drug, discontinues the study prematurely, Investigators will make any reasonable effort to collect and document "Additional Response-Assessment Visit" evaluations (section 6.2.7). The response assessment should be performed before any other therapeutic intervention. Frequency of further "Safety Follow-Up Visits" will be according to the regular visit schedule. The "Safety Follow-Up Visits" will consist of the evaluations described under section 6.2.8. They have to be conducted until week 24 after study drug treatment.

In case a subject drops-out, e.g. he/she fails to return to the site and does not respond to two documented attempts of the investigative site to contact him by a combination of telephone/email and mail within one month (reason: lost to follow-up) no additional contact will be required.

8 STUDY MEDICATION AND ADMINISTRATION

8.1 Lymphodepletion

All subjects will receive fludarabine 25 mg/m^2/d intravenously (i.v.) on days -5, -4, and -3, and cyclophosphamide 1000 mg/m2/d iv on day -3 for lymphodepletion. A minimum interval of 48 hours after infusion of cyclophosphamide prior to administration of IMP has to be held.

For subjects with eGFR between 30 and 70 ml/min the fludarabine dose will be reduced by up to 50% and the eGFR of the subject will be monitored closely according to the institutional standards (at least once daily).

Subjects will be hospitalized for lymphodepleting chemotherapy as well as for the administration of MB-CART19.1 and for at least 10 days.

Supportive concomitant medication will be administered according to local standards, e.g. hydration and mesna should be added according to institutional guidelines.

The lymphodepleting treatment will only be started after confirmation of a successful lentiviral transduction by the manufacturer, i.e., the result of the In-Process-Control (IPC) on day 5 of the manufacturing process will be forwarded to the site.

8.2 Investigational Medicinal Product MB-CART19.1

Fresh ATMP from autologous T cells ex vivo lentivirally transduced with the vector (anti-CD19 CAR pLTG1563) and expanded. Leukapheresis will be performed at the collection site according to local standard practice. The leukapheresis product of the subject will be used for the individual manufacturing of MB-CART19.1 by using the automated CliniMACS Prodigy System. CD4+ and CD8+ cells are enriched and activated, followed by lentivirus-based transduction with the CD19 CAR construct. Then the CD19 CAR transduced T cells are expanded and finally formulated (ATMP). ATMP manufacturing will be performed in certified location in Erlangen under licenses by local regulatory authorities. T cells are activated, lentivirally transduced, expanded, and formulated. Quality controls are performed in-process and on the final product.

8.2.1 Manufacturing of the IMP

8.2.1.1 Leukapheresis (Day -13)

The IMP will be manufactured from a fresh donor leukapheresis product (Leukapheresis, day -13). Leukapheresis will be performed at the collection site according to local standard practice.

8.2.1.2 Preparation (Day -12 to -1)

The leukapheresis product will be used for automated CD19.1 CAR cell production. In brief, CD4+ and CD8+ positive T cell will be enriched, activated and lentivirally transduced. After in vitro expansion in presence of IL-7 and IL-15 the cells will be harvested, and formulated in CliniMACS Formulation Solution(R). Quality control includes number of cells, transduction rate, composition of cell populations, microbiological quality, endotoxin content, absence of *Mycoplasma sp.* in the cellular product and in-process controls as well as vector copy number determination. Manufacturing and quality control will occur according to established processes within the existing manufacturing licence (UK Erlangen:DE_BY_05_MIA_2022_0081/55.2-2678.3-33-7-28). The IMP will be applied freshly (without cryopreservation).

8.2.1.3 Packaging

The IMP "MB-CART19.1" will be delivered in primary bags, which are packed for transport in a secondary overwrap package.

8.2.1.4 Labeling

The bags/packages of the IMPs will be labeled in accordance with the GMP Annex 13, German Drug Law (AMG) and the GCP regulation ("GCP-Verordnung") §5. Labels will be in local language.

8.2.1.5 Storage

The IMP "MB-CART19.1" is intended for direct administration after completion of the preparation process with a shelf-life of 24 h calculated from the end of the manufacturing process, to application with a storage temperature of 2-8°C.

Temperature during storage of IMP will be controlled and documented.

8.2.2 Transport of the IMP

The transport of the IMP from the GMP Manufacturing site to the clinical study site will be performed at product-appropriate temperatures at 2-8°C. The transport will be started after end of the manufacturing process to arrive either the same day or the day after.

8.2.3 Dosage of the IMP (Day 0)

All subjects will be hospitalized for administration of the ATMP and a subsequent observation period for a minimum of 10 days.

Subjects are not allowed to be discharged from the hospital until CAR T cell-related CRS symptoms (fever, hypotension, hypoxia) have fully resolved, nor with ongoing > grade 1 CNS toxicities.

8.2.3.1 Dosing schedule: single dose i.v.

In the first stage of the study, 8 subjects will be recruited and treated with $1x10^6$ CAR T cells/kg body weight. An interval of at least 28 days between the treatment of the first and second subject is mandatory. If a DLT occurs in 2 subsequent subjects, the dose of ATMP will be reduced from 1 to $0.5 x10^6$ CAR T cells/kg body weight. If additional 2 DLTs occur in these doses, the study will be stopped.

If no more than 3 DLT occur and if clinical efficacy is observed in 4 or more subjects, the second stage (basket) will be started. In the second stage of the study, 16 additional subjects will be recruited.

8.2.3.2 Measures in case of “failed” In-Process-Control (IPC) during Manufacturing

In case the manufacture of the IMP has to be discontinued due to the negative results of the IPC the Sponsor informs the Investigator. The subject will not start the lymphodepleting chemotherapy and will be considered a withdrawal. The subject can be re-screened if possible and would receive a new subject identification number in that case. Results of the first screening examination are likely to be reused (as far as reasonable).

8.2.3.3 Measures in case subject is unable to be dosed on Day 0

In case the subject suffers from an AE which prohibits the infusion of MB-CART19.1 the infusion can be postponed for the remaining time until expiry of the IMP (see 6.2.4.1). Thereafter the

subject has to be considered a withdrawal. The subject can be re-screened if possible. Results of the first screening examination are likely to be reused (as far as reasonable).

If the subject dies prior to completion of the IMP production the Sponsor notifies the manufacturer to stop the IMP production immediately after the Sponsor has been informed by the Investigator. The IMP has to be discarded.

8.2.3.4 Measures in case of insufficient cell numbers of IMP

In case, the produced cell number is not sufficient, i.e., the cell number does not reach the anticipated dose, the manufacturing site will inform the Sponsor immediately. Depending on the anticipated cell dose, the Sponsor will request to repeat the leukapheresis procedure.

8.2.3.5 Measures in case of IMP being Out Of Specification (OOS)

An IMP, which is out of specification for any reasons, may be provided by the Qualified Person upon request by the Investigator and thus may be administered to the subject. Risk assessments, communication with local and competent authorities and decisions need to be clearly documented in the batch records. The Investigator will be informed by the CTM in due time.

Subjects will be followed up per clinical study protocol if they receive the treatment that is out of specifications. In case they do not receive the treatment, they will be entered in the database and safety follow-up until day 28 will be performed. In addition, further treatment and its outcome will be documented.

When IMP is administered prior to completion of day 14 microbial (sterility) testing, it is possible that a sterility test returns positive after IMP administration. In that case, the Investigator will be notified to follow site-specific procedures for positive cultures after IMP infusion. Procedures should include appropriate infectious work-up and antimicrobial coverage as indicated based on the results of the sterility tests.

The Investigator and the Sponsor will be responsible for regulatory reporting. Subjects will be treated as receiving out-of-specification investigational agents, and will be replaced but followed up per protocol for safety.

8.2.4 Preparations and administration of IMP

MB-CART19.1 is delivered freshly after manufacturing and administered intravenously (i.v.) as single dose the final volume of which is adapted to the subjects' weight. Infusion will be performed through a central or peripheral vein over a time period of approximately 5 to 20 minutes.

Administration of the IMP has to be completed within the shelf-life indicated on the label.

For CAR T cell infusion, the following preparations need to be performed:

- Prepare monitoring of vital parameters.
- Administer pre-medication: H1-antagonists like clemastine 0.025 mg/kg i.v. (max 2 mg), and paracetamol 10 mg/kg p.o. or i.v. (max 1000 mg) prior to infusion (recommended).
- Infusion set.
- If possible, a central venous catheter should be in place.

The pre-medication should be completed 15 minutes before CAR T cell infusion. A continuous ECG will be applied and has to be recorded until the end of infusion of MB-CART19.1. Standby medication with steroids should be avoided whenever possible. During infusion, the subject should be monitored by pulsoxymetry and intermittent blood pressure control. After CAR T cell infusion, the subject should be monitored according to section 6.2.4.

For medical interventions in case of toxicities please refer to section 6.1.6.

8.3 Method of blinding

This is an open-label study.

8.4 Drug accountability

Accountability for the IMP is the responsibility of the Investigator, who may delegate this responsibility to appropriately qualified personnel. The responsible person will ensure that MB-CART19.1 are used only in accordance with this protocol and that appropriate records are maintained. Autologous MB-CART19.1 are supplied for study subjects only and must not be used outside the context of this protocol.

The Investigator (or other appropriately qualified personnel) must maintain records for the IMP, which includes receipt, dispensing, unused medication, storage conditions and destruction of unused medication.

Accountability for drugs other than the IMPs (e.g. lymphodepleting conditioning therapy and/or other concomitant medication) is to be performed according to institutional guidelines.

Copies of completed drug accountability logs must be submitted to the Sponsor for all study subjects at the end of treatment or upon request.

8.5 Auxiliary Medicinal Products / Non-Investigational Medicinal Products

Auxiliary medicinal products in this study include

- agents used for lymphodepletion, specifically fludarabine and cyclophosphamide,
- administration of uromitexan (MESNA) during lymphodepleting chemotherapy (see 8.6.2),
- antibiotic prophylaxis such as cotrimoxazole as well as antiviral prophylaxis such as aciclovir dependent on leucocyte count during lymphodepletion (see 8.6.2),
- agents used for pre-medication, specifically antihistaminic drugs like clemastine and paracetamol (see 8.2.4),
- intravenous immunoglobulin prophylaxis used to substitute low IgG levels (<400 mg/dL), which are a consequence of CAR T cell induced B cell depletion (see 6.1.6),
- Tocilizumab as rescue therapy for CRS, and corticosteroids as second line rescue therapy (see 6.1.6),
- Antileptic medication, e.g. levetiracetam, as standard rescue therapy for seizures by neurotoxicity (see 6.1.6).

Auxiliary medicinal products are used and accounted for according to internal hospital routine.

8.6 Concomitant medication

Subjects may take any medication that is not restricted by the protocol (section 8.6.4) and that would not be expected to interfere with the conduct of the study. Chronic medication should be dosed on a stable regimen throughout study participation (except as medically required due to an AE). All concomitant medication is recorded in the CRF "Concomitant Medication": generic name,

indication, route of administration, dose rate with unit of measurement, date started (before study or date), date stopped (ongoing or date) and application (continuous or as necessary). In case of changes in the dosage or regimen of a concomitant medication, the maximal dose will be recorded in the CRF.

Concomitant medication will not be recorded after end of week 12 except they are related to SAE treatment (SAR/AESI).

8.6.1 Concomitant medication at Screening

At Screening, subjects will be asked what medication they are currently taking. At each subsequent assessment, any new concomitant medication and any changes in concomitant medication will be documented.

- All immunosuppressive treatment such as azathioprine, cyclophosphamide and mycophenolate will be stopped at the screening visit, while belimumab and glucocorticoids (≤ 10 mg prednisolone/day) can be maintained until lymphodepletion (day -5).
- Concomitant use of oral corticosteroids (≤ 5 mg/day oral prednisone or equivalent) is allowed during the study and should be kept at a stable dose for ≥ 1 week prior to leukapheresis and remain stable throughout the study. Inhaled corticosteroids for stable medical conditions are allowed.
- The use of NSAIDs, including COX2 inhibitors and paracetamol, are allowed during the study, if subjects receiving stable prescribed doses for ≥ 1 week before leukapheresis. In the event of tolerability or other safety issues, the doses of NSAIDs may be skipped, decreased, or discontinued with substitution by another permitted medication from that class. If taking any of the above at leukapheresis on an as-needed basis, they should continue to use them for the same reason and same dose each time. On the days the subjects are scheduled to be seen in clinic, no NSAIDs should be used within 24 hours of the subject´s clinic visit.

8.6.2 Concomitant medication during lymphodepletion

During lymphodepleting chemotherapy with cyclophosphamide (days -5 to -3) adequate hydration as well as concomitant administration uromitexan (MESNA) prophylaxis in accordance with local standards is mandatory.

Pneumocystis pneumonia (PCP) prophylaxis with cotrimoxazole and an anti-viral prophylaxis with aciclovir until a CD4 T-Cell count >200/µl is reached will be performed in any patient while ciprofloxacin will be given to neutropenic patients and antiemetic prophylaxis is performed according to hospital standards.

8.6.3 Prohibited concomitant medication

Immunosuppressants, glucocorticoids (except flare control as described in 8.6.4) and live-vaccines until week 24.

8.6.4 Concomitant medication in case of flare

In case of a flare until week 24, two prednisolone pulses of 20 mg prednisolone equivalent with a complete tapering are allowed. Cumulative prednisolone intake will be noted in CRF. For further handling of flare please refer to sections 6.2.9 and 7.6.2.

8.7 Rescue medication

Relapse of autoimmune disease can occur at any time after CAR T cell therapy. Standard-of-care treatment will be started in case of relapse of autoimmune disease, which depends on the clinical presentation of the relapse. In case of recurrence of SLE, DM/PM and SSc glucocorticoids at a dose of 1 mg/kg body weight prednisolone will be started with subsequent tapering of dose over 6 weeks to a target dose of 5 mg prednisolone/day. In case of recurrence (in SLE and DM/PM) or progression (SSc) of inner organ involvement, additional immunosuppressive therapy will be started. Selection of immunosuppressive therapy will be based on previous medications, tolerability and existing clinical evidence with a preference on mycophenolate mofetil (MMF) at a dose of 3 g/day. MMF is well tolerated, currently used in SLE, DM/PM and SSc treatment and based on our experience many patients are pre-exposed to this drug. For SLE patients, in which hydroxychloroquine is an anchor drug, hydroxychloroquine will be additionally started in case of any (non-organ or organ involvement) disease relapse.

9 METHODS OF ASSESSMENTS

The assessment schedule from screening until 24 weeks post MB-CART19.1 administration is outlined in the Assessment Schedule.

Date of visits and any data generated during visits and assessments will be documented in the source documents. Source documents for unscheduled visits will also be maintained. An unscheduled visit will not affect the scheduling of the planned visits, i.e. the following regular visit will not be postponed due to an unscheduled visit. Evaluation of data with importance to create study endpoints and safety will be recorded within the CRF. The date of the visit and any data generated will be recorded on the appropriate part of the CRF.

9.1 Medical history and concomitant medication

At the screening visit, the subject's medical history as well as relevant prior and all concomitant medications will be recorded. A review of this information will allow the Investigator to assess whether the subject should be enrolled.

9.2 Disease activity assessments

If not stated otherwise, the assessments and questionnaires listed below will be performed at visits V1, V3, V4, V9-V12 and V14.

9.2.1 General assessments and questionnaires

9.2.1.1 Patient's and Physician's Global Assessment (PtGA/ PhGA) of disease activity

The subject and the physician will be independently asked to place a vertical line on a 100-mm VAS on which the left-hand boundary represents "no disease activity," and the right-hand boundary represents "maximum disease activity." The distance from the mark to the left-hand boundary will be recorded.

9.2.1.2 Health Assessment Questionnaire-Disability Index (HAQ-DI)

The HAQ-DI assesses a subject's level of functional ability and includes questions of fine movements of the upper extremity, locomotor activities of the lower extremity, and activities that involve both upper and lower extremities. There are 20 questions in eight categories of functioning, which represent a comprehensive set of functional activities – dressing, rising, eating,

walking, hygiene, reach, grip, and usual activities. The stem of each item asks for the past week "Are you able to …" perform a particular task. The subject's responses are made on a scale from 0 (no disability) to 3 (completely disabled). Each category contains at least two specific component questions. The questionnaire will be provided in a German translation and will be scored based on the instructions from the Stanford University Medical Center.

9.2.1.3 Functional Assessment of Chronic Illness Therapy – Fatigue (FACIT Fatigue)

The FACIT-fatigue scale is a 13-item subject-reported measure of fatigue with a 7-day recall period. Items are scored on a 0-4 response scale with anchors ranging from "Not at all" to "Very much so". To score the FACIT-fatigue, all items are summed to create a single fatigue score with a range from 0-52.

9.2.1.4 EORTC QLQ-C30

The EORTC QLG Core Questionnaire (EORTC QLQ-C30) is a 30-item instrument designed to measure quality of life in all cancer patients.

9.2.2 SLE specific assessments and questionnaires

9.2.2.1 British Isles Lupus Assessment Group (Easy-BILAG) index

The BILAG index is a computerized index for measuring clinical disease activity of lupus over the past 4 weeks. It includes up to 18 questions about eight organ systems that are answered as 0 = not present, 1 = improving, 2 = same, 3 = worse, and 4 = new. Then, a computer program facilitates scoring from numerical to alphabetical score for each system (grade A–E) where grade A represents very active disease and grade E indicates no current disease activity ([58]).

9.2.2.2 Systemic Lupus Erythematosus Disease Activity Index (SLEDAI)

The SLEDAI is a clinical index for the assessment of lupus disease activity in the preceding 10 days. It consists of 24 weighted clinical and laboratory variables of 9 organ systems. The scores of the descriptors range from 1-8, and the total possible score for all 24 descriptors is 105 ([59]).

9.2.3 SSC specific assessments and questionnaires

9.2.3.1 modified Rodnan Skin Score (mRSS)

The mRSS is a measure of skin thickness by estimating skin thickness using a 0-3 scale in 17 body areas. It is recommended that the same assessor examine the subject for the duration of the study ([60]).

9.2.4 DM/PM specific assessments and questionnaires

9.2.4.1 Physician's Global Assessment (PhGA) of extramuscular activity

The physician will be asked to place a vertical line on a 100-mm VAS on which the left-hand boundary represents "no disease activity," and the right-hand boundary represents "maximum disease activity" in all extramuscular systems. The distance from the mark to the left-hand boundary will be recorded. This assessment will be performed at the screening visit only to determine eligibility.

9.2.4.2 Manual Muscle Testing (MMT)

The MMT involves testing of 16 muscle groups/ motions. Each muscle group tested is scored by using Kendall grading scale, depending on how much the muscle group can do in terms of moving against gravity or against applied pressure. Scores for individual muscle groups range from 0–10 on the Kendall scale, which are ordinal grading scales. The scores are summed for a total score or for subscores involving particular muscle groups (proximal, distal, axial scores)[61].

9.3 Safety assessments

9.3.1 Laboratory assessments

Blood samples as well as samples for urinalysis will be analyzed in the local lab of the study site and will be handled and shipped in accordance with the provisions of the local lab.

Before starting the study, a list of normal ranges and units of measurement and also laboratory certificates will be provided to the Sponsor.

All laboratory values will be judged in relation to the reference ranges from the respective laboratory and signed off by an Investigator in a timely manner. Laboratory values outside the normal range and considered clinically relevant must be documented as an AE.

9.3.1.1 Safety laboratory

The following measurements will be done at the study visits as outlined in the Assessment Schedule.

- Hematology: Full blood count (FBC) with differential white blood cell count (WBC).
- Clinical chemistry:
    - Basic: sodium, potassium, chloride, creatinine (incl. eGFR), urea, total bilirubin, ALT [SGPT], gamma-glutamyl transferase [GGT], LDH, CRP, CK, Glucose
- Coagulation:
    - Basic: PTT, INR
- Additional safety parameter (will be examined from visit 4 through visit 10) : Serum IL-6, procalcitonin, ferritin

After administration of IMP, it is recommended to take samples daily if a subject suffers of fever, CRS and/or neurotoxicity until resolution.

9.3.1.2 Study-specific laboratory

- Clinical chemistry (extended): Consists of “clinical chemistry basic” plus: calcium, alkaline phosphatase, triglycerides, cholesterol, AST [SGOT], total protein, albumin, uric acid, NT-pro-BNP
- Lymphocyte subset analysis (CD3, CD4+ and CD8+ T cells, B cells, NK-cells incl. CD3 counts
- Coagulation (extended): Consists of “coagulation basic” plus: Fibrinogen, antithrombin III, d-dimers
- Immunology:
    - SLE: C3/C4, RF, ACPA, anti-dsDNA (ELISA and Crithidia test), ANA, ENA profile, anti-cardiolipin IgG/M, anti-ß2-glycoprotein IgG/M,
    - SSC: RF, ACPA, ANA, ENA profile, system sclerosis profile,
    - DM: RF, ACPA, ANA, ENA profile, myositis profile.
- ESR
- Urine analysis: Specific gravity, pH, glucose, protein, ketones, leukocytes, erythrocytes, protein quantitative and protein/creatinine ratio

- CMV-DNA and Beta-D-Glukan will be assessed on visit 4 prior to IMP administration and on day 7 – visit 7.

9.3.1.3 Other testings

Hepatitis B, hepatitis C and HIV1/2 serology testing will be performed at the screening visit. In addition, **tuberculosis** screen will be done using QuantiFERON TB-Gold.

Additionally, **TSH basal** will be tested at screening visit.

Testing of serum antibody levels of **tetanus**, **measles**, **SARS-CoV-2** and **EBV** will be performed at screening as well as visit 14 – week 24.

Serum collection for a **pregnancy test** (only for female subjects of childbearing potential) will be performed at the screening visit and prior to lymphodepletion.

SARS-CoV-2 PCR test is performed as part of the inpatient admission for visit 2.

Additionally, from subjects with moderate impairment of renal function **eGFR** will be monitored during lymphodepletion with fludarabine according to hospital routine (at least once daily).

9.3.2 B cell Depletion and Ig Levels

Circulating B cell numbers (analyzed together with FBC and lymphocyte subsets) and the immune status of IgM and IgG levels (blood serum) and the IgG subclasses in the peripheral blood will be assessed in order to determine the effect of CAR T cell therapy on humoral immunity.

The following lymphocyte subsets will be determined: CD3+, CD4+ and CD8+ T cells, CD19+ B cells, NK-cells in absolute numbers per microliter.

Ig serum levels (IgG, IgM, IgA and IgG subclasses) will be measured pre lymphodepletion (V3) and on day 28, week 8, week 12, week 16 and week 24.

B cell depletion will be measured pre-dose on day 0, on days 7, 10, 14 and 28 as well as on week 8, 12 and 24 after MB-CART19.1 infusion.

These assessments will be performed by the respective certified local clinical laboratory. The analysis will be performed according to the local instructions and guidelines.

9.3.3 Clinical safety

9.3.3.1 Vital signs

Heart rate, blood pressure (systolic and diastolic) and temperature will be measured at all visits (V1-V14) in the sitting or supine position after at least 5 minutes rest. As far as possible measurement will always be performed on the same arm.

9.3.3.2 Lung function

At the screening visit, pulmonary and respiratory parameters will be measured in all subjects.

At V14, Forced Vital Capacity (FVC), Total Lung Capacity (TLC) and DLCO (diffusion capacity) will be determined in SSc and DM/PM with pulmonary involvement only.

9.3.3.3 Brain MRI

MRI of brain will be performed at the screening visit.

9.3.3.4 ECG and echocardiography

At the screening visit, a sonogram of the heart will be assessed in supine position after at least 10 minutes rest.

In addition, a 12-lead ECG will be recorded in supine position after at least 10 minutes rest. The interpretation of the ECG will be recorded as normal, abnormal not clinically significant, or abnormal clinically significant. If the ECG is considered abnormal, the abnormality must be recorded in the CRF.

The 12-lead ECGs will be plotted with a paper speed of 25 mm/sec and 10 mm/mV amplitude, with 10 seconds recording duration for all leads and at least 3 complexes but preferably 5 complexes in each lead.

On V4 (Day 0) from start until end of IMP administration a continuous ECG has to monitor heart function.

9.3.4 Physical examination

A detailed physical examination will be performed at all visit (V1-V14) and should cover the evaluation of the skin of the whole body, nasal cavities, eyes, ears, and the respiratory,

cardiovascular, gastrointestinal, neurological, lymphatic, and musculoskeletal system. Height will be measured at V1, and weight at V1 and V2 only.

9.3.5 *Persistence and phenotyping of infused MB-CART19.1*

Blood samples for determination of persistence/phenotyping of infused MB-CART19.1 will be taken on the day prior to start of lymphodepletion (day -5), days 0 (pre-dose), 2, 7, 10, 14 and 28 as well as on weeks 8, 12 and 24.

Fresh blood samples will be sent to a central laboratory for immediate analysis.

9.3.6 *Physical function*

Disease specific objective (and subjective) physical function will be monitored over the course of the study at V1, V2 (only SSc and DM/PM), D28, and weeks 12 and 24.

SLE:
Physical function will be assessed by accelerometry (GeneActive, Activinsights, Kimbolton, UK). Patients will wear the wearable on their wrist or ankle for 7 days (24h/day). Start day is the visit day. Patients will send the wearable back to the clinic by a pre-prepared parcel. The wearable is waterproof and the battery life time is long enough to cover the full collection period of 7 days. The device is CE certified (Class I self-certified medical device under the MDD 93/42/EEC for CE Marking). Introduction to the device and procedure will take 5 minutes.

SSc:
Objective Hand Function: Isometric grip strength will be measured in pounds (lbs) using a hand dynamometer (Lafayette Instrument, Lafayette, IN, USA). Subjects will perform the test while standing, shoulder and elbow are relaxed. After a practice trial for each hand, both hands will be measured alternately for three times. The maximal force will be included in the analysis The Moberg-Picking-Up test (MPUT) will be used to measure fine motor skills [66]. The time (s) needed to move 12 small items from the table into a box with open eyes will be recorded twice with each hand.

Subjective Hand Function: Self-reported hand function will be determined using the short form of the Michigan Hand Questionnaire (sMHQ) [67, 68].

The hand function assessments will take 10 minutes.

DM/PM:

Patients will perform the 2-minute-walk-test in the hospital hall way at a self-selected fast speed while wearing an inertial sensor-based gait monitoring system (Mobile GaitLab, Portabiles HealthCare Technologies, Erlangen, Germany). Spatio-temporal gait parameters (stance time, swing time, stride time, stride length, step length, number of steps, walking speed) and distance walked in 2 minutes will be recorded. The device is CE certified (Class I medical device regulation). The walk test will take 10 minutes.

9.4 Complementary research program

Sample collection for complementary research program is optional and will only be performed in subjects who expressively have given their consent in writing after they have been informed about the details of this procedure by the Investigator. Sampling and storage instructions will be provided in separate documents. The Investigator will record all samples taken in a sample log in the investigator site file. The samples will be transferred into a local biobank, otherwise they will be destroyed after completion of the analyses.

The following analyses are planned:

- Serum levels of BAFF, type I interferon, IFN-gamma, IL-6, TNF-alpha
- Circulating T lymphocyte subsets
- T cell and B cell receptor sequencing
- Single cell RNA sequencing including IFN signature
- Exploratory biomaterial: stool probe (microbiome), serum and EDTA blood

9.5 Total amount of blood

The total amount of blood to be collected during the active part (until Week 24, V14) of the study will be approximately 705 mL (Table 3).

Collected blood will be used for local assessments of safety parameters as well as for central assessment of persistence and immunogenicity of infused MB-CART19.1 cells as well as for complementary research in central laboratory.

Table 3: Total amount of blood per visit.

	**Total volume of blood collected [ml]**

Phase	Study Day/ Month/ Year	Visit	Routine and Safety blood	MB-CART19.1 persistence	Immunogenicity	Complementary research program	Max. blood vol.
Screening	D-45 to D-15	V1	40	0	10	30	**80**
Leukapheresis	D-13	V2	15	0	0	0	**15**
Lymphodepletion	D-5 to D-3	V3	20	10	0	30	**60**
Treatment	D0, 0h	V4	20	10	10	30	**70**
	D0, 4h	V4	15	0	0	0	**15**
Short-term FU	D1	V5	15	0	0	0	**15**
	D2	V6	15	10	0	0	**25**
	D7	V7	20	10	10	0	**40**
	D10	V8	15	10	10	0	**35**
	D14	V9	20	10	10	0	**40**
	D28	V10	20	10	10	30	**70**
Mid-term FU	W8	V11	20	10	10	30	**70**
	W12	V12	20	10	10	30	**70**
	W16	V13	20	0	10	0	**30**
	W24	V14	20	10	10	30	**70**

10 SAFETY ASSESSMENTS

10.1 Adverse Events

10.1.1 Definition of Adverse Events

Adverse Event (AE) “means any untoward occurrence in a study subject administered an investigational medicinal product and which does not necessarily have a causal relationship with this treatment.” [GCP-Ordinance, 4. August 2004, § 3 (6)]

Thus, an AE can be any unfavourable and unintended sign, symptom, or disease (including intercurrent illness), deterioration of a pre-existing illness, accident, any suspected drug reaction, or a clinically relevant change of laboratory values whether or not considered related to the IMP(s).

Adverse Reaction (AR) “means all untoward and unintended responses to an investigational medicinal product unrelated to the dose administered.” [GCP-Ordinance, 4. August 2004, § 3 (7)]

The definition implies a reasonable possibility of a causal relationship between the event and the IMP(s). It covers also medication errors and uses outside what is foreseen in the protocol, including misuse and abuse of the product.

Unexpected Adverse Reaction (UAR) “means an adverse reaction, the nature or severity of which is not consistent with the available information concerning the investigational medicinal product.” [GCP-Ordinance, 4. August 2004, § 3 (9)]

10.1.2 Reporting of Adverse Events

The Investigator is responsible to evaluate and document all AEs that arise from the scheduled visit assessment after obtaining the informed consent until the end of week 12.

Pre-existing conditions that do not worsen during the course of the study are not reportable as AEs. To determine whether a condition has worsened, it is compared to the condition of the subject at the screening visit. Abnormal laboratory values obtained during the subject’s screening period will only meet AE criteria if newly detected.

Elective treatments planned before screening and which are documented in the subject’s source data are usually not regarded as AEs.

Data pertaining to AEs will be collected during each study visit based on the subject’s spontaneous description, through Investigator inquiry, or discovered in the course of examinations done during the visit. The Investigator will assess and record any AE in detail in the subject’s file

and on the CRF AE report form. AE will be monitored until the event has resolved, any abnormal laboratory values have returned to baseline or stabilized at a level acceptable to the Investigator, until there is a satisfactory explanation for the changes observed, or until the subject is lost to follow-up.

10.1.2.1 Required information

Following information needs to be recorded in the subject's CRF and the source data sheets:

- AE diagnosis (if possible) or main symptom,
- date (and time, if relevant) of onset,
- intensity (maximum observed),
- causal relationship (IMP, lymphodepletion, other study procedures),
- serious (yes or no; see section 10.2.1).
- action(s) taken with IMP,
- outcome,
- AE leading to discontinuation of the study (yes or no).
- stop date (and time, if relevant).

10.1.2.2 Intensity

The clinical intensity of an AE will be classified as:

- Mild: Signs and symptoms that can be easily tolerated. Symptoms can be ignored and disappear when the subject is distracted.
- Moderate: Signs and symptoms that cause discomfort and interfere with normal functioning, but are tolerable. They cannot be ignored and do not disappear when the subject is distracted.
- Severe: Signs and symptoms that affect usual daily activity and incapacitate the subject, thereby interrupting his/her daily activities.
- Life-threatening: Signs and symptoms that require urgent intervention.

- Fatal: Death related to AE.

The definitions above are difficult to apply to some data (e.g., clinically relevant laboratory values). In such situations, the Investigator should make a judgment based on personal experience.

10.1.2.3 Causal relationship

Investigators will be asked to assess causality as 'IMP-related', 'related to lymphodepleting therapy', 'related to any other study procedure' or 'other'.

Causality will then be categorized by using a simple binary decision for causality according to the following criteria for all three parameters as applicable:

- Not related: An AE for which there is no reasonable possibility of a causal relationship to the IMP or to the lymphodepleting therapy or any other study procedure.
- Related: An AE for which there is a reasonable possibility of a causal relationship with the IMP or with the lymphodepleting therapy or any other study procedure. This means that there are facts (evidences) or arguments to suggest a causal relationship.

The Investigator usually assesses whether there is a reasonable possibility of a causal relationship. The Sponsor should not downgrade the causality assessment given by the Investigator. If the Sponsor disagrees with the Investigator's causality assessment, the opinion of both, the Investigator and the Sponsor, should be provided with the report.

The following points should be considered during causality assessment of adverse events:

- timing of the event between administration of the drug and the onset of the adverse event,
- drug levels and evidence, if any, of overdose.

10.1.2.4 Expectedness

The Sponsor determines the expectedness of an SAR in the Reference Safety Information (RSI). This should be done from the perspective of events previously observed, not on the basis of what might be anticipated from the pharmacological properties of a medicinal product.

10.1.2.5 Actions taken

Action(s) taken with the IMP are as follows:

- drug withdrawn,
- dose reduced,
- dose increased,
- dose not changed,
- unknown,
- not applicable,
- drug withdrawn temporarily.

10.1.2.6 Outcome

The reportable outcomes and/or sequelae of an AE are as follows:

- recovered/resolved,
- recovering/resolving,
- not recovered/not resolved,
- recovered/resolved with sequelae,
- fatal,
- unknown.

If there is more than one AE, only the AE leading to death will be attributed with a "fatal" outcome.

10.2 Serious Adverse Events

10.2.1 Definition of Serious Adverse Events

A Serious Adverse Event (SAE) is any untoward medical occurrence, at any dose, that:

- results in death,
- is life-threatening[21],

[21] An adverse event is life-threatening if the patient was at immediate risk of death from the event as it occurred; i.e., it does not include a reaction that if it had occurred in a more serious form it might have caused death. For example,

- requires inpatient hospitalization, or prolongation of existing hospitalization,
- results in persistent or significant disability/incapacity[22],
- is a congenital anomaly/birth defect,
- consists of any other medically important condition.

In addition, medical and scientific judgment should be exercised in deciding whether other conditions should also be considered serious, such as important medical events that may not be immediately life-threatening or result in death or hospitalization but may jeopardize the subject's safety or may require intervention to prevent one of the other outcomes listed in the definition above. These should also be considered serious.

Examples for events also to be considered serious are:

- intensive treatment in an emergency room or at home for allergic bronchospasm or
- blood dyscrasias or convulsions that do not result in hospitalization.

Events not considered to be SAEs are hospitalizations for:

- A standard procedure for protocol therapy administration (IMP administration and routine FU until day 10). However, hospitalization or prolonged hospitalization for a complication of therapy administration will be reported as an SAE.
- Routine treatment or monitoring of the studied indication not associated with any deterioration in condition.
- A procedure for protocol/disease-related investigations (e.g., inpatient treatment for leukapheresis, lymphodepletion, scans, endoscopy, sampling for laboratory tests, bone marrow sampling). However, hospitalization or prolonged hospitalization for a complication of such procedures remains a reportable SAE.
- Hospitalization or prolongation of hospitalization for technical, practical, or social reasons, in absence of an AE.

---

drug induced hepatitis that resolved without evidence of hepatic failure would not be considered life-threatening even though drug induced hepatitis could be fatal.

[22] An adverse event is incapacitating or disabling if the event results in a substantial and/or permanent disruption of the patient's ability to carry out normal life functions.

- A procedure that is planned (i.e., planned prior to starting of treatment on study); must be documented in the source document and the CRF. Hospitalization or prolonged hospitalization for a complication remains a reportable SAE.
- An elective treatment of a pre-existing condition unrelated to the studied indication.
- Emergency outpatient treatment or observation that does not result in admission, unless fulfilling other seriousness criteria above.

A **Serious Adverse Reaction (SAR)** during the clinical study is an AE, which is at least possibly related to the IMP and fulfils at least one of the criteria of seriousness listed above.

A **Suspected Unexpected Serious Adverse Reaction (SUSAR)** during the clinical study is an AE which is at least possibly related to the IMP(s), fulfils at least one of the criteria of seriousness listed above and is not expected.

10.2.2 Reporting of Serious Adverse Events

All SAEs must be documented in the CRF and reported to Pharmacovigilance (PV; CCS Erlangen) **within 24 hours of obtaining knowledge of the events** until the end of week 24 or longer if an SAE is considered related to the IMP (SAR). SAE report forms are provided in the ISF.

Although not all information required for completion of an SAE report form may be available within the specified time period, an initial report should be submitted if the following minimal information is available:

- an identifiable subject (subject ID),
- a suspected product (if still applicable),
- an identifiable reporting source (Investigator/study site identification),
- an event or outcome that can be identified as serious.

The address for SAE reporting is:

Universitätsklinikum Erlangen

Center for Clinical Studies (CCS Erlangen)

Krankenhausstraße 12

D-91054 Erlangen

Phone: +49-9131-85-47023/ +49-9131-85-47047

Fax: +49-9131-85-35120

Email: ams.ccs@uk-erlangen.de

CSS will inform the Sponsor of all SAEs and related information immediately (within 24 hours) after receipt and request a second assessment of all SAEs by the Sponsor.

The Sponsor contact details are:

Prof. Dr. Georg Schett (Sponsor Designee)

Universitätsklinikum Erlangen, Medizinische Klinik 3

Rheumatology & Immunology

Phone: +49-9131-85-33418

Fax: +49-9131-85-34770

EMail: georg.schett@uk-erlangen.de

The Investigator must supply further supporting information within 3 days of knowledge of the SAE, and a detailed SAE description is an integral part of this supporting information. Follow-up reports should be sent immediately to the CCS Erlangen as an SAE report form (marked as a "follow-up" report) and accompanied by appropriate supporting documentation (e.g., hospital reports). The SAE has to be followed up until a final outcome and date are available.

10.2.3 Sponsor´s assessment of Serious Adverse Events

The Sponsor is responsible for the reassessment of seriousness and relatedness of all SAEs and the assessment of expectedness of all SARs.

The SAE Management of the CCS Erlangen is commissioned by the Sponsor to ensure the timely reporting of all relevant SUSARs according the applicable regulatory requirements within the following time limits:

A SUSAR that is fatal or life-threatening must be reported as soon as possible, in any case no later than 7 calendar days after the Sponsor or one of the Sponsor's delegates first becomes aware of it. Follow-up information must be reported within additional eight days.

All other SUSARs must be reported as soon as possible, in any case no later than 15 calendar days after the Sponsor or one of the Sponsor’s delegates first becomes aware of it.

During this study, there will be standard pharmacovigilance monitoring for SARs and SUSARs to allow for annual safety reporting procedures. CCS Erlangen will be responsible for safety updates to regulatory authorities in accordance with guidelines and guidance and the German Drug Law (AMG) and for updating ECs (Ethics Committees) in accordance with the guidelines.

10.3 Adverse Events of Special Interest

Adverse Events of Special Interest (AESIs) are defined as:

- flare of the underlying disease,
- Cytokine Release Syndrome (CRS): signs of CRS include fever, fatigue, loss of appetite, muscle and joint pain, nausea, vomiting, diarrhea, rashes, fast breathing, rapid heartbeat, low blood pressure,
- CAR T cell Associated Neurotoxicity Syndrome (ICANS): signs of ICANS include headache, seizures, delirium, anxiety, tremor and impaired writing ability, aphasia, decreased consciousness.
- organ toxicity (cardiac, dermatologic, gastrointestinal, hepatic, pulmonary, renal/genitourinary, or neurologic) ≥ grade 3, which is not pre-existing and not due to the underlying condition and occurs within 30 days of cell infusion and does not resolve to grade 2 within 7 days or
- ≥ grade 3 clinically relevant neutropenia or thrombocytopenia lasting ≥ 28 days from the time of infusion

All AESI have to be documented in CRF and reported to PV irrespective of their relatedness to IMP until week 24. The reporting procedure is the same as for SAEs as in section 10.2.2.

10.4 Toxicities related to lymphodepleting therapy

Therapy-related toxicities during the conditioning period will have to be rated as known and unknown therapy-related toxicity. Known therapy-related toxicities are to be found in the respective Summary of Product Characteristics (SmPCs) of the used chemotherapeutic agents. Unknown therapy-related toxicities will have to be documented as AEs as described above.

From Day 1 onwards no further distinction between known and unknown therapy-related toxicities will be made. All have to be documented as AEs until end of week 12.

10.5 Breaking the blind

Not applicable.

10.6 Pregnancy

There are no data from the use of CAR T cells in pregnant women. Therefore, MB-CART19.1 is not recommended during pregnancy and in women of childbearing potential not using contraception.

No data on the effect of MB-CART19.1 on the developing human fetus are available. Therefore, this study does not include pregnant women. At the screening visit and before start of lymphodepleting conditioning, each female of child bearing potential will undergo an urine pregnancy test.

10.6.1 Contraception

Female subjects are considered to have no child-bearing potential if they are permanently sterile, pre-, or post-menopausal. Permanent sterilization methods include hysterectomy, bilateral salpingectomy and bilateral oophorectomy. A postmenopausal state is defined as no menses for 12 months without alternative medical cause.

Female subjects of childbearing potential (and male subjects with a partner of child-bearing potential) must agree to use a highly effective contraceptive method (Pearl index <1) starting from the time of signing the ICF and for 12 months after dosing of the IMP.

According to the “Recommendations related to contraception and pregnancy testing in clinical studies” (Clinical Study Facilitation Group, 2014-09-15) birth control methods, which may be considered as highly effective, are for example:

- combined (estrogen and progestogen containing) hormonal contraception associated with inhibition of ovulation*:
    - oral
    - intravaginal
    - transdermal

- progestogen-only hormonal contraception associated with inhibition of ovulation*:
    - oral
    - injectable
    - implantable
- intrauterine device (IUD),
- intrauterine hormone-releasing system (IUS),
- vasectomized partner (with confirmed surgical success),
- sexual abstinence (when this is consistent with the preferred and usual lifestyle of the subject).

*** Hormonal contraception may be susceptible to interaction with the IMP, which may reduce the efficacy of the contraception method.**

Male subjects, unless surgically sterile, must be using two acceptable methods for contraception (e.g. spermicide and condom) during the study and refrain from fathering a child starting from the time of signing the Informed consent until 12 months after dosing of the IMP.

10.6.2 Reporting of pregnancy

All pregnancies or suspected pregnancies occurring in either a female subject of childbearing potential or partner of childbearing potential of a male subject during the course of study must be reported to CCS Erlangen immediately by email, fax or phone and by sending a completed Pregnancy Report Form to CCS.

Subjects should be instructed to notify the Investigator if, after completion of the study, it is determined that they became pregnant or, if male, their female partner conceived. Whenever possible a pregnancy should be followed to term, any premature terminations should be reported, and the status of the mother and child should be reported to the Sponsor after delivery.

10.7 Premature termination of the study

The Sponsor, the EC and PEI have the right to terminate this clinical study at any time for reasonable medical or administrative reasons such as safety concerns based on reported data (e.g. significant occurrence of relevant toxicity or inadequate response as described in section 11.9). Unsatisfactory enrolment with respect to quantity or quality, inaccurate or incomplete data

collection, falsification of records, failure to adhere to the study protocol. Any possible premature discontinuation will have to be documented adequately with reasons being stated, and information would be issued according to local requirements (e.g. to IEC and regulatory authorities).

10.8 Development Safety Update Report (DSUR)

Once per year or if requested, the Sponsor will supply a report on the safety of study subjects with all available relevant information concerning subject safety during the reference period to the competent supreme federal authority (Annual Safety Reports, § 13 Absatz 6 GCP-V). This report will also be supplied to the responsible Ethics Committee and to the Safety Monitoring Board (SMB).

10.9 Communication Plan of Safety Data

After start of the study, the following measures will be taken by the Sponsor to ensure dissemination of safety data across all Investigators:

- SUSAR and their updates will be sent via email to all Investigators within the timelines required, i.e., within 7 or 15 days;
- conduct of regular meetings on safety topics and other clinical experiences;
- after each SMB meeting results will be communicated in above mentioned meetings and SAE line listings will be forwarded to all Investigators;
- each yearly IB version update will also contain an updated section “Reference Safety Information” summarizing all SARs that have been reported as SUSARs initially.

11.1 Adherence to protocol

The Investigator should not deviate from the protocol. In medical emergencies, the Investigator may use his/her medical judgment and may remove a study participant from immediate hazard before notifying the IRB/IEC in writing regarding the type of emergency and the course of action taken.

11.2 Local data and biobank (TARDA = Translational Arthritis Research Database)

The collection of data and biomaterials during this study takes place in the mentioned facilities of Universitätsklinikum Erlangen and is stored within the Database (Ethics Approval 334_18 B) of the Department of Internal Medicine 3, Rheumatology and Immunology. The data of participants will be transferred from the CRF to the data and human biobank TARDA for storage and statistical analysis. Biomaterials and imaging data are labelled using the TARDA software and will be stored in the facilities of the Department. The data is stored exclusively on servers of Universitätsklinikum Erlangen, which are provided, maintained and secured by the MIK (Medical Center for Information and Communication Technology). Access authorizations for the TARDA are assigned on a person-related and traceable basis by the responsible TARDA supervisor (employee of the Department of Internal Medicine 3, Rheumatology and Immunology) in consultation with the data protection officer of the Department.

11.3 Source Documentation

All data collected from a subject during the course of the clinical study should be entered and/or filed in the subject's medical record, which constitute the source data. The subject's medical record must also contain a descriptive statement on the informed consent procedure (see section 13.4.2), and a copy (!) of the signed informed consent form, whereas the original must be filed in the ISF.

If the study site is using an electronic system for documenting source data, a member of the site staff must print out the source data after each visit. The print-out must be signed and dated by a member of the site staff who can confirm the accuracy and completeness of data in the paper print-out. The monitor should also sign and date after verifying the source data. The paper print-out should be stored in the subject's medical record.

If source data information is entered retrospectively, this must be done directly on the paper print-out and should be initialed and dated. The same applies to any corrections of initial data.

If the site is using a validated computer system including audit trail with a separate access for the monitor (i.e., the monitor can only access the data of the study subjects), then no such paper print-outs are required.

Data produced by automatic devices with original print-outs (e.g. clinical laboratory test results, ECG traces, blood pressure measurements) will be attached to the subject's medical record. Investigator should sign the clinical laboratory parameters (e.g. on laboratory print-outs or electronically) and should give comments on all clinically significant abnormal values accordingly.

11.4 Case Report Form

Data collected on each subject will be recorded on a paper-based Case Record Form (CRF) in a timely manner. The Investigator is responsible for ensuring that all sections of the CRF are completed correctly, and that entries can be verified against source data. If certain data are not available or not applicable, this will be indicated as such on the appropriate space on the CRF. The study monitor will review the CRFs and check them for completeness.

For Screening Failures, the screening visit has to be completed in the CRF as far as the data have been captured. (S)AEs and the related concomitant medication have to be documented if applicable and the End of Study Visit has to be completed.

11.5 Monitoring

The Sponsor ensures that appropriate monitoring procedures are performed before, during, and after the study. Before the study is initiated all aspects of the study are reviewed with the Investigator(s) and the staff. Prior to enrolling subjects into the study, the Sponsor will review the protocol, CRFs, procedures for obtaining informed consent, record keeping, and reporting of AEs with the Investigator(s). Monitoring will include visits to the Investigator(s) and his/her staff. At each monitoring visit, the facilities, medication storage area, drug accountability, CRFs, subject's source documents, and all other study documentation will be inspected/reviewed for adherence to the protocol and GCP. The Principal Investigator will review CRFs for completion and accuracy. Accuracy will be checked by performing Source Data Verification (SDV) that is a direct comparison of the entries made into the CRF against the appropriate source documentation. Any resulting discrepancies will be reviewed with the Investigator(s) and/or his/her staff. Any

necessary corrections will be made directly to the CRFs or via Source Data Clarification Forms by the Investigator(s) and/or his/her staff. Monitoring procedures require that Informed Consent Forms (ICFs), adherence to inclusion/exclusion criteria and documentation of SAEs and the proper recording be verified. Additional monitoring activities may be outlined in a study-specific monitoring plan.

11.6 Source data verification

SDV ensures accuracy and credibility of the data obtained. During monitoring visits, reported data are reviewed with regard to being accurate, complete, and verifiable from source documents (e.g., subject files, recordings from automated instruments, x-ray films, and laboratory notes). All data reported in the CRF should be supported by source documents. The Investigator/institution will permit trial-related monitoring, audits, IEC review, and regulatory inspections, providing direct access to source data/documents.

11.7 Archiving and data retention

The Investigator will maintain adequate records for the study including CRFs, medical records, laboratory results, informed consent documents, drug dispensing and disposition records, safety reports, information regarding participants who discontinued, and other pertinent data. Essential study documents are to be retained for at least 30 years after completion or discontinuation of the study. Study documents may not be destroyed prior to the retention period and without the prior written consent of the Sponsor.

12 STATISTICAL METHODS

12.1 Statistical evaluations

Analysis will include all efficacy and safety data from the screening visit to week 24 after study start for each subject. For all recruited subjects, safety measures, clinical variables, response, and follow-up information will be analyzed according to the study protocol and a Statistical Analysis Plan (SAP). Standard descriptive statistics will be used to assess the distribution of categorical and quantitative safety outcomes. A Bryant and Day two-stage optimal design will be used and a one-sided exact binomial test for the overall response rate (ORR) will be conducted, following the intention-to-treat principle. Sensitivity analyses will be considered. Time-to-event outcomes will be analyzed using the Kaplan-Meier method. Confidence intervals will be given where appropriate. Statistical analysis will be performed using R software.

12.1.1 Study variables

12.1.2 Primary safety outcome variable

The primary safety outcome variable will be measured as the number of subjects experiencing a CRS or an ICANS within the first 4 weeks after ATMP administration.

12.1.3 Secondary outcome variable

Overall Response Rate (ORR), is defined as the ratio between the number of subjects experiencing a response at week 24 as defined in section 5.2 and the total number of enrolled subjects.

Time of persistence of CAR T cells in the peripheral blood, expansion of CAR T cell in the patient over time, time of absence of B cells in the peripheral blood and time to disappearance of autoantibodies in the serum will be measured as the time elapsed from the date of the study intervention and the date of occurrence of the events as detailed in section 5.2. In case of no events, it will be censored at week 24 or at the date of last observation.

The success of the manufacturing process is determined by the GMP certification of the product. This is granted by the fulfilment of specifications including (but not limited to) dose, strength, viability, purity, transduction-rate, absence of microbial contamination.

12.1.4 Exploratory variables

B cell receptor and T cell receptor usage (as determined by sequencing analysis of circulating B cells and T cells) in the peripheral blood at screening and week 24.

The reporting of the results will comply with the CONSORT guidelines. A comprehensive statistical analysis plan will be drafted before database lock.

12.1.5 Analysis population

The analysis will be undertaken on the full analysis set with all available data and after imputation of missing values as outlined in section 11.3. Apart from the safety and efficacy endpoints, all analyses will be descriptive/exploratory in nature. The safety and efficacy endpoints will be tested at a one-sided significance level of 5% with a power of 80%. For other analyses, when statistical tests are performed, p-values and 95% confidence intervals will be used for exploratory purposes

12.2 Determination of sample size

The safety and efficacy endpoints of this study are the incidence and grading of severity (graded 0-4) of Cytokine Release Syndrome (CRS) and of CAR T cell Associated Neurotoxicity Syndrome (ICANS) within the first 4 weeks after ATMP administration and the Overall Response Rate (ORR) at week 24. A Bryant and Day two stage optimal design is used ([69]). The null hypothesis that the true toxicity rate is 50% will be tested against a one-sided alternative of an acceptable toxicity rate of 20% or less. At the same time, a null hypothesis that the true response rate is 30% will be tested against a one-sided alternative of a response rate equal or higher than 60%.

The following design will be developed with a one-sided type I error = 0.05 (alpha) and type II error (beta) of 0.20 (power 80%):

- Stage 1: 8 subjects will be accrued with stopping rules for relevant toxicity as defined by more than 4 subjects experiencing either CRS > grade 2 or ICANS > grade 2 or organ toxicity (cardiac, dermatologic, gastrointestinal, hepatic, pulmonary, renal/genitourinary, or neurologic) ≥ grade 3, which is not pre-existing and not due to the underlying condition and occurs within 30 days of cell infusion and does not resolve to grade 2 within 7 days or ≥ grade 3 clinically relevant neutropenia or thrombocytopenia lasting ≥ 28 days from the time of infusion) or inadequate response (less than 4 subjects responding).

- Stage 2: 16 subjects will be accrued with stopping rules for relevant toxicity as defined by more than 6 subjects experiencing relevant toxicities as described above.

Power calculations were performed with R (Version 4.0.1) software.

12.3 Statistical analysis

12.3.1 Dataset to be analysed

The Available Data (AD) population will be the primary population to be analysed for safety and efficacy. Data will be analysed with and without imputation of missing data. All data will be analysed, except data after subjects have withdrawn consent. Data collected before withdrawal of consent remain in the statistical analysis.

12.3.2 Analysis of safety

Adverse events will be encoded using the MedDRA dictionary. Frequency tables for adverse events will be compiled, based on subjects experiencing an AE and based on the number of AEs. Laboratory data will be presented using descriptive statistics for continuous parameters and frequencies with respect to normal ranges. Concomitant diseases and medical history will be encoded ICD10. These data will be presented using frequency tables. Previous medication and concomitant medication will be encoded using the ATC dictionary. Frequency tables will be compiled based on the encoding for the medication, applying the full code and the first 3 digits subgroup.

12.3.3 Analysis of efficacy

We will describe the baseline characteristics, primary, and secondary outcome measures using appropriate summary statistics. These will include means and standard deviations for continuous and discrete data, and counts and percentages for categorical data. Median and interquartile ranges will also be estimated if a robust central tendency estimate is deemed necessary. Same summary statistics will also be used to describe outcomes at week 24 as appropriate. Secondary outcome data with continuous or discrete values will be summarized and, if appropriate, compared using the same methods as the primary outcome for continuous and discrete data. Longitudinal categorical outcome data will be compared using a McNemar test for baseline-follow-up comparisons; Wilcoxon signed rank test will be used for paired comparisons of quantitative items; more generally, a generalized linear model will be used to analyse outcomes along time.

Due to the exploratory nature of this study, the focus of these analyses is to obtain information on the magnitude of change with respect to specified primary and secondary endpoints including corresponding effect, which may be applied in sample size calculations of subsequent studies with similar endpoints. Estimated p values will not be corrected for multiple-testing.

12.4 Missing Data

Missing values for outcomes of interest may occur for primary as well as secondary study endpoints. Single missing items in subject questionnaires will be imputed by published instructions about how to handle missing values for these instruments. Data of subjects who were lost to follow-up after visit 4 will be imputed by data of the last visit completed (Last Observation Carried Forward technique, LOCF), implying that the status of subjects lost to follow-up would not have changed during the period of last visit completed and end of study.

13 ETHICS AND REGULATORY

13.1 Investigator obligations

The Investigator agrees to conduct the clinical study in compliance with the protocol agreed by the Sponsor and, if required by regulatory authorities, which got favorable opinion by an Ethics Committee and for which a Clinical Trial Authorization (CTA) has been granted by the Competent Authority (CA). The Investigator and the Sponsor should sign the protocol (and protocol amendments) to confirm this agreement.

The Ethics Committee of the Investigator as well as the CA must review and give approval for the clinical study as described in this protocol to be conducted in human subjects.

13.2 Ethics Committee and Competent Authority

The study will be conducted under the auspices of an IRB/IEC, as defined in local regulations, ICH-GCP, and in accordance with the ethical principles that have their origin in the Declaration of Helsinki.

Before initiating a study, the Investigator will have written and dated full approval from the responsible IRB/IEC for the protocol.

The Investigator will also promptly report to the IRB/IEC all changes in the study, all unanticipated problems involving risks to human subjects or others, to eliminate immediate hazards to subjects. The Investigator will not make any changes in the study or study conduct without IRB/IEC approval, except where necessary to eliminate apparent immediate hazards to the subjects. For minor changes to a previously approved protocol during the period covered by the original approval, it may be possible for the Investigator to obtain an expedited review by the IRB/IEC as allowed.

As part of the IRB/IEC requirements for continuing review of approved studies, the Investigator will be responsible for submitting periodic progress reports to the IRB/IEC at intervals appropriate to the degree of subject risk involved but no less than once per year. The Investigator should provide a final report to the IRB/IEC following study completion.

13.3 Patient Information and Informed Consent

The subjects will be provided with detailed information on the nature and importance of the study always from both the rheumatological (Med 3) and haemato-oncological side (Med 5). They will receive a description of the foreseeable risks and discomforts and a description of the procedures to be followed. The subjects will be informed that they are free to withdraw from the study at any time without any disadvantages. Prior to the start of the study the subjects will agree to the participation in the study by signing the informed consent form.

Voluntary written informed consent will be obtained from each participant. This informed consent has to be obtained at the screening visit prior to any study-related procedures. Each subject should be given both verbal and written information (in a language that is understandable to the subject) describing the meaning, aim and conduct of the study. This will take place under conditions where the participant has adequate time to consider the risks and benefits associated with his participation in the study. The subjects will have the possibility to ask all their questions.

The consent will be signed and dated by the subject and the Investigators (Med 3 and Med 5) who conducted the informed consent discussion.

It is the responsibility of the Investigator(s) to assure that informed consent is obtained from each participant in accordance with section 4.8 of the ICH consolidated guideline for Good Clinical Practice from July 1996, its Addendum E6 (R2) of June 2017, and local regulations. The signed informed consent will be retained with the study records. Each participant will receive a copy of the signed informed consent.

If a protocol amendment is required, the Informed Consent Form (ICF) may need to be revised and a re-consent form will be generated to reflect the changes to the protocol. Both, the revised consent form and the re-consent form must be reviewed and approved by the appropriate EC: all ongoing subjects must sign the re-consent form. Subjects should be informed in a timely manner if new information becomes available that might be relevant to the subject’s willingness to continue participation in the study. The communication of this information should be documented.

The approved revised ICF will be used to inform all subjects newly to be enrolled into the study.

The Investigator should maintain a log of all subjects who sign the ICF and indicate if the subject received study drug or, if not, the reason why. The subject’s medical records should also document that the ICF was signed and dated prior to any study-related procedures being performed.

13.4 Duration of the study

The study will be closed when all enrolled and treated subjects have completed week 24 (LSLV) or the trial has to be terminated prematurely.

13.5 Subject privacy

The subject will be informed of procedures to protect subject privacy. Although recorded data will be passed on in a coded version only to authorized individuals, re-identification by the Investigator (e.g., in case of emergencies) will be possible by the study number assigned to the subject. Access to non-coded data will be allowed solely to check validity, and such access will be limited strictly to authorized individuals (e.g., the sponsor or individuals authorized by the Sponsor, auditors, regulatory authorities, or members of IECs) who have been bound to confidentiality.

13.6 Insurance

From the beginning of the study until its termination, each subject is insured against any health impairment occurring as a result of participation in the study in accordance with the laws and regulations of the country in which the study is performed.

The subject or his/her legally acceptable representative will be informed by the investigator and through the subject's informed consent form about the existence of this insurance and the resulting obligations. The insurance conditions will be handed out to the subject or his/her legally acceptable representative, if requested or if required by local law.

Any medical deviation from the clinical study protocol that is deemed to have occurred through the subject's own fault is not covered by this insurance.

The sponsor is usually not liable for injuries/cases of death that occur solely as a consequence of the subject's underlying disease or condition, or from diagnostic or therapeutic measures not specifically required by the agreed clinical study protocol. The sponsor is also usually not liable for events resulting from negligence of the investigator, clinical study staff, and/or CRO, including failure to act according to ICH-GCP principles or to comply strictly with the agreed clinical study protocol.

13.7 Publication

Authorship of planned manuscripts for submission to medical journals shall be determined in accordance with the International Committee of Medical Journal Editors (ICMJE) Uniform Requirements for Manuscripts Submitted to Biomedical Journals.

14 APPENDICES

14.1 Grading of CRS[23]

ASTCT CRS Consensus Grading

CRS Parameter[5]	Grade 1	Grade 2	Grade 3	Grade 4
**Fever[1,2]**	Temperature ≥38°C	Temperature ≥38°C	Temperature ≥38°C	Temperature ≥38°C
		With		
**Hypotension[1]**	None	Not requiring vasopressors	Requiring one vasopressor with or without vasopressin	Requiring multiple vasopressors (excluding vasopressin)
		And/ or[3]		
**Hypoxia[1]**	None	Requiring low-flow nasal cannula[4] or blow-by	Requiring high-flow nasal cannula[4], facemask, non-rebreather mask, or Venturi mask	Requiring positive pressure (eg: CPAP, BiPAP, intubation and mechanical ventilation)

1) Not attributable to any other cause
2) In subjects who have CRS then receive tocilizumab or steroids, fever is no longer required to grade subsequent CRS severity
3) CRS grade is determined by the more severe event
4) Low-flow nasal cannula is ≤ 6 L/min and high-flow nasal cannula is > 6 L/min
5) Organ toxicities associated with CRS may be graded according to CTCAE v5.0 but they do not influence CRS grading

[23] From Lee, D.W., et al., ASTCT Consensus Grading for Cytokine Release Syndrome and Neurologic Toxicity Associated with Immune Effector Cells. Biol Blood Marrow Transplant, 2019. 25(4): p. 625-638.

14.2 Management of CRS

Grade	**Definition** (as in App 14.1)	**Measures**
1	Temperature ≥38°C<br>no hypotension,<br>no hypoxia	• Antipyretics<br>• Assess for infection using blood and urine cultures<br>• Empiric broad-spectrum antibiotics if neutropenic<br>• Maintenance intravenous (IV) fluids for hydration<br>• Symptomatic management of constitutional symptoms and organ toxicities<br>• Consider tocilizumab 8 mg/kg (≥30 kg) or 12 mg/kg (<30 kg) IV (max. 800 mg per dose) for persistent (lasting >3 days) and refractory fever
2	Temperature ≥38°C with hypotension not requiring vasopressors and/or hypoxia requiring low-flow nasal cannula or blow-by	• IV fluid bolus of normal saline: 500–1,000 ml adult subjects, 10 ml/kg pediatric subjects. Repeat if persistent hypotension.<br>• Tocilizumab 8 mg/kg (≥30 kg) or 12 mg/kg (<30 kg) IV (max. 800 mg/dose) for the management of hypotension that is refractory to two fluid boli; repeat after 6 h if needed for a max. total of 3 doses<br>• If hypotension persists after two fluid boluses and tocilizumab, consider transfer to intensive-care unit (ICU), start vasopressors, obtain echocardiogram, and initiate other methods of hemodynamic monitoring<br>• If hypotension persists after 1–2 doses of tocilizumab, dexamethasone can be used at 10 mg IV every 6 h (adult) / 0.2 mg/kg/dose IV every 6 h (pediatric)<br>• Manage fever and constitutional symptoms as in grade 1.<br>• In cases of hypoxia, apply supplemental oxygen.
3	Temperature ≥38°C with hypotension requiring multiple vasopressors and/or hypoxia requiring high-flow nasal cannula or face mask	• Transfer to ICU, obtain echocardiogram and perform hemodynamic monitoring<br>• IV fluid boluses and vasopressor as needed.<br>• Tocilizumab as recommended for grade 2 CRS<br>• In addition, dexamethasone at 10 mg IV every 6 h (adult) / 0.2 up to 0.5 mg/kg/dose (max. 10 mg/dose) IV every 6 h (pediatric)<br>• Manage fever and constitutional symptoms as indicated for grade 1 CRS<br>• In cases of hypoxia, supplemental oxygen including high-flow oxygen delivery and non-invasive positive pressure ventilation
4	Temperature ≥38°C with hypotension requiring a single vasopressor and/or hypoxia requiring positive pressure or invasive ventilation	• IV fluids, vasopressors, tocilizumab and dexamethasone as defined for the management of grade 3 CRS<br>• If unsuccessful: Methylprednisolone 1 g/day IV (adults) or 20 mg/kg/d (pediatric) for 3 days IV.<br>• Supplemental oxygen including high-flow oxygen delivery and non-invasive positive pressure ventilation or invasive ventilation

14.3 Grading of ICANS[24]

ASTCT CRS Consensus Grading

Neurotoxicity Domain[1]	Grade 1	Grade 2	Grade 3	Grade 4
**ICE Score[2]**	7-9	3-6	0-2	0 (subject is unarousable and unable to perform ICE)
**Depressed level of consciousness**	Awakens spontaneously	Awakens to voice	Awakens only to tactile stimulus	Subject is unarousable or requires vigorous or repetitive tactile stimuli to arouse or stupor or coma
**Seizure**	N/A	N/A	Any clinical seizure focal or generalized that resolves rapidly; or Non-convulsive seizures on EEG that resolve with intervention	Life-threatening prolonged seizure (>5 min); or Repetitive clinical or electrical seizures without return to baseline in between
**Motor findings**	N/A	N/A	N/A	Deep focal motor weakness such as hemiparesis or paraparesis
**Raised intracranial pressure / Cerebral edema**	N/A	N/A	Focal/local edema on neuroimaging	Diffuse cerebral edema on neuroimaging; Decerebrate or decorticate posturing; or Cranial nerve VI palsy; or Papilledema; or Cushing's triad

1) ICANS grade is determined by the most severe event (ICE score, level of consciousness, seizure, motor findings, raised ICP/cerebral edema) not attributable to any other cause
2) ICE score:
   - Orientation: orientation to year, month, city, hospital: 4 points
   - Naming: ability to name 3 objects (e.g. point to clock, pen, button): 3 points
   - Following commands: ability to follow simple commands (e.g. “Show me 2 fingers” or “Close your eyes and stick out your tongue”): 1 point
   - Writing: ability to write a standard sentence (e.g. “Our national bird is the bald eagle”): 1 point
   - Attention: ability to count backwards from 100 by 10: 1 point

[24] From Lee, D.W., et al., ASTCT Consensus Grading for Cytokine Release Syndrome and Neurologic Toxicity Associated with Immune Effector Cells. Biol Blood Marrow Transplant, 2019. 25(4): p. 625-638.

14.4 Management of ICANS

The following measures are recommended per protocol to avoid life-threatening and fatal neurotoxicity:

- Seizure prophylaxis e.g. with levetiracetam, is recommended for all subjects
- Any new onset grade 2 or higher neurotoxicity should be evaluated by an age appropriate neurological examination, brain magnetic resonance imaging, and lumbar puncture for CSF examination, as clinically indicated.
- In any case, anti-epileptics should be considered in all subjects with ≥2 grade neurotoxicity and started in all subjects with a seizure. The first choice is levetiracetam.
- In subjects with grade 3 and grade 4 neurotoxicity, Dexamethasone 0.2- 0.5 mg/kg i.v. q6h should be given (max 10 mg i.v. q6h). Administration of tocilizumab 8 mg/kg (max. 800 mg/dose) should be considered in case of concurrent CRS or in case of persistent neurotoxicity. A repeated dose should be considered if symptoms have not stabilized or improved within 12 – 24 hours.

14.5 Grading of CAR T cell specific organ toxicity and action[25]

Category	Adverse Event (AE)	Grade 1	Grade 2	Grade 3	Grade 4
Cardiac disorders	Sinus tachycardia	Asymptomatic; intervention not indicated	Symptomatic; non-urgent medical intervention indicated	Urgent medical intervention indicated	-
	Arrhythmia or block	Asymptomatic; intervention not indicated	Non-urgent medical intervention indicated	Urgent medical intervention indicated	Life-threatening consequences; urgent intervention indicated
	Decreased ejection fraction (EF)	-	EF at rest 40-50%; 10-19% decrease in baseline value	EF at rest 20-39% or ≥20% reduction of baseline value	EF at rest <20%
Respiratory disorders	Pleural effusion /pleural effusion	Asymptomatic; clinical or diagnostic observations only; intervention not indicated	Symptomatic; medical intervention indicated (diuretic administration or limited therapeutic thoracentesis)	Symptomatic with respiratory distress and hypoxia; (surgical) intervention including pleural drainage or pleurodesis indicated	Life-threatening respiratory or hemodynamic compromise; intubation or intervention urgently indicated
	Pulmonary edema	Radiological findings only, mini-male dyspnea on exertion	Moderate dyspnea on exertion, medical intervention indicated; limitation of instrumental ADL[1,2]	Severe dyspnea or dyspnea at rest; oxygen indicated; limitation of self-sustaining ADL[1,3]	Life-threatening respiratory impairment; urgent intervention or intubation with respiratory support indicated
Gastrointestinal disorders	Nausea	Loss of appetite without change in eating habits	Decreased oral intake without significant dehydration, weight loss or malnutrition	Inadequate oral caloric or fluid intake, tube feeding, total parenteral nutrition, or hospitalization indicated	-
	Vomiting / Emesis	No intervention necessary	Medical intervention necessary, administration of fluid intravenous, intervention possible on an outpatient basis	Feeding tube, complete parenteral complete parenteral nutrition or hospitalization necessary	Life-threatening consequences; Urgent intervention indicated

[25] From M5-CAR-SOP-Gradeinteilung CAR-T spezifischer Organtoxizität nach CTCAE (V5.0)

	Diarrhea	Increase <4 stools per day above normal frequency; small increase in stool volume above normal	Increase 4-6 stools per day above normal frequency; moderate increase in stool volume above normal; instrumental ADL[2] limitations	Increase ≥ 7 stools per day above normal frequency; hospitalization indicated; severe increase in stool volume above normal frequency beyond normal; restriction of self-care ADL[1,3]	Life-threatening consequences; urgent intervention indicated

Category	Adverse Event (AE)	Grade 1	Grade 2	Grade 3	Grade 4
**Hepatobiliary disorders**	AST/GOT or ALT/GPT elevated	> upper normal limit up to 3 x upper normal limit if baseline was normal; 1.5-3 x baseline if baseline was already pathological	> than 3-5 x upper limit if baseline was normal; >3-5 x baseline if baseline was pathological	> than 5-20 x upper limit of normal if baseline was normal, 5-20 x baseline if baseline was pathological	> than 20 x upper limit if baseline was normal, 20 x baseline if baseline was pathological was pathological
	Hyperbilirubinemia	> upper normal limit up to 1.5 x upper normal limit if baseline value was normal; >1-1.5x baseline value if baseline value was already pathological was pathological	> 1.5-3 x upper normal limit if baseline was normal was normal; >1.5-3x baseline value if baseline value was already pathological	> 3-10 x upper normal limit if baseline was normal was normal; >3-10 x baseline if baseline value was already pathological	> than 10 x upper normal limit if baseline was normal >10 x baseline if baseline was already pathological was pathological
**Kidney and urinary tract disorders**	Decreased urine production	-	-	Oliguria (<80 ml/8 h)	Anuria (<240 ml/24 h)
	Creatinine increase	> upper normal limit to 1.5 x upper normal limit normal limit	>1.5 x 3.0 baseline; >1.5 x 3.0 upper normal limit	>3.0 baseline; >3.0 - 6.0 x upper normal limit	>6x upper normal limit
**Coagulation disorders**	Disseminated intravascular coagulation (DIC)	-	Pathological laboratory findings without bleeding tendency	Pathological laboratory findings and bleeding tendency	Life-threatening effects; Urgent intervention indicated
**Skin disorders**	Acneiform exanthema	Papules and/or pustules < 10% of BSA, associated or not with symptoms of pruritus or tenderness	Papules and/or pustules on 10-30% of BSA, associated or not with symptoms of pruritus and tenderness; associated with psychosocial	Papules and/or pustules on >30% of BSA, associated with moderate or severe symptoms; limitation of self-care ADL[1,3] associated with	Papules and/or pustules on any % of BSA, associated or not with symptoms of pruritus and tenderness and are associated with extensive

		impact, limitation of instrumental ADL[1,2], exanthema; exanthema affecting more than 30% of BSA with/without mild symptoms	local superinfection, therapy with oral antibiotics indicated	superinfection (therapy with i.v. antibiotics indicated); life-threatening sequelae
Maculopapular exanthema	Papules and/or pustules on <10% of BSA, associated or not with symptoms (e.g. itching, burning, tension)	Papules and/or pustules on 10 to 30% BSA, associated or not associated with symptoms (e.g. itching, burning, tension); limitation of instrumental ADL[1, 2], exanthema with >30% BSA with max. mild symptoms	papules and/or pustules on > 30% of the BSA, associated with moderate or severe symptoms; restriction of self-care ADL[1,3]	-

1) Activities of daily living (ADL)
2) Instrumental ADL: Preparing meals, shopping for food or clothes, using the telephone, handling money, etc.
3) Self-care ADL: Bathing, dressing and undressing, eating independently, using the toilet, taking medication and not being bedridden.

Action	Grade 1	Grade 2	Grade 3	Grade 4
• **Symptom management according to general guidelines**<br>• **Consultation with senior physician in case of symptom persistence**	spontaneous resolving without special therapeutic countermeasures	outpatient treatment with simple drugs (for example, by means of peripherally acting analgesics, steroids, oral antibiotics)	hospitalization for the initiation of intensive medication and supportive measures (for example, centrally acting analgesics, intravenous administration of antibiotics, insertion of a percutaneous endoscopically placed gastrostomy [PEG])	immediate emergency hospitalization, immediate intensive medical measures or surgical interventions

14.6 Patient's and Physician's Global Assessment (PtGA/ PhGA) of disease activity

Patientenurteil zur Krankheitsaktivität

- Wie aktiv ist Ihre Erkrankung in den letzten 7 Tagen gewesen?

Keine Aktivität	Stärkst mögliche Aktivität	___ ___ ___ (0-100 mm)

Arzturteil zur Krankheitsaktivität

Keine Aktivität	Stärkst mögliche Aktivität	___ ___ ___ (0-100 mm)

14.7 Health Assessment Questionnaire-Disability Index (HAQ-DI)

Bitte kreuzen Sie die Antwort an, die am besten Ihre Fähigkeiten **während der letzten Woche beschreibt.**

**1. Ankleiden und Körperpflege**	OHNE jede Schwierig-keit	Mit EINIGEN Schwierig-keiten	Mit GROSSEN Schwierig-keiten	Nicht dazu in der Lage
Können Sie sich ankleiden, inkl. Binden von Schnürsenkeln und Schließen von Knöpfen?	☐	☐	☐	☐
Können Sie sich die Haare waschen?	☐	☐	☐	☐
**2. Aufstehen**				
Können Sie von einem Stuhl ohne Armlehnen aufstehen?	☐	☐	☐	☐
Können Sie sich ins Bett legen und wieder aufstehen?	☐	☐	☐	☐
**3. Essen & Trinken**				
Können Sie Fleisch schneiden?	☐	☐	☐	☐
Können Sie eine volle Tasse oder ein volles Glas zum Mund führen?	☐	☐	☐	☐
Können Sie eine neue Milchtüte öffnen?	☐	☐	☐	☐
**4. Gehen**				
Können Sie im Freien auf ebenem Gelände gehen?	☐	☐	☐	☐
Können Sie fünf Treppenstufen hinaufsteigen?	☐	☐	☐	☐

Bitte kreuzen Sie alle **Hilfsmittel** an, die Sie üblicherweise benutzen:

☐ Stock	☐ Spezialstuhl oder erhöhter Stuhl
☐ Gehwagen	☐ Besondere Hilfsmittel beim Essen und Trinken
☐ Krücken	☐ Hilfsmittel zum Ankleiden (langer Schuhlöffel, Knöpfe, Strümpfe anziehen, usw.)
☐ Rollstuhl	☐ Andere, bitte angeben: ______________________

Bitte kreuzen Sie die Bereiche an, bei denen Sie gewöhnlich **Hilfe von einem anderen Menschen** benötigen:

☐ Ankleiden & Körperpflege	☐ Essen & Trinken
☐ Aufstehen	☐ Gehen

**5. Hygiene**	OHNE jede Schwierig-keit	Mit EINIGEN Schwierig-keiten	Mit GROSSEN Schwierig-keiten	Nicht dazu in der Lage
Können Sie Ihren Körper von Kopf bis Fuß waschen und abtrocknen?	☐	☐	☐	☐
Können Sie in der Badewanne baden?	☐	☐	☐	☐
Können Sie sich auf die Toilette setzen und wieder aufstehen?	☐	☐	☐	☐

6. Erreichen von Gegenständen

Können Sie sich strecken und einen etwa 2kg schweren Gegenstand (z.B. einen Sack Kartoffeln) von einem knapp über Ihrem Kopf befindlichen Regal herunterholen?	☐	☐	☐	☐
Können Sie sich bücken, um Kleidungsstücke vom Boden aufzuheben?	☐	☐	☐	☐

7. Greifen

Können Sie Autotüren öffnen?	☐	☐	☐	☐
Können Sie Gläser mit Schraubverschluss öffnen, die vorher schon einmal geöffnet waren?	☐	☐	☐	☐
Können Sie Wasserhähne auf- und zudrehen?	☐	☐	☐	☐

8. Andere Tätigkeiten

Können Sie Besorgungen machen und einkaufen?	☐	☐	☐	☐
Können Sie in ein Auto ein- und aussteigen?	☐	☐	☐	☐
Können Sie Hausarbeiten verrichten, z.B. Staubsaugen und Gartenarbeit?	☐	☐	☐	☐

Bitte kreuzen Sie die **Hilfsgeräte** an, die Sie üblicherweise für diese Tätigkeiten benutzen:

☐ erhöhter Toilettensitz	☐ Geräte mit langen stielen im Badezimmer
☐ Badewannensitz	☐ Geräte mit langen Stielen zum Erreichen von Gegenständen
☐ Schraubverschluss-Öffner für Gläser (die vorher schon geöffnet waren)	☐ Andere, bitte angeben: ____________
☐ Badewannenstange bzw. griff	

Bitte kreuzen Sie die Bereiche an, bei denen Sie gewöhnlich die **Hilfe von einem anderen Menschen** benötigen:

☐ Hygiene	☐ Greifen
☐ Erreichen von Gegenständen	☐ Andere Tätigkeiten

14.8 Functional Assessment of Chronic Illness Therapy – Fatigue (FACIT Fatigue)

Nachfolgend finden Sie eine Liste von Aussagen, die von anderen Personen mit Ihrer Krankheit für wichtig befunden wurden. **Bitte geben Sie jeweils and, wie sehr jede der Aussagen im Laufe der letzten 7 Tage auf Sie zugetroffen hat, indem Sie die entsprechende Zahl markieren.**

		**Über-haupt nicht**	**Ein wenig**	**Mäßig**	**Ziemlich**	**Sehr**
1.	Ich bin erschöpft.	☐	☐	☐	☐	☐
2.	Ich fühle mich insgesamt sehr schwach.	☐	☐	☐	☐	☐
3.	Ich fühle mich lustlos (ausgelaugt).	☐	☐	☐	☐	☐
4.	Ich bin müde.	☐	☐	☐	☐	☐
5.	Es fällt mir schwer, etwas anzufangen, weil ich müde bin.	☐	☐	☐	☐	☐
6.	Es fällt mir schwer, etwas zu Ende zu führen, weil ich müde bin.	☐	☐	☐	☐	☐
7.	Ich habe Energie.	☐	☐	☐	☐	☐
8.	Ich bin in der Lage, meinen gewohnten Aktivitäten nachzugehen. (Beruf, Einkaufen, Schule, Freizeit, Sport, usw.)	☐	☐	☐	☐	☐
9.	Ich habe das Bedürfnis, tagsüber zu schlafen.	☐	☐	☐	☐	☐
10.	Ich bin zu müde, um zu essen.	☐	☐	☐	☐	☐
11.	Ich brauche Hilfe bei meinen gewohnten Aktivitäten. (Beruf, Einkaufen, Schule, Freizeit, Sport, usw.)	☐	☐	☐	☐	☐
12.	Ich bin frustriert, weil ich zu müde bin, die Dinge zu tun, die ich machen möchte.	☐	☐	☐	☐	☐
13.	Ich musste meine sozialen Aktivitäten einschränken, weil ich müde bin.	☐	☐	☐	☐	☐

14.9 QLQ-C30

GERMAN

EORTC QLQ-C30 (Version 3)

Wir sind an einigen Angaben interessiert, die Sie und Ihre Gesundheit betreffen. Bitte beantworten Sie die folgenden Fragen selbst, indem Sie die Zahl einkreisen, die am besten auf Sie zutrifft. Es gibt keine „richtigen" oder „falschen" Antworten. Ihre Angaben werden streng vertraulich behandelt.

Bitte tragen Sie Ihre Initialen ein: |_|_|_|_|
Ihr Geburtsdatum (Tag, Monat, Jahr): |_|_|_|_|_|_|_|_|
Das heutige Datum (Tag, Monat, Jahr): 31 |_|_|_|_|_|_|_|_|

	Überhaupt nicht	Wenig	Ziemlich	Sehr
1. Bereitet es Ihnen Schwierigkeiten, sich körperlich anzustrengen (z. B. eine schwere Einkaufstasche oder einen Koffer zu tragen)?	1	2	3	4
2. Bereitet es Ihnen Schwierigkeiten, einen <u>längeren</u> Spaziergang zu machen?	1	2	3	4
3. Bereitet es Ihnen Schwierigkeiten, eine <u>kurze</u> Strecke außer Haus zu gehen?	1	2	3	4
4. Müssen Sie tagsüber im Bett liegen oder in einem Sessel sitzen?	1	2	3	4
5. Brauchen Sie Hilfe beim Essen, Anziehen, Waschen oder Benutzen der Toilette?	1	2	3	4

**Während der letzten Woche:**	Überhaupt nicht	Wenig	Ziemlich	Sehr
6. Waren Sie bei Ihrer Arbeit oder bei anderen tagtäglichen Beschäftigungen eingeschränkt?	1	2	3	4
7. Waren Sie bei Ihren Hobbys oder anderen Freizeitbeschäftigungen eingeschränkt?	1	2	3	4
8. Waren Sie kurzatmig?	1	2	3	4
9. Hatten Sie Schmerzen?	1	2	3	4
10. Mussten Sie sich ausruhen?	1	2	3	4
11. Hatten Sie Schlafstörungen?	1	2	3	4
12. Fühlten Sie sich schwach?	1	2	3	4
13. Hatten Sie Appetitmangel?	1	2	3	4
14. War Ihnen übel?	1	2	3	4
15. Haben Sie erbrochen?	1	2	3	4
16. Hatten Sie Verstopfung?	1	2	3	4

<u>Bitte wenden</u>

**Während der letzten Woche:**	**Überhaupt nicht**	**Wenig**	**Ziemlich**	**Sehr**
17. Hatten Sie Durchfall?	1	2	3	4
18. Waren Sie müde?	1	2	3	4
19. Fühlten Sie sich durch Schmerzen in Ihrem alltäglichen Leben beeinträchtigt?	1	2	3	4
20. Hatten Sie Schwierigkeiten, sich auf etwas zu konzentrieren, z. B. auf Zeitunglesen oder Fernsehen?	1	2	3	4
21. Fühlten Sie sich angespannt?	1	2	3	4
22. Haben Sie sich Sorgen gemacht?	1	2	3	4
23. Waren Sie reizbar?	1	2	3	4
24. Fühlten Sie sich niedergeschlagen?	1	2	3	4
25. Hatten Sie Schwierigkeiten, sich an Dinge zu erinnern?	1	2	3	4
26. Hat Ihr körperlicher Zustand oder Ihre medizinische Behandlung Ihr Familienleben beeinträchtigt?	1	2	3	4
27. Hat Ihr körperlicher Zustand oder Ihre medizinische Behandlung Ihr Zusammensein oder Ihre gemeinsamen Unternehmungen mit anderen Menschen beeinträchtigt?	1	2	3	4
28. Hat Ihr körperlicher Zustand oder Ihre medizinische Behandlung für Sie finanzielle Schwierigkeiten mit sich gebracht?	1	2	3	4

Bitte kreisen Sie bei den folgenden Fragen die Zahl zwischen 1 und 7 ein, die am besten auf Sie zutrifft:

29. Wie würden Sie insgesamt Ihren Gesundheitszustand während der letzten Woche einschätzen?

1	2	3	4	5	6	7
sehr schlecht						ausgezeichnet

30. Wie würden Sie insgesamt Ihre Lebensqualität während der letzten Woche einschätzen?

1	2	3	4	5	6	7
sehr schlecht						ausgezeichnet

© QLQ-C30 Copyright 1995 EORTC Quality of Life Group. Alle Rechte vorbehalten. Version 3.0

14.10 British Isles Lupus Assessment Group (BILAG) 2004 index[26]

Key	
(blue)	A
(pink)	B
(yellow)	C
All	D previous
All	E never

System	ITEM	ABBREVIATED GLOSSARY	Not Present	Improving	Same	Worse	New	SCORE A - E
Mucocutaneous	Skin eruption - ***severe***	Any lupus rash except panniculitis, bullous lesion and angio-oedema. Must involve >18% BSA	0	1	2	3	4	
Mucocutaneous	Skin eruption - ***mild***	As above but <18% BSA. If malar rash: observed by doctor and present at least 1/52	0	1	2	3	4	
Mucocutaneous	Mucosal ulceration - ***severe***	Disabling (sig interfering with oral intake), extensive & deep. Observed by a physician.	0	1	2	3	4	
Mucocutaneous	Mucosal ulceration - ***mild***	Localized and/or non-disabling ulceration	0	1	2	3	4	
Mucocutaneous	Alopecia - ***severe***	Clinically detectable (diffuse or patchy) hair loss with scalp inflammation (redness over scalp)	0	1	2	3	4	
Mucocutaneous	Alopecia - ***mild***	Diffuse or patchy hair loss without scalp inflammation (clinically detectable /by history)	0	1	2	3	4	
Mucocutaneous	Digital infarct / nodular vasculitis	Localised single / multiple infarct(s) over digit(s) or tender erythematous nodule(s)	0	1	2	3	4	
Mucocutaneous	Periungual erythema / chilblains	Chilblains = localised inflammatory lesions (may ulcerate) precipitated by exposure to cold	0	1	2	3	4	
Mucocutaneous	Other features	Angio-oedema, panniculitis, cutaneous vasculitis, or splinter haemorrhages	No	YES → NEXT PAGE				
MSK	Arthritis - ***severe***	Observed Synovitis ≥2 joints + marked loss of ROM & ADL, on several days (cum) in past 4/52.	0	1	2	3	4	
MSK	Arthritis ***(moderate)***, tendonitis or tenosynovitis	≥1 joint (observed or history), some loss ROM, several days of past 4/52	0	1	2	3	4	
MSK	Arthritis ***(mild)***, arthralgia or myalgia	Inflammatory pain over joints / muscle	0	1	2	3	4	
MSK	Other features	Myositis	No	YES → NEXT PAGE				
CardioResp	Pleurisy / Pericarditis	Convincing history and/or physical findings. Do not score if unsure.	0	1	2	3	4	
CardioResp	Pleural effusion with dyspnoea	Supportive imaging required	0	1	2	3	4	
CardioResp	Interstitial alveolitis/ pneumonitis	Supportive imaging required. Corrected Kco <70% normal or fall by > 20%.	0	1	2	3	4	
CardioResp	Other features	Any other cardiac or respiratory problem due to active SLE.	No	YES → NEXT PAGE				
NeuroPsych	Mononeuropathy (single or multiplex)	Supportive electrophysiology required	0	1	2	3	4	
NeuroPsych	Polyneuropathy	Acute symmetrical distal sensory and/or motor deficit (supportive electrophysiology required)	0	1	2	3	4	
NeuroPsych	Seizure disorder	Independent description of seizure by reliable witness	0	1	2	3	4	
NeuroPsych	Other features	Any other CNS or PNS feature due to active SLE	No	YES → NEXT PAGE				

Haematology			A	B	C	D	E	SCORE A - E
**Full blood count**<br>Circle patient's values only if due to ***active SLE***, i.e. exclude drug causes, iron deficiency	Hb	WITHOUT evidence of haemolysis		<8.0	8-10.9	>10.9	Never	
	Hb	WITH evidence of haemolysis	<8.0	8.0-9.9	>10		Never	
		Total WCC ($x10^6/L$)		<1.0	1-3.9	>3.9	Never	
		Neutrophils ($x10^6/L$)		<0.5	0.5-1.9	>1.9	Never	
		Lymphocytes ($x10^6/L$)			<1.0	>1.0	Never	
		Platelets ($x10^6/L$)	<25	25-49	50-149	>149	Never	
Other features		TTP or isolated Coombes	No	YES → NEXT PAGE				

**Gastrointestinal**<br>If active check next page		**Ophthalmic**<br>If active check next page	

For the following systems, circle all that apply and total the points to determine the domain score

Constitutional	
A	≥1200
B	≥ 200
C	≥ 10
D	0 previous
E	0 never

Constitutional		No	Imp	Same	Worse	New	SCORE A - E
Pyrexia	Documented Temp >37.5 (infection excluded)	0	10	1000	1000	1000	
Weight loss	Unintentional >5% loss in 1 month due to SLE	0	10	100	100	100	
Lymphadenopathy or splenomegaly	Lymph node > 1cm diameter	0	10	100	100	100	
Anorexia	Due to active SLE	0	10	100	100	100	

Renal	
A	≥1100
B	≥ 10
C	≥ 1
D	0 previous
E	0 never

Renal							SCORE A - E
**Select all that apply:**	Biopsy nephritis past 3 months?	1000	Active urinary sediment?	1000	Nephrotic syndrome?	100	
**Urinary PCR** (mg/mmol): or equivalent	25-50 ***or*** >25 and improved by ≥25%	1	50-100 and unimproved	10	>100 and unimproved	1000	
**GFR** (ml/min per $1.73m^2$):	<80 ml/min **&** <67% of previous	100	<50 ml/min **&** previously > 50	100			
**Serum creatinine:**	>130 µmol/L **&** >130% of previous	100	>130 µmol/L **&** >115% previous	10			
**Blood pressure** (mmHg):	Accelerated HTN (↑ to >170/110 within 1/12 with retinal changes)	100	BP >140/90 **&** ↑ by 30 systolic or 15 diastolic	1			

Update the final domain scores with any items recorded on page two | **No other items? BILAG COMPLETE**

Easy-BILAG v1.0 © University of Leeds

[26] From Isenberg, D.A., et al., BILAG 2004. Development and initial validation of an updated version of the British Isles Lupus Assessment Group's disease activity index for patients with systemic lupus erythematosus. Rheumatology (Oxford), 2005. 44(7): p. 902-6.

	ITEM	ABBREVIATED GLOSSARY	Nil	Imp	Same	Worse	New
Gastrointestinal	Lupus peritonitis	Serositis presenting as acute abdomen with rebound/guarding.	0	1	2	3	4
	Abdominal serositis or ascites	Not presenting as acute abdomen.	0	1	2	3	4
	Lupus enteritis / colitis	Vasculitis or inflammation of small or large bowel, with supportive imaging &/or biopsy.	0	1	2	3	4
	Malabsorption	Diarrhoea + abnormal D-xylose absorption / ↑faecal fat losses. Exclude Coeliac & gut vasculitis.	0	1	2	3	4
	Protein-losing enteropathy	See detailed glossary.	0	1	2	3	4
	Intestinal pseudo-obstruction	Subacute intestinal obstruction due to intestinal hypomotility.	0	1	2	3	4
	Lupus hepatitis	Raised transaminases, without AIH specific autoantibodies. Exclude drug- & viral hepatitis.	0	1	2	3	4
	Acute lupus cholecystitis	Exclude gallstones or infection.	0	1	2	3	4
	Acute lupus pancreatitis	Usually associated with multisystem involvement.	0	1	2	3	4
Ophthalmic	Orbital inflam / myositis / proptosis	Orbital inflammation + myositis / extra-ocular muscle swelling / proptosis. Imaging required.	0	1	2	3	4
	Keratitis - severe	Sight-threatening. Includes corneal melt and peripheral ulcerative keratitis.	0	1	2	3	4
	Keratitis - mild	Not sight-threatening.	0	1	2	3	4
	Anterior uveitis		0	1	2	3	4
	Post. uveitis/retinal vasculitis - *severe*	Sight-threatening and/or retinal vasculitis not due to vaso-occlusive disease.	0	1	2	3	4
	Post. uveitis/retinal vasculitis - *mild*	Not sight-threatening. Not due to vaso-occlusive disease.	0	1	2	3	4
	Episcleritis		0	1	2	3	4
	Scleritis - severe	Necrotising anterior scleritis. Ant &/or post scleritis requiring systemic therapy.	0	1	2	3	4
	Scleritis - mild	Anterior/posterior scleritis not requiring systemic steroids.	0	1	2	3	4
	Retinal / choroidal vaso-occlusive disease	See detailed glossary table.	0	1	2	3	4
	Isolated cottonwool spots	Also known as cystoid bodies.	0	1	2	3	4
	Optic neuritis	Exclude anterior ischaemic optic neuropathy.	0	1	2	3	4
	Anterior ischaemic optic neuropathy	Visual loss with pale swollen optic disc due to occlusion of posterior ciliary arteries.	0	1	2	3	4

OTHER PAGE 1 FEATURES: Record here and update the score for that domain on page one:

Mucocutan	Angio-oedema - severe	Urticaria variant in subcut, submucosal &deep dermal tissues. Potentially life-threatening.	0	1	2	3	4
	Angio-oedema - mild	As above but not life-threatening.	0	1	2	3	4
	Panniculitis/ bullous lupus - severe	See detailed glossary table.	0	1	2	3	4
	Panniculitis/ bullous lupus - mild	Affects <9% BSA & does not fulfil any criteria for severe panniculitis.	0	1	2	3	4
	Major cutaneous vasculitis/thrombosis	Cutaneous vasculitis/thrombosis → extensive gangrene /ulceration / skin infarction.	0	1	2	3	4
	Splinter haemorrhages		0	1	2	3	4
MSK	Myositis - *severe*	Significantly ↑ muscle enzymes with significant muscle weakness.	0	1	2	3	4
	Myositis – *mild*	Significantly ↑muscle enzymes + myalgia but no significant muscle weakness.	0	1	2	3	4
Cardiorespiratory	Myocarditis - mild	↑ cardiac enzymes &/or ECG changes. No heart failure/arrhythmia/valve dysfunction.	0	1	2	3	4
	Myo /Endocarditis + cardiac failure	See detailed glossary table.	0	1	2	3	4
	Arrhythmias	Due to myocarditis / non-infective inflammation. ECG evidence required.	0	1	2	3	4
	New valvular dysfunction	Due to myocarditis / non-infective inflammation. Supportive imaging required.	0	1	2	3	4
	Cardiac tamponade	Supportive imaging required.	0	1	2	3	4
	Pulmonary haemorrhage/vasculitis	With haemoptysis &/or dyspnoea &/or pulmonary HTN. Imaging &/or histology required.	0	1	2	3	4
	Shrinking lung syndrome	Acute ↓ lung volumes (< 70% predicted) + normal corrected Kco. Diagphragmatic dysfunction.	0	1	2	3	4
	Aortitis	+/- dissection with supportive imaging, claudication, bruits or BP discrepancy >10 mmHg.	0	1	2	3	4
	Coronary vasculitis	Imaging evidence of non-atheromatous coronary narrowing/ obstruction /aneurysm.	0	1	2	3	4
Neuropsychiatric	Aseptic meningitis	See detailed glossary table.	0	1	2	3	4
	Cerebral vasculitis	With features of vasculitis in another system. Supportive imaging &/or biopsy required.	0	1	2	3	4
	Demyelinating syndrome	Discrete white matter lesion + neurological deficit. Ideally ≥1 prior recorded event. Exclude MS.	0	1	2	3	4
	Myelopathy	Acute onset, rapidly evolving paraparesis, quadriparesis and/or sensory level. Exclude SOL.	0	1	2	3	4
	Acute confusional state	See detailed glossary table.	0	1	2	3	4
	Psychosis	Delusions &/or hallucinations. Excludes primary psychotic disorder, drugs or during delirium.	0	1	2	3	4
	Acute inflammatory demyelinating polyradiculoneuropathy	See detailed glossary table.	0	1	2	3	4
	Cranial neuropathy	Exclude optic neuropathy which is classified under ophthalmic system.	0	1	2	3	4
	Plexopathy	Supportive electrophysiology study required.	0	1	2	3	4
	Cognitive dysfunction	Sufficient to impair ADLs. Includes attention, memory, language, visuospatial, psychomotor.	0	1	2	3	4
	Status epilepticus	A seizure or seizures lasting >30 minutes without full recovery to baseline.	0	1	2	3	4
	Cerebrovascular disease	Not vasculitis. See detailed glossary table.	0	1	2	3	4
	Movement disorder	Exclude drug-induced.	0	1	2	3	4
	Autonomic disorder	See detailed glossary table.	0	1	2	3	4
	Cerebellar ataxia	Cerebellar ataxia in isolation of other CNS features. Usually subacute presentation.	0	1	2	3	4
	Severe lupus headache	Unremitting, Disabling, unresponsive to narcotics, >3 days. Exclude SOL and CNS infection.	0	1	2	3	4
	Headache from intracranial hypertension	Exclude cerebral sinus thrombosis.	0	1	2	3	4
Haem	TTP	Micro-angiopathic haemolytic anaemia + thrombocytopenia. Other causes excluded.	0	1	2	3	4
	Coombes positive (isolated)	Without evidence of haemolysis.	Negative		Positive		

© University of Leeds

14.11 Systemic Lupus Erythematosus Disease Activity Index 2000 (SLEDAI-2K)[27]

Weight	Score	Descriptor	Definition
8	▢	Anfall	Beginn vor kurzem; metabolische, infektiöse oder medikamentöse Ursachen müssen ausgeschlossen werden.
8	▢	Psychose	Fähigkeit, normale Aktivitäten auszuführen, aufgrund einer schweren Störung der Realitätswahrnehmung verändert; umfasst Halluzinationen, Inkohärenz, ausgeprägt lockeres Assoziieren, verarmten Gedankeninhalt, ausgeprägt unlogisches Denken, bizarres, desorganisiertes oder katatones Verhalten, Urämie und medikamentöse Ursachen müssen ausgeschlossen werden.
8	▢	Psycho-organisches Syndrom	Veränderte geistige Funktion mit Beeinträchtigung von Orientierung, Gedächtnis und anderen intellektuellen Funktionen; mit sehr schnellem Beginn und fluktuierenden klinischen Merkmalen; umfasst Beeinträchtigung des Bewusstseins mit verringerter Fähigkeit zu fokussieren und Unfähigkeit zu anhaltender Konzentration auf die Umgebung, plus mindestens zwei der folgenden Merkmale: Wahrnehmungsstörungen, inkohärentes Reden, Schlaflosigkeit oder Schläfrigkeit tagsüber oder gesteigerte oder gesenkte psychomotorische Aktivität; metabolische, infektiöse oder medikamentöse Ursachen müssen ausgeschlossen werden.
8	▢	Sehstörung	Retinale Veränderung bei SLE; umfassen Schwellungen der Nervenfasern (cytoid bodies), Netzhautblutungen, seröse Exsudate oder Hämorrhagien in der Choroidea oder Opticus-Neuritis; metabolische, infektiöse oder medikamentöse Ursachen müssen ausgeschlossen werden.
8	▢	Hirnnerven-Störung	Neu beginnende sensorische oder motorische Neuropathie, bei der die Hirnnerven betroffen sind.
8	▢	Lupus-Kopfschmerz	Schwerer, anhaltender Kopfschmerz; kann migräneartig sein, darf aber nicht auf Analgetika ansprechen.
8	▢	Zerebro-vaskulärer Insult	Neu beginnender zerebrovaskuläre(r) Insult(e); Ausschluss einer Arteriosklerose.
8	▢	Vaskulitis	Ulzeration; Gangrän; schmerzhafte Fingerknötchen; periungualer Infarkt; Splitterblutungen oder Nachweis einer Vaskulitis durch Biopsie oder Angiogramm.
4	▢	Arthritis	mehr als zwei Gelenke mit Schmerz und Entzündungszeichen (also Empfindlichkeit, Schwellung oder Erguss).
4	▢	Myositis	Schwäche oder Schmerzen proximaler Muskeln, einhergehend mit erhöhter Kreatin-Phosphokinase oder Aldolase oder Nachweis einer Myositis mittels Veränderungen im EMG oder Biopsie.
4	▢	Harnzylinder	granuläre Zylinder oder Erythrozyten-Zylinder.
4	▢	Hämaturie	>5 Erythroyzten im Mikroskopierfeld bei 400facher Vergrößerung (high power field hpf), Steine, Infektion oder andere Ursachen müssen ausgeschlossen werden.
4	▢	Proteinurie	> 0,5 g/24 Stunden; neu beginnend oder Steigerung in jüngster Zeit um mehr als 0,5 g/24 Stunden.
4	▢	Pyurie	> 5 Leukozyten im Mikroskopierfeld bei 400facher Vergrößerung; Ausschluss einer Infektion.
2	▢	Erythem	Neubeginn oder erneutes Auftreten eines Erythems.
2	▢	Alopezie	Neubeginn oder erneutes Auftreten von pathologischem Haarausfall, diffus oder als Alopezia areata.
2	▢	Schleimhaut-Ulzera	Neubeginn oder erneutes Auftreten oraler oder nasaler Ulzerationen.
2	▢	Pleuritis	Pleuritischer Schmerz im Brustkorb, mit Pleurareiben oder Erguss oder Verdickung der Pleura.
2	▢	Perikarditis	Perikardialer Schmerz mit mindestens einem der folgenden Merkmale: Reiben, Erguss oder Bestätigung durch EKG oder Echokardiogramm.
2	▢	Komplement-Verminderung	Verringerte Werte für CH50, C3 oder C4, unterhalb der für die Labortests normalen unteren Referenzbereiche.
2	▢	Erhöhte dsDNA-AK	im Labortest oberhalb des Normbereichs.
1	▢	Fieber	> 38 °C; Ausschluss einer infektiösen Ursache
1	▢	Thrombo-zytopenie	< 100.000 Thrombozyten pro µl
1	▢	Leukopenie	< 3.000 Leukozyten pro µl; Ausschluss einer medikamentösen Ursache

[27] From Gladman, D.D., D. Ibañez, and M.B. Urowitz, Systemic lupus erythematosus disease activity index 2000. J Rheumatol, 2002. 29(2): p. 288-91.

14.12 DORIS remission criteria of SLE[28]

- Clinical SLEDAI=0.

- Physician Global Assessment <0.5 (0–3).

    - Irrespective of serology.
    - The subject may be on antimalarials, low-dose glucocorticoids (prednisolone <5 mg/day), and/or stable immunosuppressives including biologics.

[28] From van Vollenhoven, R.F., et al., 2021 DORIS definition of remission in SLE: final recommendations from an international task force. Lupus Sci Med, 2021. 8(1).

14.13 modified Rodnan Skin Score (mRSS)[29]

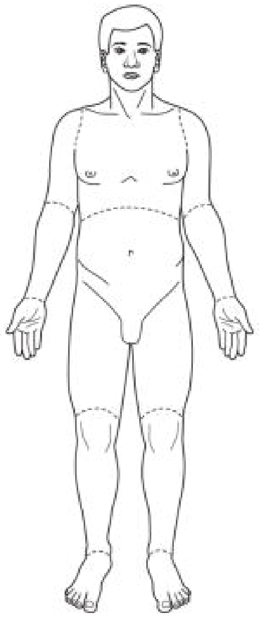

	Right				Left			
Fingers	0☐	1☐	2☐	3☐	0☐	1☐	2☐	3☐
Hands	0☐	1☐	2☐	3☐	0☐	1☐	2☐	3☐
Forearms	0☐	1☐	2☐	3☐	0☐	1☐	2☐	3☐
Upper Arms	0☐	1☐	2☐	3☐	0☐	1☐	2☐	3☐
Face			0☐	1☐	2☐	3☐		
Anterior Chest			0☐	1☐	2☐	3☐		
Abdomen			0☐	1☐	2☐	3☐		
Thighs	0☐	1☐	2☐	3☐	0☐	1☐	2☐	3☐
Legs	0☐	1☐	2☐	3☐	0☐	1☐	2☐	3☐
Feet	0☐	1☐	2☐	3☐	0☐	1☐	2☐	3☐
Column Totals								
Total:								
Key:	*0 – No Thickening*		*1 – Mild Thickening*		*2 – Moderate Thickening*		*3 – Severe Thickening*	
Notes:								

14.14 Physician's Global Assessment (PhGA) of extramuscular activity

Arzturteil zur extramuskulären Krankheitsaktivität

Keine Aktivität ______________________________ Stärkst mögliche Aktivität ___ ___ ___ (0-100 mm)

[29] From Khanna, D., et al., Standardization of the modified Rodnan skin score for use in clinical trials of systemic sclerosis. J Scleroderma Relat Disord, 2017. 2(1): p. 11-18.

14.15 Manual Muscle Testing (MMT)[30]

MANUAL MUSCLE TESTING PROCEDURES

Key to Muscle Grading

	Function of the Muscle	Grade		
**No Movement**	No contractions felt in the muscle	0	0	Zero
	Tendon becomes prominent or feeble contraction felt in the muscle, but no visible movement of the part	T	1	Trace
**Test Movement**	**MOVEMENT IN HORIZONTAL PLANE**			
	Moves through partial range of motion	1	2-	Poor-
	Moves through complete range of motion	2	2	Poor
	**ANTIGRAVITY POSITION**<br>Moves through partial range of motion	3	2+	
**Test Position**	*Gradual* release from test position	4	3-	Fair-
	Holds test position (no added pressure)	5	3	Fair
	Holds test position against slight pressure	6	3+	Fair+
	Holds test position against slight to moderate pressure	7	4-	Good-
	Holds test position against moderate pressure	8	4	Good
	Holds test position against moderate to strong pressure	9	4+	Good+
	Holds test position against strong pressure	10	5	Normal

TESTING POSITIONS

Muscle Groups	Anti-Gravity	Gravity Eliminated
Trapezius (shoulder elevators)	Sitting	Supine
Deltoid middle (shoulder abductors)	Sitting	Supine
Biceps brachii (elbow flexors)	Sitting	Sidelying
Wrist extensors	Sitting (pronation)	Sitting (neutral)
Wrist flexors	Sitting (supination)	Sitting (neutral)
Iliopsoas (hip flexors)	Sitting	Sidelying
Quadriceps femoris (knee extensors)	Sitting	Sidelying
Ankle dorsiflexors	Sitting	Sidelying
Neck flexors	Supine	Sidelying
Gluteus medius (hip abductors)	Sidelying	Supine
Neck extensors	Prone	Sidelying
Gluteus maximus (hip extensors)	Prone	Sidelying
Hamstrings (knee flexors)	Prone	Sidelying
Ankle plantarflexors	Prone/Standing	Sidelying

[30] From Kendall, [illegible]. 4 ed. 1993: Lippincott, Willian[illegible]

MMT Considerations to Promote Reliability

MMT Grades (0 – 10 scale)	Comments
0 – T	Palpation skill may confound the distinction between the "0" and "T" score; use trained and experienced clinicians.
1 – 2	Adjust range of motion criterion to accommodate for muscle contractures.
3	This grade can only be assigned to muscles tested in the standard (against gravity) testing position.
4	Gradual descent from testing position to resting position should last at least 3 seconds
5	Test position should be held for 3 seconds
3 – 5	MMT grades in this range should be re-tested for the next highest grade after 60 seconds of recovery time.
6 – 10	MMT grades in this range are heavily influenced by the stature of the subject and tester. Attempt to use back-up testers of a similar stature to the primary tester.<br>All MMT in this range should involve a force application time of 3 seconds.

14.16 2016 ACR/EULAR Criteria for Clinical Response in Adult DM/PM[31]

Web calculator for 2016 ACR/EULAR Criteria for Minimal, Moderate, and Major Clinical Response in Adult Dermatomyositis and Polymyositis

The Myositis Response Criteria was validated using all 6 core set measures.

At a minimum, you must enter:
1. Physician Global Disease Activity AND,
3. Manual Muscle Testing AND
at least 2 other core set measures in order for a Total Improvement Score to be calculated.

Core Set Measure			BASELINE	FOLLOW-UP	Change	Relative % Change	Absolute % Change	Improvement Score
1. Physician Global Disease Activity [0.0-10.0 cm]								
2. Patient Global Disease Activity [0.0-10.0 cm]								
3. Manual Muscle Testing (MMT)	Maximum MMT Score Possible:							
4. Health Assessment Questionnaire (HAQ) [0.000-3.000]								
5. Extra-muscular Disease Activity [0.0-10.0 cm]								
6. Muscle Enzymes<br>Please select the UNITS first, and then enter each serum muscle enzyme level tested and the associated upper limit of normal.		Upper Limit of Normal	BASELINE	FOLLOW-UP				
○ [Unit/L]<br>○ [microkat/L]	Creatine Kinase (CK)							
	Lactate Dehydrogenase (LDH)							
	Aspartate Aminotransferase (AST)							
	Alanine Aminotransferase (ALT)							
	Aldolase							
Calculate Most Abnormal Muscle Enzyme								

	No Improvement Threshold	< 20	Total Improvement Score:
	Minimal Improvement Threshold	20	
	Moderate Improvement Threshold	40	
	Major Improvement Threshold	60	

Clear Form | Calculate Total Improvement Score

[31] Online Calculator: https://www.niehs.nih.gov/research/resources/imacs/response_criteria/adult.html
Users Guide:
https://www.niehs.nih.gov/research/resources/imacs/response_criteria/users_guide_for_the_adult_dermatomyositis_and_polymyositis_response_criteria_online_calculator_508.pdf

14.17 2019 ACR/EULAR classification criteria of SLE[32]

Entry criterion
Antinuclear antibodies (ANA) at a titer of ≥1:80 on HEp-2 cells or an equivalent positive test (ever)

↓

If absent, do not classify as SLE
If present, apply additive criteria

↓

Additive criteria
Do not count a criterion if there is a more likely explanation than SLE.
Occurrence of a criterion on at least one occasion is sufficient.
SLE classification requires at least one clinical criterion and ≥10 points.
Criteria need not occur simultaneously.
Within each domain, only the highest weighted criterion is counted toward the total score§.

Clinical domains and criteria	Weight
Constitutional	
Fever	2
Hematologic	
Leukopenia	3
Thrombocytopenia	4
Autoimmune hemolysis	4
Neuropsychiatric	
Delirium	2
Psychosis	3
Seizure	5
Mucocutaneous	
Non-scarring alopecia	2
Oral ulcers	2
Subacute cutaneous OR discoid lupus	4
Acute cutaneous lupus	6
Serosal	
Pleural or pericardial effusion	5
Acute pericarditis	6
Musculoskeletal	
Joint involvement	6
Renal	
Proteinuria >0.5g/24h	4
Renal biopsy Class II or V lupus nephritis	8
Renal biopsy Class III or IV lupus nephritis	10

Immunology domains and criteria	Weight
Antiphospholipid antibodies	
Anti-cardiolipin antibodies OR	
Anti-β2GP1 antibodies OR	
Lupus anticoagulant	2
Complement proteins	
Low C3 OR low C4	3
Low C3 AND low C4	4
SLE-specific antibodies	
Anti-dsDNA antibody* OR	
Anti-Smith antibody	6

Total score:

↓

Classify as Systemic Lupus Erythematosus with a score of 10 or more if entry criterion fulfilled.

[32] From Aringer, M., et al., 2019 European League Against Rheumatism/American College of Rheumatology classification criteria for systemic lupus erythematosus. Ann Rheum Dis, 2019. 78(9): p. 1151-1159.

14.18 2013 ACR/EULAR classification criteria of SSc[33]

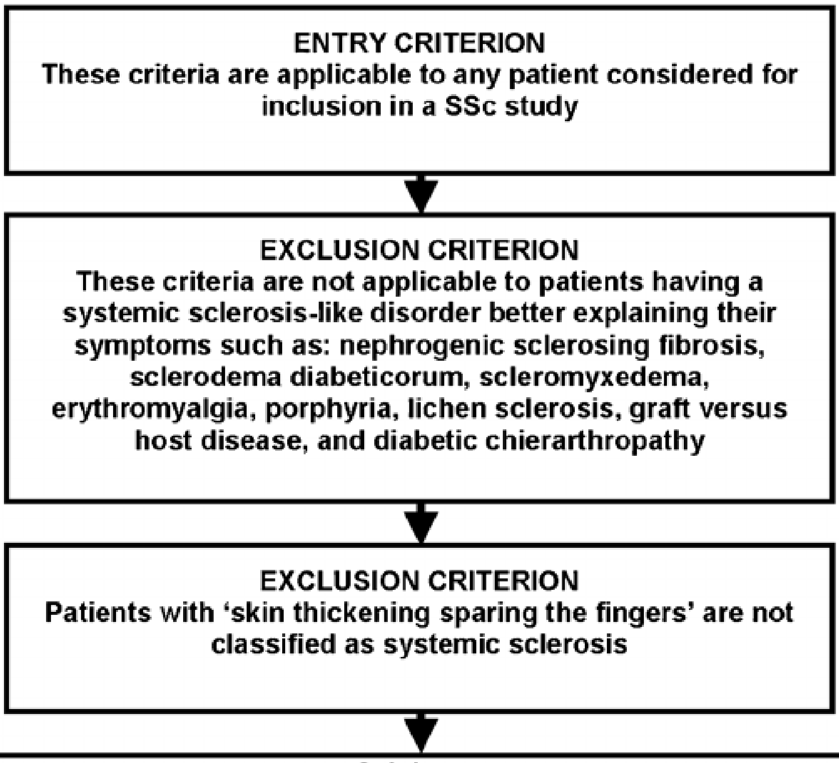

Items	Sub-items	Weight
SUFFICIENT CRITERION		9
Skin thickening of the fingers of both hands extending proximal to the metacarpophalangeal joints		
Skin thickening of the fingers<br>*(only count the highest score)*	Puffy fingers<br>Whole Finger, distal to MCP	2<br>4
Finger tip lesions	Digital Tip Ulcers	2
(only count the highest score)	Pitting Scars	3
Telangiectasia		2
Abnormal nailfold capillaries		2
Pulmonary arterial hypertension and/or Interstitial lung Disease		2
Raynaud's phenomenon		3
Scleroderma related antibodies	(any of anti-centromere, anti-topoisomerasel [anti-ScL 70], anti-RNA polymerase III)	3
TOTAL SCORE:		

Patients having a total score of 9 or more are classified as having definite systemic sclerosis.

[33] From [illegible] College of Rheumatology/European League against Rheumatism collaborative initiative. Arthritis Rheum, 2013. 65(11): p. 2737-47.

14.19 2017 ACR/EULAR classification criteria for probable or definite DM or PM[34]

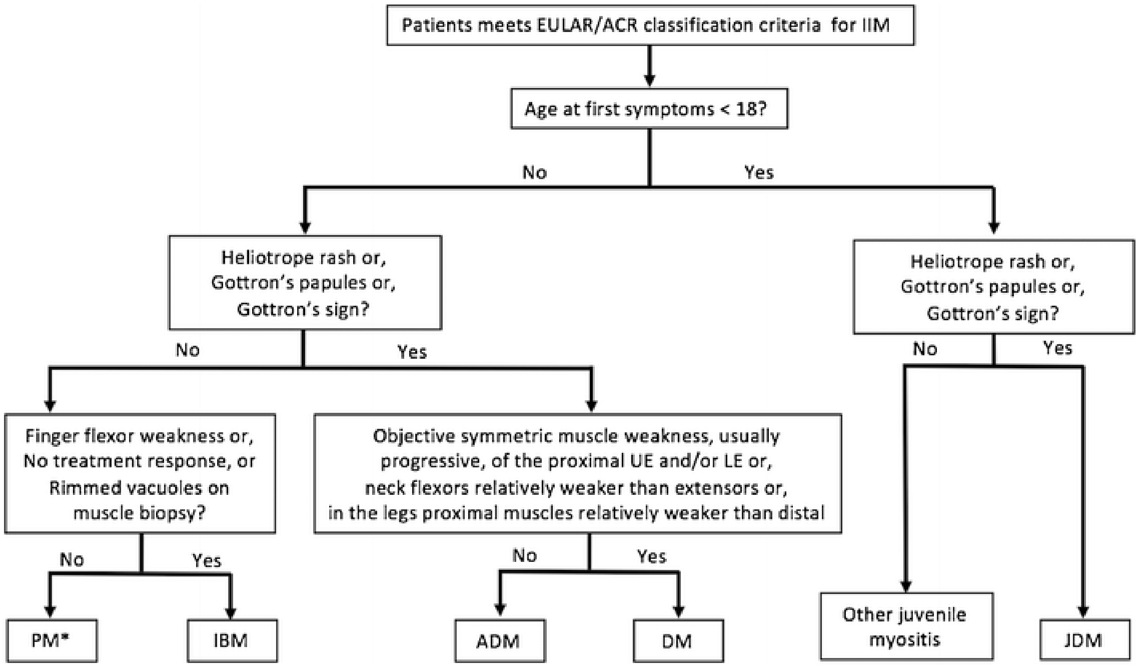

*The PM subset includes immune-mediated necrotizing myopathies (IMNM). PM, polymyositis; IBM, inclusion body myositis; ADM, amyopathic dermatomyositis; DM, dermatomyositis; JDM, juvenile dermatomyositis

[34] From Lundberg, I.E., et al., 2017 European League Against Rheumatism/American College of Rheumatology classification criteria for adult and juvenile idiopathic inflammatory myopathies and their major subgroups. Ann Rheum Dis, 2017. 76(12): p. 1955-1964.

15 REFERENCES

1. Dull, T., et al., *A third-generation lentivirus vector with a conditional packaging system.* J Virol, 1998. **72**(11): p. 8463-71.
2. Naldini, L. and I.M. Verma, *Lentiviral vectors.* Adv Virus Res, 2000. **55**: p. 599-609.
3. Zufferey, R., et al., *Self-inactivating lentivirus vector for safe and efficient in vivo gene delivery.* J Virol, 1998. **72**(12): p. 9873-80.
4. Kaiser, A.D., et al., *Towards a commercial process for the manufacture of genetically modified T cells for therapy.* Cancer Gene Ther, 2015. **22**(2): p. 72-8.
5. Merrill, J.T., et al., *Efficacy and safety of rituximab in moderately-to-severely active systemic lupus erythematosus: the randomized, double-blind, phase II/III systemic lupus erythematosus evaluation of rituximab trial.* Arthritis Rheum, 2010. **62**(1): p. 222-33.
6. Smith, V., et al., *Rituximab in diffuse cutaneous systemic sclerosis: an open-label clinical and histopathological study.* Ann Rheum Dis, 2010. **69**(1): p. 193-7.
7. Oddis, C.V., et al., *Rituximab in the treatment of refractory adult and juvenile dermatomyositis and adult polymyositis: a randomized, placebo-phase trial.* Arthritis Rheum, 2013. **65**(2): p. 314-24.
8. Hoy, S.M., *Tafasitamab: First Approval.* Drugs, 2020. **80**(16): p. 1731-1737.
9. Kamburova, E.G., et al., *A single dose of rituximab does not deplete B cells in secondary lymphoid organs but alters phenotype and function.* Am J Transplant, 2013. **13**(6): p. 1503-11.
10. Thurlings, R.M., et al., *Synovial tissue response to rituximab: mechanism of action and identification of biomarkers of response.* Ann Rheum Dis, 2008. **67**(7): p. 917-25.
11. Tabbara, N., et al., *Anti-CD19 CAR T-cell therapy remission despite prior anti-CD19 antibody Tafasitamab in relapsed/refractory DLBCL.* Leuk Res Rep, 2021. **16**: p. 100260.
12. Kaul, A., et al., *Systemic lupus erythematosus.* Nat Rev Dis Primers, 2016. **2**: p. 16039.
13. Cervera, R., et al., *Morbidity and mortality in systemic lupus erythematosus during a 10-year period: a comparison of early and late manifestations in a cohort of 1,000 patients.* Medicine (Baltimore), 2003. **82**(5): p. 299-308.
14. van den Hoogen, F., et al., *2013 classification criteria for systemic sclerosis: an American College of Rheumatology/European League against Rheumatism collaborative initiative.* Arthritis Rheum, 2013. **65**(11): p. 2737-47.
15. Melissaropoulos, K. and D. Daoussis, *B cells in systemic sclerosis: from pathophysiology to treatment.* Clin Rheumatol, 2021. **40**(7): p. 2621-2631.
16. Bosello, S., et al., *Characterization of inflammatory cell infiltrate of scleroderma skin: B cells and skin score progression.* Arthritis Res Ther, 2018. **20**(1): p. 75.
17. Ebata S., Yoshizaki A., and KojiOba K., *Safety and efficacy of rituximab in systemic sclerosis (DESIRES): a double-blind, investigator-initiated, randomised, placebo-controlled trial.* Lancet Rheumatol, 2021. **3**: p. e489-e497.
18. Lundberg, I.E., M. de Visser, and V.P. Werth, *Classification of myositis.* Nat Rev Rheumatol, 2018. **14**(5): p. 269-278.
19. Kansal, R., et al., *Sustained B cell depletion by CD19-targeted CAR T cells is a highly effective treatment for murine lupus.* Sci Transl Med, 2019. **11**(482).
20. Jin, X., et al., *Therapeutic efficacy of anti-CD19 CAR-T cells in a mouse model of systemic lupus erythematosus.* Cell Mol Immunol, 2021. **18**(8): p. 1896-1903.
21. Afzali, A.M., et al., *Animal models in idiopathic inflammatory myopathies: How to overcome a translational roadblock?* Autoimmun Rev, 2017. **16**(5): p. 478-494.

22. Chakrabarti, S., et al., *Impaired membrane resealing and autoimmune myositis in synaptotagmin VII-deficient mice.* J Cell Biol, 2003. **162**(4): p. 543-9.
23. Soare, A., *Updates on animal models of systemic sclerosis.* Scleroderma and Related Disorders, 2016. **1**: p. 266-276.
24. Artlett, C.M., *Animal models of systemic sclerosis: their utility and limitations.* Open Access Rheumatol, 2014. **6**: p. 65-81.
25. Mougiakakos, D., et al., *CD19-Targeted CAR T Cells in Refractory Systemic Lupus Erythematosus.* N Engl J Med, 2021. **385**(6): p. 567-569.
26. Mackensen, A., et al., *Anti-CD19 CAR T cell therapy for refractory systemic lupus erythematosus.* Nat Med, 2022. **28**(10): p. 2124-2132.
27. Mueller, F., *CD19-Targeted CAR T Cells in Refractory Anti-Synthetase Syndrome.* Lancet, 2023.
28. Bergmann, C., *Treatment of a patient with severe systemic sclerosis using CD19-targeted CAR-T-cells.* Ann Rheum Dis, 2023. **submitted**.
29. Wiendl, H., Schmidt, J. *Myositissyndrome – Leitlinien für Diagnostik und Therapie in der Neurologie*. 2022; Available from: https://www.awmf.org/uploads/tx_szleitlinien/030-054l_S2k_Myositissyndrome_2022-06_04.pdf.
30. *Diagnostik und Therapie der systemischen Sklerose*. Available from: https://www.awmf.org/leitlinien/detail/anmeldung/1/ll/060-014.html.
31. Kowal-Bielecka, O., et al., *Update of EULAR recommendations for the treatment of systemic sclerosis.* Ann Rheum Dis, 2017. **76**(8): p. 1327-1339.
32. *Systemischer Lupus erythematodes: Diagnose und Management*. Available from: https://www.awmf.org/leitlinien/detail/anmeldung/1/ll/060-008.html.
33. Fanouriakis, A., et al., *2019 update of the EULAR recommendations for the management of systemic lupus erythematosus.* Ann Rheum Dis, 2019. **78**(6): p. 736-745.
34. Fanouriakis, A., et al., *2019 Update of the Joint European League Against Rheumatism and European Renal Association-European Dialysis and Transplant Association (EULAR/ERA-EDTA) recommendations for the management of lupus nephritis.* Ann Rheum Dis, 2020. **79**(6): p. 713-723.
35. Tian, J., et al., *Risk of adverse events from different drugs for SLE: a systematic review and network meta-analysis.* Lupus Sci Med, 2018. **5**(1): p. e000253.
36. Shen, Y., et al., *Association of intrarenal B-cell infiltrates with clinical outcome in lupus nephritis: a study of 192 cases.* Clin Dev Immunol, 2012. **2012**: p. 967584.
37. Espeli, M., et al., *Local renal autoantibody production in lupus nephritis.* J Am Soc Nephrol, 2011. **22**(2): p. 296-305.
38. Kraaij, M.D. and J.M. van Laar, *The role of B cells in systemic sclerosis.* Biologics, 2008. **2**(3): p. 389-95.
39. Lafyatis, R., et al., *B cell infiltration in systemic sclerosis-associated interstitial lung disease.* Arthritis Rheum, 2007. **56**(9): p. 3167-8.
40. Preuße, C., et al., *Skeletal muscle provides the immunological micro-milieu for specific plasma cells in anti-synthetase syndrome-associated myositis.* Acta Neuropathol, 2022. **144**(2): p. 353-372.
41. Peng, M., et al., *Association between nonspecific interstitial pneumonia and presence of CD20+ B lymphocytes within pulmonary lymphoid follicles.* Sci Rep, 2017. **7**(1): p. 16912.
42. Anolik, J.H., et al., *Delayed memory B cell recovery in peripheral blood and lymphoid tissue in systemic lupus erythematosus after B cell depletion therapy.* Arthritis Rheum, 2007. **56**(9): p. 3044-56.

43. Vos, K., et al., *Early effects of rituximab on the synovial cell infiltrate in patients with rheumatoid arthritis.* Arthritis Rheum, 2007. **56**(3): p. 772-8.
44. Dudley, M.E., et al., *Cancer regression and autoimmunity in patients after clonal repopulation with antitumor lymphocytes.* Science, 2002. **298**(5594): p. 850-4.
45. Morgan, R.A., et al., *Cancer regression in patients after transfer of genetically engineered lymphocytes.* Science, 2006. **314**(5796): p. 126-9.
46. Davila, M.L., et al., *Efficacy and toxicity management of 19-28z CAR T cell therapy in B cell acute lymphoblastic leukemia.* Sci Transl Med, 2014. **6**(224): p. 224ra25.
47. Kochenderfer, J.N., et al., *Donor-derived CD19-targeted T cells cause regression of malignancy persisting after allogeneic hematopoietic stem cell transplantation.* Blood, 2013. **122**(25): p. 4129-39.
48. Lee, D.W., et al., *T cells expressing CD19 chimeric antigen receptors for acute lymphoblastic leukaemia in children and young adults: a phase 1 dose-escalation trial.* Lancet, 2015. **385**(9967): p. 517-528.
49. Maude, S.L., et al., *Chimeric antigen receptor T cells for sustained remissions in leukemia.* N Engl J Med, 2014. **371**(16): p. 1507-17.
50. Turtle, C.J., et al., *CD19 CAR-T cells of defined CD4+:CD8+ composition in adult B cell ALL patients.* J Clin Invest, 2016. **126**(6): p. 2123-38.
51. Turtle, C.J., et al., *Immunotherapy of non-Hodgkin's lymphoma with a defined ratio of CD8+ and CD4+ CD19-specific chimeric antigen receptor-modified T cells.* Sci Transl Med, 2016. **8**(355): p. 355ra116.
52. Larson, R.C. and M.V. Maus, *Recent advances and discoveries in the mechanisms and functions of CAR T cells.* Nat Rev Cancer, 2021. **21**(3): p. 145-161.
53. Schuster, S.J., et al., *Tisagenlecleucel in Adult Relapsed or Refractory Diffuse Large B-Cell Lymphoma.* N Engl J Med, 2019. **380**(1): p. 45-56.
54. Neelapu, S.S., *Managing the toxicities of CAR T-cell therapy.* Hematol Oncol, 2019. **37 Suppl 1**: p. 48-52.
55. van Vollenhoven, R.F., et al., *2021 DORIS definition of remission in SLE: final recommendations from an international task force.* Lupus Sci Med, 2021. **8**(1).
56. Roofeh, D., et al., *Tocilizumab Prevents Progression of Early Systemic Sclerosis-Associated Interstitial Lung Disease.* Arthritis Rheumatol, 2021. **73**(7): p. 1301-1310.
57. Aggarwal, R., et al., *2016 American College of Rheumatology/European League Against Rheumatism Criteria for Minimal, Moderate, and Major Clinical Response in Adult Dermatomyositis and Polymyositis: An International Myositis Assessment and Clinical Studies Group/Paediatric Rheumatology International Trials Organisation Collaborative Initiative.* Arthritis Rheumatol, 2017. **69**(5): p. 898-910.
58. Isenberg, D.A., et al., *BILAG 2004. Development and initial validation of an updated version of the British Isles Lupus Assessment Group's disease activity index for patients with systemic lupus erythematosus.* Rheumatology (Oxford), 2005. **44**(7): p. 902-6.
59. Gladman, D.D., D. Ibañez, and M.B. Urowitz, *Systemic lupus erythematosus disease activity index 2000.* J Rheumatol, 2002. **29**(2): p. 288-91.
60. Khanna, D., et al., *Standardization of the modified Rodnan skin score for use in clinical trials of systemic sclerosis.* J Scleroderma Relat Disord, 2017. **2**(1): p. 11-18.
61. Kendall, F.P., McCreary, E.K. and Provance, P.G., *Muscle Testing and Function*. 4 ed. 1993: Lippincott, Williams and Wilkins, Philadelphia.
62. Lee, D.W., et al., *Current concepts in the diagnosis and management of cytokine release syndrome.* Blood, 2014. **124**(2): p. 188-95.

63. Lee, D.W., et al., *ASTCT Consensus Grading for Cytokine Release Syndrome and Neurologic Toxicity Associated with Immune Effector Cells.* Biol Blood Marrow Transplant, 2019. **25**(4): p. 625-638.
64. Aringer, M., et al., *2019 European League Against Rheumatism/American College of Rheumatology classification criteria for systemic lupus erythematosus.* Ann Rheum Dis, 2019. **78**(9): p. 1151-1159.
65. Lundberg, I.E., et al., *2017 European League Against Rheumatism/American College of Rheumatology classification criteria for adult and juvenile idiopathic inflammatory myopathies and their major subgroups.* Ann Rheum Dis, 2017. **76**(12): p. 1955-1964.
66. Ng, C.L., D.D. Ho, and S.P. Chow, *The Moberg pickup test: results of testing with a standard protocol.* J Hand Ther, 1999. **12**(4): p. 309-12.
67. Chung, K.C., et al., *The Michigan Hand Outcomes Questionnaire (MHQ): assessment of responsiveness to clinical change.* Ann Plast Surg, 1999. **42**(6): p. 619-22.
68. Chung, K.C., et al., *Predictors of patient satisfaction in an outpatient plastic surgery clinic.* Ann Plast Surg, 1999. **42**(1): p. 56-60.
69. Bryant, J. and R. Day, *Incorporating toxicity considerations into the design of two-stage phase II clinical trials.* Biometrics, 1995. **51**(4): p. 1372-83.
