# Supplementary material for: CD19 CAR-T cells for treatment-refractory autoimmune diseases: the phase 1/2 CASTLE basket trial
Source: Nat Med. 2026 Jan 7;32(3):1142–51. doi: 10.1038/s41591-025-04185-6 (PMC13004673; doi:10.1038/s41591-025-04185-6)
Supplement: Supplementary file 2 — Reporting Summary [file 41591_2025_4185_MOESM2_ESM.pdf]

nature portfolio

Corresponding author(s): Georg Schett

Last updated by author(s): Dec 5, 2025

Reporting Summary

Nature Portfolio wishes to improve the reproducibility of the work that we publish. This form provides structure for consistency and transparency in reporting. For further information on Nature Portfolio policies, see our Editorial Policies and the Editorial Policy Checklist.

Please do not complete any field with "not applicable" or n/a. Refer to the help text for what text to use if an item is not relevant to your study.
For final submission: please carefully check your responses for accuracy; you will not be able to make changes later.

Statistics

For all statistical analyses, confirm that the following items are present in the figure legend, table legend, main text, or Methods section.

n/a	Confirmed	
☐	☒	The exact sample size (*n*) for each experimental group/condition, given as a discrete number and unit of measurement
☐	☒	A statement on whether measurements were taken from distinct samples or whether the same sample was measured repeatedly
☐	☒	The statistical test(s) used AND whether they are one- or two-sided<br>*Only common tests should be described solely by name; describe more complex techniques in the Methods section.*
☒	☐	A description of all covariates tested
☒	☐	A description of any assumptions or corrections, such as tests of normality and adjustment for multiple comparisons
☐	☒	A full description of the statistical parameters including central tendency (e.g. means) or other basic estimates (e.g. regression coefficient) AND variation (e.g. standard deviation) or associated estimates of uncertainty (e.g. confidence intervals)
☐	☒	For null hypothesis testing, the test statistic (e.g. *F*, *t*, *r*) with confidence intervals, effect sizes, degrees of freedom and *P* value noted<br>*Give P values as exact values whenever suitable.*
☒	☐	For Bayesian analysis, information on the choice of priors and Markov chain Monte Carlo settings
☒	☐	For hierarchical and complex designs, identification of the appropriate level for tests and full reporting of outcomes
☒	☐	Estimates of effect sizes (e.g. Cohen's *d*, Pearson's *r*), indicating how they were calculated

Our web collection on statistics for biologists contains articles on many of the points above.

Software and code

Policy information about availability of computer code

Data collection	paper-based CRF
Data analysis	FlowJo v10, Graph Pad Prism v10, R v4.0.1

For manuscripts utilizing custom algorithms or software that are central to the research but not yet described in published literature, software must be made available to editors and reviewers. We strongly encourage code deposition in a community repository (e.g. GitHub). See the Nature Portfolio guidelines for submitting code & software for further information.

Data

Policy information about availability of data

All manuscripts must include a data availability statement. This statement should provide the following information, where applicable:

- Accession codes, unique identifiers, or web links for publicly available datasets
- A description of any restrictions on data availability
- For clinical datasets or third party data, please ensure that the statement adheres to our policy

All numeric data of this manuscript can be obtained from the corresponding author upon request via e-mail to georg.schett@uk-erlangen.de. Patient data can only be shared in pseudonymized form. Otherwise, there are no restrictions to data access.

Research involving human participants, their data, or biological material

Policy information about studies with human participants or human data. See also policy information about sex, gender (identity/presentation), and sexual orientation and race, ethnicity and racism.

Reporting on sex and gender	Sex of the trial participants is reported in this phase I/II trial. No sex or gender analysis was carried out due to the small sample size and the different diseases.
Reporting on race, ethnicity, or other socially relevant groupings	These data are not reported.
Population characteristics	All patients fulfilled the ACR/EULAR criteria for the respective diseases.
Recruitment	Eligible patients were recruited at the discretion of the investigators based on inclusion and exclusion criteria. There was no compensation of the participants.
Ethics oversight	The study protocol and all amendments were approved by an independent ethics committee. All recruited patients provided a written informed consent in compliance with the Declaration of Helsinki.

Note that full information on the approval of the study protocol must also be provided in the manuscript.

Field-specific reporting

Please select the one below that is the best fit for your research. If you are not sure, read the appropriate sections before making your selection.

☒ Life sciences ☐ Behavioural & social sciences ☐ Ecological, evolutionary & environmental sciences

For a reference copy of the document with all sections, see nature.com/documents/nr-reporting-summary-flat.pdf

Life sciences study design

All studies must disclose on these points even when the disclosure is negative.

Sample size	A Bryant and Day two-stage optimal design was used. In the first stage 8 patients were recruited. After meeting the primary safety endpoint with respect to dose limiting toxicity, 16 additional patients were recruited in the second stage.
Data exclusions	There were 4 screen failures (2 for not meeting lung function inclusion criteria, 2 for concomitant infections)
Replication	The results from the phase 2 illustrated reproducibility of the results from the phase 1.
Randomization	There was no randomization as Phase 1 cell therapy studies are usually performed as single-arm studies.
Blinding	There was no blinding, as this was an open-label trial.

Reporting for specific materials, systems and methods

We require information from authors about some types of materials, experimental systems and methods used in many studies. Here, indicate whether each material, system or method listed is relevant to your study. If you are not sure if a list item applies to your research, read the appropriate section before selecting a response.

Materials & experimental systems

n/a	Involved in the study	
☐	☒	Antibodies
☒	☐	Eukaryotic cell lines
☒	☐	Palaeontology and archaeology
☒	☐	Animals and other organisms
☐	☒	Clinical data
☒	☐	Dual use research of concern
☒	☐	Plants

Methods

n/a	Involved in the study	
☒	☐	ChIP-seq
☐	☒	Flow cytometry
☒	☐	MRI-based neuroimaging

Antibodies

Antibodies used	The following commercial ELISAs were used:

Antibodies used

VaccZyme Tetanus toxoid IgG (Binding Site, Catalog #: MK010)
Anti-dsDNA antibodies (Orgentec Catalog #: ORG 604_5)
Anti-Nucleosomes (Orgentec, Catalog #: ORG 528_4)
Anti-SM (Orgentec, Catalog #: ORG 511_4)
Anti SSA/Ro52 (Orgentec, Catalog #: ORG 652_4)
Epstein-Barr Virus EBNA1 IgG (Virion/Serion, Catalog #: IC1023)
Epstein-Barr Virus VCA IgG (Virion/Serion, Catalog #: IC1022)
Varicella Zoster Virus IgG (Virion/Serion, Catalog #: IC1046)
Mumps Virus IgG (Virion/Serion, Catalog #: IC1037)
Measles Virus IgG (Virion/Serion, Catalog #: IC1036)
Rubella Virus IgG (Abbott ARCHITECT)
SARS-CoV2 IgG (EUROIMMUN, Catalog #: EI 2606-9601 G)
Inflammatory Myopathy EUROLINE (EUROIMMUN, Catalog #: DL 1530-1601-4 G)
Systemic sclerosis EUROLINE (EUROIMMUN, Catalog #: DL 1532-1601 G)

The following antibodies were used for B cell compartment:
CD19: clone BV421 (HIB19), Biolegend, dilution: 1/400, Catalog #: 302233
CD20: clone AF700 (2H7), Biolegend, dilution: 1/1000, Catalog #: 302322
CD27: clone PE-Cy7 (M-T271), Biolegend, dilution: 1/100, Catalog #: 356412
CD38: clone: PerCP-Cy5.5 (HIT2), BD Biosciences, dilution: 1/200, Catalog #: 551400
CD21: clone PE (Bu32), Biolegend, dilution: 1/200, Catalog #: 354904
IgD: clone BV785 (IA6-2), Biolegend,dilution: 1/400, Catalog #: 348242
IgD: clone PE (IA6-2), Biolegend, dilution: 1/400, catalog # 348204
CD3 (dump): clone SparkBlue 550 (SK7), Biolegend, dilution: 1/400, Catalog #: 344852
Viability: Fixable Viability Stain 780, ThermoFisher, dilution: 1:2000. Catalog # 65-0865-14
Zombie NIR: Fixable Viability Kit, Biolegend, dilution: 1:1000, Catalog #: 423106

The following anti-human antibodies were used for flow cytometry for monitoring leukocytes and CAR T cells after treatment: anti-CD3 (clone SK7), anti-CD4 (clone SK3), anti-CD8 (clone SK1), anti-CD14 (clone MφP9), anti-CD19 (clone SJ25C1), anti-CD45 (clone 2D1), anti-CD56 (clone NCAM16.2; all BD Biosciences, Heidelberg, Germany) and CD19 CAR Detection Reagent (clone REA746; Miltenyi Biotec, Bergisch-Gladbach, Germany).

Validation

Validation is provided by the commercial manufacturers:
Binding Site:
https://www.thermofisher.com/bindingsite/wo/en/products/immunoassays/vaccine-response.html
Orgentec:
https://www.orgentec.com/de/produkte/elisa/autoimmundiagnostik/rheumadiagnostik/Anti-dsDNA.html
https://www.orgentec.com/de/produkte/elisa/autoimmundiagnostik/rheumadiagnostik/ORG+528.html
https://www.orgentec.com/de/produkte/elisa/autoimmundiagnostik/rheumadiagnostik/ORG+511.html
https://www.orgentec.com/de/produkte/elisa/autoimmundiagnostik/rheumadiagnostik/ORG+652.html
Virion/Serion:
https://www.serion-diagnostics.de/produkte/serion-kontrollen/serion-immunocontrol/
Abbott:
DOI: 10.1016/j.jcv.2010.07.011
Euroimmun:
https://www.euroimmun.de/produkte/infektion/pd/atemwegsinfektionen/2606/2/172507/
https://www.euroimmun.de/produkte/produktdetails/1530-4/3/170513/
https://www.euroimmun.com/products/autoimmunity/pd/rheumatology/connective-tissue-diseases/1532/3/170550/
Biolegend:
https://www.biolegend.com/en-us/products/brilliant-violet-421-anti-human-cd19-antibody-7144
https://www.biolegend.com/en-us/products/alexa-fluor-700-anti-human-cd20-antibody-3400
https://www.biolegend.com/en-us/products/pe-cyanine7-anti-human-cd27-antibody-8640
https://www.biolegend.com/en-us/products/pe-anti-human-cd21-antibody-8033
https://www.biolegend.com/en-us/products/brilliant-violet-785-anti-human-igd-antibody-13612
https://www.biolegend.com/en-us/products/pe-anti-human-igd-antibody-6532
https://www.biolegend.com/en-us/products/spark-blue-550-anti-human-cd3-antibody-18495
https://www.biolegend.com/en-gb/products/zombie-nir-fixable-viability-kit-8657?GroupID=BLG2181
ThermoFisher:
https://www.thermofisher.com/order/catalog/product/65-0865-14/
BD BioScience:
https://www.bdbiosciences.com/en-eu/products/reagents/flow-cytometry-reagents/research-reagents/quality-and-reproducibility
https://www.bdbiosciences.com/en-us/products/reagents/flow-cytometry-reagents/research-reagents/single-color-antibodies-ruo/percp-cy-5-5-mouse-anti-human-cd38.551400?tab=product_details
Miltenyi Biotech:
https://www.miltenyibiotec.com/DE-en/products/cd19-car-detection-reagent-human.html#130-129-550

Clinical data

Policy information about clinical studies
All manuscripts should comply with the ICMJE guidelines for publication of clinical research and a completed CONSORT checklist must be included with all submissions.

Clinical trial registration

NCT06347718

Study protocol	The full protocol was submitted. Key features as inclusion/exclusion criteria and primary/secondary endpoints can also be accessed via clinicaltrials.gov
Data collection	This single-center clinical trial was performed at Universitätsklinikum Erlangen, Germany. Data were collected from July 17th 2023 through July 11th 2025.
Outcomes	Primary endpoints were incidence and severity grading of CRS and ICANS after 4 weeks.<br>Secondary endpoints were the overall response rates at week 24 measured by disease activity composite indexes:<br>DORIS remission in SLE, 2016 ACR/EULAR moderate or major response in IIM and no progression of interstitial lung disease in SSc.<br>Additional secondary endpoints were duration of B cell depletion, persistence of CAR T cells, levels of serum autoantibodies at week 24 and expansion of CAR T cells.

Plants

Seed stocks	n/a
Novel plant genotypes	n/a
Authentication	n/a

Flow Cytometry

Plots

Confirm that:

☒ The axis labels state the marker and fluorochrome used (e.g. CD4-FITC).

☒ The axis scales are clearly visible. Include numbers along axes only for bottom left plot of group (a 'group' is an analysis of identical markers).

☒ All plots are contour plots with outliers or pseudocolor plots.

☒ A numerical value for number of cells or percentage (with statistics) is provided.

Methodology

Sample preparation	Outlined in the method section
Instrument	LSR Fortessa
Software	FlowJo v10
Cell population abundance	provided
Gating strategy	provided

☒ Tick this box to confirm that a figure exemplifying the gating strategy is provided in the Supplementary Information.

This checklist template is licensed under a Creative Commons Attribution 4.0 International License, which permits use, sharing, adaptation, distribution and reproduction in any medium or format, as long as you give appropriate credit to the original author(s) and the source, provide a link to the Creative Commons license, and indicate if changes were made. The images or other third party material in this article are included in the article's Creative Commons license, unless indicated otherwise in a credit line to the material. If material is not included in the article's Creative Commons license and your intended use is not permitted by statutory regulation or exceeds the permitted use, you will need to obtain permission directly from the copyright holder. To view a copy of this license, visit http://creativecommons.org/licenses/by/4.0/
